# *Amesia hispanica* sp. nov., Producer of the Antifungal Class of Antibiotics Dactylfungins

**DOI:** 10.3390/jof9040463

**Published:** 2023-04-12

**Authors:** Esteban Charria-Girón, Alberto Miguel Stchigel, Adéla Čmoková, Miroslav Kolařík, Frank Surup, Yasmina Marin-Felix

**Affiliations:** 1 Department Microbial Drugs, Helmholtz Centre for Infection Research (HZI), German Centre for Infection Research (DZIF), Partner Site Hannover-Braunschweig, Inhoffenstrasse 7, 38124 Braunschweig, Germany; 2 Institute of Microbiology, Technische Universität Braunschweig, Spielmannstraße 7, 38106 Braunschweig, Germany; 3 Mycology Unit, Medical School, Universitat Rovira i Virgili, C/Sant Llorenç 21, 43201 Tarragona, Spain; 4 Institute of Microbiology, Czech Academy of Sciences, Vídeňská 1083, 14220 Prague, Czech Republic

**Keywords:** antifungals, Chaetomiaceae, fungal secondary metabolites, metabolomics, Sordariales

## Abstract

During a study of the diversity of soilborne fungi from Spain, a strain belonging to the family Chaetomiaceae (Sordariales) was isolated. The multigene phylogenetic inference using five DNA loci showed that this strain represents an undescribed species of the genus *Amesia*, herein introduced as *A. hispanica* sp. nov. Investigation of its secondary metabolome led to the isolation of two new derivatives (**2** and **3**) of the known antifungal antibiotic dactylfungin A (**1**), together with the known compound cochliodinol (**4**). The planar structures of **1**–**4** were determined by ultrahigh performance liquid chromatography coupled with diode array detection and ion mobility tandem mass spectrometry (UHPLC-DAD-IM-MS/MS) and extensive 1D and 2D nuclear magnetic resonance (NMR) spectroscopy after isolation by HPLC. All isolated secondary metabolites were tested for their antimicrobial and cytotoxic activities. Dactylfungin A (**1**) showed selective and strong antifungal activity against some of the tested human pathogens (*Aspergillus fumigatus* and *Cryptococcus neoformans*). The additional hydroxyl group in **2** resulted in the loss of activity against *C. neoformans* but still retained the inhibition of *As. fumigatus* in a lower concentration than that of the respective control, without showing any cytotoxic effects. In contrast, 25″-dehydroxy-dactylfungin A (**3**) exhibited improved activity against yeasts (*Schizosaccharomyces pombe* and *Rhodotorula glutinis*) than **1** and **2**, but resulted in the appearance of slight cytotoxicity. The present study exemplifies how even in a well-studied taxonomic group such as the Chaetomiaceae, the investigation of novel taxa still brings chemistry novelty, as demonstrated in this first report of this antibiotic class for chaetomiaceous and sordarialean taxa.

## 1. Introduction

Fungal natural products represent one of the most prolific sources in the search for new antimicrobial drugs and beneficial therapeutic agents [1,2,3]. The worldwide emergence of multidrug-resistant (MDR) pathogens is now a matter of public health that requires an accelerated search for innovative and more efficient drugs, with natural products providing an excellent solution [4]. The risk due to a lack of treatment for fungal infections is as critical as it is for bacterial infections, as only four classes of antifungal drugs are currently available on the market to treat invasive mycoses (three classes) and non-systemic fungal infections (one class only) [3]. Moreover, pathogen resistance to antifungals is spreading significantly and, to some extent, the antimycotic pipeline is not as complete as it has been for the discovery of new antibacterials during the past twenty years [3,4]. The spread of acquired antifungal resistance is especially prominent in pathogens of the order Eurotiales, e.g., *Aspergillus fumigatus*, which is the main cause of invasive aspergillosis [5,6]. In addition to acquired resistance, naturally occurring resistance limits the available treatment options in some groups of fungi, such as the Mucorales [7]. Although naturally occurring resistance is not so prominent in other fungal pathogens, e.g., *Cryptococcus neoformans*, which is the leading cause of mortality in immunocompromised individuals, the loss of any one option from the few treatment options for these infections would threaten the lives of millions of people annually [8].

The order Sordariales is one of the largest and most diverse taxonomic groups of the kingdom Fungi. It is also a source of prolific producers of diverse biologically active secondary metabolites with potential applications in human therapy [9,10,11]. In the past decade, numerous examples of the isolation of bioactive secondary metabolites from these taxa have been reported, as recently summarized by Charria-Girón et al. [12]. In particular, fungi from the family Chaetomiaceae play a significant role in agriculture, ecosystems, biotechnology, and food production, as well as in animal and human health [13]. In addition, due to their mixed biosynthetic origin, species belonging to this family represent a wealth of unique and chemically diverse secondary metabolites, including alkaloids, polyketides, peptides, terpenes, and polyketide-amino acid, with more than 200 bioactive secondary metabolites reported to date [13,14].

During an ongoing project focused on the discovery of bioactive compounds from taxa belonging to the Sordariales, we discovered the production of novel derivatives of the antifungals dactylfungins by a chaetomiaceous fungus, *Amesia hispanica* sp. nov. The structures of the two new dactylfungin derivatives (**2** and **3**), together with the known dactylfungin A (**1**) and the chaetomiaceous metabolite cochliodinol (**4**), were elucidated by one-dimensional and two-dimensional nuclear magnetic resonance (1D- and 2D-NMR) spectroscopy. Details of the isolation, structure elucidation, antimicrobial activity, and cytotoxicity of each of the isolated compounds are presented herein.

## 2. Materials and Methods

### 2.1. Fungal Isolation

Soil samples were collected in Pico de Osorio (28°04′29.3″ N, 15°33′33.2″ W) in Gran Canaria, Spain. For the isolation of soilborne ascomycetes, we followed a previously described procedure to activate dormant spores [15]. In brief, approximately 1 g of each soil sample was suspended in 5 mL of 2% (*v*/*v*) phenol, shaken vigorously for 5 min, and left for 5 min. The liquid layer was discarded, and the residual soil was resuspended in 10 mL sterile water and plated onto three Petri dishes of 90 mm diameter. Melted potato carrot agar (PCA, 20 g grated potatoes, 20 g grated carrot, 20 g agar-agar, 100 mg L-chloramphenicol to avoid bacterial growth, 20 drops of 1% (*w*/*v*) dieldrin in dimethyl-ketone to avoid mites, 1 L tap water) at 50–55 °C was placed on top of the phenol-treated soil suspension and mixed by hand. All cultures were incubated at 15, 25, and 35 °C. Using a sterile needle, the ascomata of the taxonomically interesting fungi were transferred to two 55 mm-diameter Petri dishes containing oatmeal agar (OA, 30 g oatmeal flakes, 20 g agar-agar, 1 L tap water) and incubated under the same conditions.

Herbarium and ex-type material of the new species were deposited at the Westerdijk Fungal Biodiversity Institute (CBS), Utrecht, the Netherlands. An isotype is also maintained at the collection of the Facultad de Medicina y Ciencias de la Salud, University Rovira i Virgili, Reus, Spain (FMR).

### 2.2. Morphological Characterization

Phenotypic features were described from colonies growing on malt extract agar (MEA). Malt extract 20 g/L, peptone 1 g/L, and D-glucose 20 g/L, Agar 20 g/L (HiMedia, Mumbai, India), OA (Sigma–Aldrich, St. Louis, MO, USA), and PCA (HiMedia, Mumbai, India) at 25 °C. Colony colours were assessed according to The Royal Horticultural Society London [16]. Micromorphological descriptions and measurements for 30 replicates of sexual structures and relevant features were carried out in lactic acid 90%. Photomicrographs were taken with a Keyence VHX-970F microscope (Neu-Isenburg, Germany) and a Nikon eclipse Ni compound microscope, using a DS-Fi3 (Nikon, Tokyo, Japan) and NIS-Elements imaging software v. 5.20.

### 2.3. DNA Isolation, Amplification and Phylogenetic Study

DNA of the fungus was extracted and purified directly from a colony growing on yeast-malt extract agar (YM agar, malt extract 10 g/L, yeast extract 4 g/L, D-glucose 4 g/L, agar 20 g/L, pH 6.3 before autoclaving), following the Fungal gDNA Miniprep Kit EZ-10 Spin Column protocol (NBS Biologicals, Cambridgeshire, UK). The amplification of the internal transcribed spacer (ITS) regions and the large subunit (LSU) of the nuclear ribosomal RNA (rRNA) gene complex and partial fragments of the second largest subunit of DNA-directed RNA polymerase II (*rpb2*) and beta-tubulin (*tub2*) genes was performed according to White et al. [17] (ITS; primers used ITS5 ([5′-GGAAGTAAAAGTCGTAACAAGG-3′] and ITS4 [5′-TCCTCCGCTTATTGATATGC-3′]), Vilgalys and Hester [18] (LSU; primers used LROR [5′-ACCCGCTGAACTTAAGC-3′] and LR7 [5′-TACTACCACCAAGATCT-3′]), Miller and Huhndorf [19] (*rpb2*; primers used RPB2AM-1bf ([5′-CCAAGGTBTTYGTSAACGG-3′] and RPB2AM-7R [5′-GAATRTTGGCCATGGTRTCCAT-3′]) and O’Donnell and Cigelnik [20] and Groenewald et al. [21] (*tub2*; primers used T1 [5′-AACATGCGTGAGATTGTAAGT-3′] and T22 [5′-TCTGGATGTTGTTGGGAATCC-3′]). The PCR reactions were carried out using the JumpStart™ Taq ReadyMix™ (Sigma–Aldrich, St. Louis, MO, USA). PCR products were sequenced using the Sanger Cycle Sequencing method at Microsynth Seqlab GmbH (Göttingen, Germany), and the consensus sequences were obtained using Geneious^®^ 7.1.9 [22].

The phylogenetic analysis was carried out based on the combination of the four loci of our isolate and those of the type and reference strains of all species of *Amesia*, plus selected members of the Chaetomiaceae (Table 1). Each locus was aligned separately using MAFFT v. 7 [23] and manually optimized using MEGA v. 10.2.4 [24]. Loci were concatenated after checking for no conflicts [25,26]. The maximum-likelihood (ML) and Bayesian inference (BI) methods were used in a phylogenetic analysis as described by Harms et al. [10]. Bootstrap support (bs) ≥ 70% and posterior probability values (pp) ≥ 0.95 were considered significant [27]. The sequences generated in this study were deposited in GenBank (Table 1), and the alignments used in the phylogenetic analysis are included in Supplementary Material.

### 2.4. Fermentation, Extraction, and Isolation

The strain CBS 149852 was grown on YM agar at 23 °C. For the seed culture, the well-grown colonies in the agar plates were cut into small pieces using a cork borer (1 cm × 1 cm). Then, 8 pieces were added into a 500 mL Erlenmeyer flask with 200 mL of yeast malt extract broth (malt extract 10 g/L, yeast extract 4 g/L, D-glucose 4 g/L, pH 6.3 before autoclaving) and incubated at 23 °C and under shake conditions at 140 rpm. After 7 days, 6 mL of the seed culture were transferred to 10 conical flask of 500 mL with solid rice culture medium (brown rice 28 g and 0.1 L of base liquid [yeast extract 1 g/L, di-sodium tartrate di-hydrate 0.5 g/L, KH_2_PO_4_ 0.5 g/L] per flask). The cultures were incubated for 15 days in the dark at 23 °C. For the secondary metabolites extraction, the mycelia on the rice were covered with acetone, and sonicated for 30 min at 40 °C. The mycelia were separated from the acetone extract by using a filter with a cellulose filter paper (MN 615 1/4 Ø 185 mm, Macherey-Nagel GmbH & Co. KG, Düren, Germany). The extraction and filtration steps were repeated one more time. The obtained acetone extracts were combined and the acetone was evaporated in vacuo at 40 °C (evaporator: Heidolph Instruments GmbH & Co. KG, Germany; pump: Vacuubrand GmbH & Co. KG, Wertheim am Main, Germany) to yield an aqueous residue. This aqueous extract was extracted twice with an equal amount of ethyl acetate in a separation funnel. The resulting ethyl acetate fractions were combined and evaporated to dryness in vacuo at 40 °C. The obtained dry extract was dissolved in methanol, and afterwards extracted with an equal amount of heptane in a separation funnel. This step was repeated with obtained methanol phase. The methanol phase was evaporated to dryness in vacuo at 40 °C to yield 794 mg of crude extract.

The extract was pre-fractionated using flash chromatography (Grace Reveleris^®^, Columbia, MD, USA) (silica cartridge 12 g); the mobile phase consisted of A (DCM), B (acetone), and C ([DCM/acetone 8:2]:MeOH), gradient: 100% A for 5 min, increasing to 100% B in 20 min, followed by increasing to 100% solvent mixture C in 20 min and holding at 100% solvent C in 7 min). Six fractions (F1–F6) were collected, from which fraction F1 was found to contain almost solely fatty acids and was subsequently discharged.

For isolation of **4**, the fraction F2 (150 mg) was separated using a PLC 2250 preparative HPLC system (Gilson, Middleton, WI, USA) with a VP Nucleodur 100-5 C_18_ ec column (250 × 40 mm, 7 µm; Macherey-Nagel, Düren, Germany) as the stationary phase and in the following conditions: solvent A: H_2_O + 0.1% formic acid; solvent B: ACN + 0.1% formic acid; flow: 45 mL/min; gradient: from 50% B to 85% B in 45 min, then an increase from 85% B to 100% B in 12 min, to end with isocratic conditions of 100% for 15 min. This yielded compound **4** (17.47 mg, tR = 34.0–37.3 min). For isolation of compounds **1** and **3**, the fraction F4 (7 × 50 mg) was further separated using preparative as a reverse phase HPLC (Büchi, Pure C-850, 2020, Switzerland) with a Luna C_18_ (250 × 21 mm, 7 μm; Phenomenex, Torrance, CA, USA) as the stationary phase and in the following conditions: solvent A: H_2_O + 0.1% formic acid; solvent B: ACN + 0.1% formic acid; flow: 20 mL/min; gradient: from 50% B to 79% B in 12 min, then an increase from 79% B to 87% B in 35 min, and an increase from 87% B to 100% B in 10 min, to end with isocratic conditions of 100% for 10 min. This yielded compound **1** (198.94 mg, tR = 21.7–24.2 min) and compound **3** (1.5 mg, tR = 50.6–51.5 min). For isolation of **2**, the fraction F5 (2 × 45 mg) was separated using a PLC 2250 preparative HPLC system (Gilson, Middleton, WI, USA) with a Luna C_18_ (250 × 21 mm, 7 μm; Phenomenex, Torrance, CA, USA) as the stationary phase and in the following conditions: solvent A: H_2_O + 0.1% formic acid; solvent B: ACN + 0.1% formic acid; flow: 20 mL/min; gradient: from 50% B to 68% B in 15 min, then an increase from 68% B to 81% B in 40 min, and finally an increase from 81% B to 100% B in 10 min. This yielded compound **2** (2.38 mg, tR = 38.1–41.3 min).

### 2.5. Chromatography and Spectral Methods

Crude extracts and pure compounds were dissolved to a concentration of 4.5 and 1 mg/mL, respectively, in an acetone and methanol solution (1:1). Then, electrospray ionization mass (ESI-MS) spectra were recorded on an UltiMate 3000 Series uHPLC (Thermo Fischer Scientific, Waltman, MA, USA) using a C18 column (Acquity UPLC BEH 1.7 µm, 2.1 × 50 mm; Waters, Milford, MO, USA) with a sample injection volume of 2 µL, and connected to an amaZon speed ESI-Iontrap-MS (Bruker Daltonics, Bremen, Germany). The mobile phase consisted of A (H_2_O + 0.1% formic acid) and B (ACN + 0.1% formic acid) with a constant flow rate of 0.6 mL/min. The gradient began with 5% B for 0.5 min, increasing to 100% B in 20 min and holding at 100% B for 10 min. The temperature of the column was kept at 40 °C and UV-Vis data were recorded with a DAD at 190–600 nm.

UHPLC-DAD-IM-MS/MS spectra were recorded using the instrumental settings and conditions as described by Cedeño-Sanchez et al. [34]. Similarly, HRESI-MS/MS spectra were recorded with an Agilent 1200 series HPLC-UV system (Agilent Technologies, Böblingen, Germany; conditions as for ESI-MS) combined with ESI-TOF-MS (Maxis, Bruker Daltonics, Bremen, Germany), scan range 100–2500 *m*/*z*, capillary voltage 4500 V, and dry temperature 200 °C. For the crude extracts and isolated secondary metabolites, the ESI mass spectra were acquired in positive ion mode. Raw data were pre-processed with MetaboScape^®^ 2022 (Bruker Daltonics, Bremen, Germany) in the retention time range of 0.5 to 25 min. A molecular network was created with the Feature-Based Molecular Networking (FBMN) [35] on the GNPS platform [36] using the pre-processed feature table from MetaboScape. Datasets generated/analyzed for this study are included in supplementary files.

Optical rotations were recorded employing an MCP 150 circular polarimeter (Anton Paar, Seelze, Germany) at 20 °C. UV/Vis spectra were recorded with a UV-2450 spectrophotometer (Shimadzu, Kyoto, Japan). The optical rotation and UV/Vis spectra were measured for each isolated secondary metabolite in MeOH (Uvasol, Merck, Darmstadt, Germany). The 1D and 2D nuclear magnetic resonance (NMR) spectra of isolated compounds were recorded with an Avance III 700 spectrometer with a 5 mm TCI cryoprobe (Bruker, ^1^H NMR: 700 MHz, ^13^C: 175 MHz, Billerica, MA, USA) and an Avance III 500 spectrometer (Bruker, ^1^H NMR: 500 MHz, ^13^C: 125 MHz, Billerica, MA, USA). The chemical shifts δ were referenced to the solvents DMSO-*d*_6_ (^1^H, δ = 2.50; ^13^C, δ = 39.51).

### 2.6. Biological Assays

For all isolated metabolites, the antimicrobial activity was evaluated by determining the minimum inhibitory concentration (MIC) against five fungi (*Candida albicans*, *Mucor hiemalis*, *Rhodotorula glutinis*, *Schizosaccharomyces pombe*, and *Wickerhamomyces anomalus*), different Gram-positive (*Bacillus subtilis*, *Mycolicibacterium smegmatis*, and *Staphylococcus aureus*), and Gram-negative (*Acinetobacter baumannii*, *Chromobacterium violaceum*, *Escherichia coli*, and *Pseudomonas aeruginosa*) bacteria following the protocols described by Harms et al. [10]. For most bacteria, the cell suspension was prepared in Mueller–Hinton Broth (SN X927.1, Carl Roth GmbH, Karlsruhe, Germany) and was adjusted to an OD = 0.01 at 600 nm. *M. smegmatis* was cultured in 27H9 + ADC (Middlebrook 7H9 Broth Base + Middlebrook ADC Growth Supplement [SN M0678 + M0553, Merck, Darmstadt, Germany]) and its cell suspension was adjusted to an OD = 0.1 at 548 nm. The evaluated fungi were grown in MYC medium (1% bacto peptone, 1% yeast extract, 2% glycerol, pH 6.3) and the OD value was adjusted as for *M. smegmatis*. Then, for each test organism, 150 µL of the prepared suspension was added to each well of a 96-well microtiter plate. In the first row (A), 130 µL of the suspension was added, plus 20 µL of the test compounds (1 mg/mL in MeOH). For the negative control, 20 µL of MeOH was used, while different positive controls were used, depending on the test organisms. Nystatin (1 mg/mL) was used in the case of the different fungi tested. Oxytetracycline (0.1 mg/mL, *B. subtilis* 1 mg/mL) was used for all bacteria, except for *Ac. baumanii*, *M. smegmatis*, and *P. aeruginosa*, against which ciprobay (0.25 mg/mL), kanamycin (0.1 mg/mL), and gentamycin (0.1 mg/mL) were used, respectively. In this way, beginning from row A, 150 µL of the suspension was transferred to the next row, and then 150 µL was transferred to the following row. The remaining 150 µL after row H were discarded. This resulted in a serial dilution of the test compounds (66.7 µg/mL–0.52 µg/mL). The assay microplate was incubated overnight at 800 rpm on a microplate shaker. All test organisms were grown at 30 °C, except *M. smegmatis*, *E. coli*, and *P. aeruginosa*, which were grown at 37 °C. The lowest concentration of the compounds inhibiting visible growth of the test organism was selected as the MIC.

The cytotoxicity from all the isolated metabolites against two mammalian cell lines, i.e., human endocervical adenocarcinoma KB 3.1 and mouse fibroblasts L929, was evaluated in a 96-well plate following the protocols described by Harms et al. [10]. Each compound was dissolved in a manner similar to that in the previous section, and in this case epothilone B was used as the positive control. The cell lines were incubated with a serial dilution of the compounds (final range: 37 to 0.6 × 10^−3^ µg/mL) at 37 °C with 10% CO_2_ in Gibco™Dulbecco’s Modified Eagle’s Medium (SN 61965026, Thermo Fisher Scientific, Waltham, MA, USA) supplemented with 10% fetal bovine serum (SN 10500064, Thermo Fisher Scientific). After five days, the cells were stained with 3-(4,5-dimethyl-2-thiazolyl)-2,5-diphenyl-2H-tetrazolium bromide (MTT, [M2128, Sigma-Aldrich, Deisenhofen, Germany]). Living cells convert this dye to a purple derivative, the intensity of which is quantified in relation to the cells without additive (100% viability) for each concentration of the tested compounds. For quantification, a microplate reader with 595 nm was used to calculate the percentage of cell viability. From these results, the half-maximum inhibitory concentration (IC_50_) in µM was calculated.

The antifungal activity of the dactylfungins (**1**–**3**) was further evaluated against a set of relevant human pathogenic fungi belonging to three different groups: Mucorales (*Rhizopus arrhizus*), basidiomycetous yeasts (*C. neoformans*), and Eurotiales (*As. fumigatus*). Antifungal bioassays were performed according to the protocol of Štěpánek et al. [37], using amphotericin and cycloheximide as positive control. The cultivation period and final visual evaluation of plates were chosen according to recommendations for each group [38]. Mucorales and yeast-forming fungi were cultivated 24 h, while Eurotiales were cultivated for 48 h, at 25 °C on a shaker (300 rpm). The minimum inhibitory concentration for Eurotiales and Mucorales was considered at 100% inhibition, while for yeast-forming fungi the minimum inhibitory concentration was positively scored also in the case of 50% inhibition.

### 2.7. Biogeographic Study

The biogeography was studied following the workflow of Réblová et al. [39]. We used the GlobalFungi database, release 4, which contains 57,184 samples from 515 studies, 791,513,743 unique ITS1, and 2,147,483,647 unique ITS2 sequences [40]. ITS1 and ITS2 sequences were subjected to an exact-hit similarity search, which searches for sequences that are identical in both length and sequence.

## 3. Results

### 3.1. Phylogenetic Study

**Figure 1:**
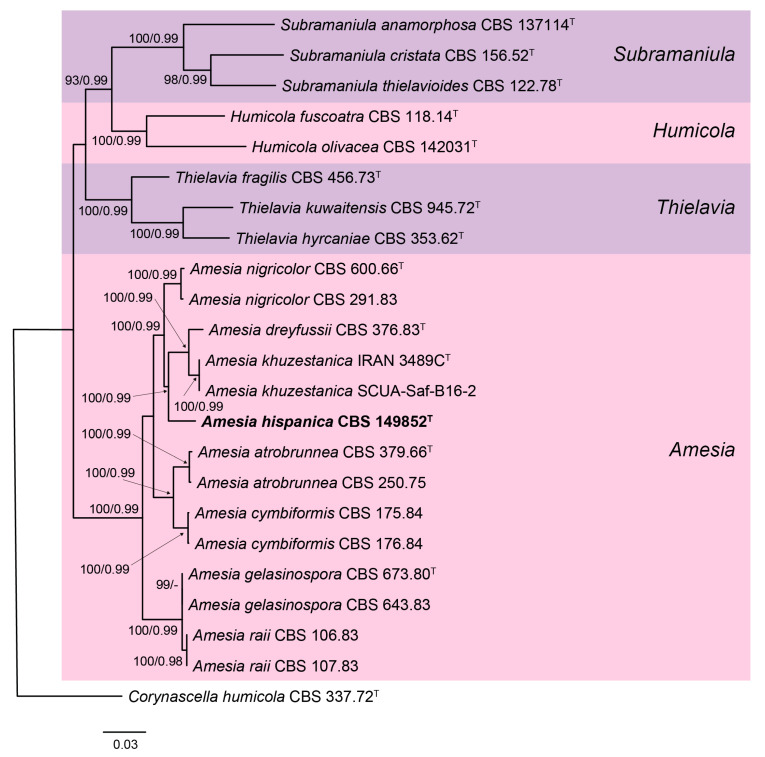
RAxML phylogram obtained from the combined ITS, LSU, *rpb2*, and *tub2* sequences of our isolate and selected strains belonging to the family Chaetomiaceae. Bootstrap support values ≥ 70/Bayesian posterior probability scores ≥ 0.95 are indicated along branches. Branch lengths are proportional to distance. The novel species is indicated in bold. Ex-type strains of the different species are indicated by ^T^.

The combined dataset consisted of 2711 bp characters, of which 591 bp corresponded to ITS, 853 bp corresponded to LSU, 521 bp corresponded to *rpb2*, and 746 bp corresponded to *tub2*. In the phylogenetic tree (Figure 1), our isolate was located in the well-supported clade (100 bs/0.99 pp) representing the genus *Amesia*. This was placed in a terminal branch, suggesting that it represented a new species. The alignment of each locus is available in the Supplementary Materials (Table S1).

### 3.2. Taxonomy

**Figure 2:**
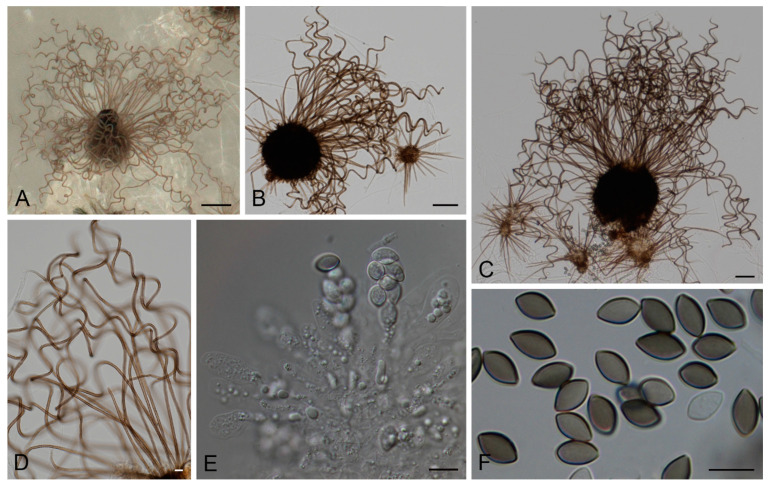
*Amesia hispanica* sp. nov. (CBS 149852). (**A**–**C**). Ascomata. (**D**). Terminal ascomatal hairs, (**E**). Asci. (**F**). Ascospores. Scale bars: (**A**) = 100 μm; (**B**,**C**) = 50 μm; (**D**–**F**) = 10 μm.

***Amesia hispanica*** Y. Marín & Stchigel, **sp. nov.** MycoBank MB847662 (Figure 2).

Etymology: “Hispanica” refers to Spain, the country where it was isolated from.

Type material: SPAIN: Gran Canaria, Pico de Osorio, from soil, 7-VIII-2009, M. Calduch and A.M. Stchigel (holotype CBS H-25223; ex-type cultures CBS 149852 = FMR 12004).

Description: Ascomata superficial, ostiolate, olivaceous to olivaceous grey under reflected light due to ascomatal hairs, subglobose to ovate, 100–200 × 85–170 μm; ascomatal wall brown, *textura angularis* or *epidermoidea* in surface view; terminal hairs first straight, then becoming undulate to loosely coiled at the top, and erect or flexuous in lower part when mature, verrucose, pale brown to brown, septate, 2.5–4.0 μm wide in the undulate or coiled upper part; lateral hairs short and straight or flexuous, or similar to the terminal hairs. Asci fasciculate, clavate, spore-bearing part 20–35 × 11–14 μm, stalks 10–16 μm long, with eight irregularly-arranged ascospores, evanescent. Ascospores one-celled, at first hyaline, then becoming pale olivaceous brown to olivaceous brown when mature, fusiform, 9–12 × 5.5–7.0 μm, with an apical germ pore at one end. Asexual morph unknown.

Culture characters: Colonies on PCA reaching 35–41 mm in one week, white with a greyed red (179C) ring surrounding the center, umbonate, velvety to cottony, margins regular to slightly fringed; reverse greyed orange (173C, D) with white to yellow white (158A–C) margins. Colonies on MEA reaching 28–30 mm in one week, yellow orange (18C) with white center, umbonate, velvety to cottony in the center, margins regular to slightly fringed; reverse yellow (11B, C). Colonies on DG18 reaching 16–20 mm in one week, white, umbonate, cottony, slightly lobulate, margins regular to slightly fringed; reverse yellow orange (20C, D). Colonies on OA reaching 40–43 mm in one week, greyed green (197B–D) with white to greyed white mycelia (156D), umbonate, cottony, margins regular to slightly fringed, reverse greyed green (197B).

Biogeography: The ITS sequence is identical (Genbank Accession HQ316557 [41]) or 99.8% similar (KT357692, 524/525 bp, unpublished) with two strains isolated from an unknown source in India. According to GlobalFungi, identical sequences were found in a single air sample from Madrid, Spain [42], and in two soil samples from Woody Island in the South China Sea, China [43]. Data from NCBI Genbank and GlobalFungi database show that *A. hispanica* is probably a rare fungus, found not only in Spain, but also in Asia (India, South China).

Notes: Morphologically, *A. hispanica* is similar to *A. gelasinospora* and *A. raii*, all of which bear coiled hairs surrounding the ascoma ostiole and produce fusiform or broadly fusiform ascospores. However, *A. gelasinospora* and *A. raii* are characterized by ascomata densely ornamented with hairs. *Amesia gelasinospora* and *A. hispanica* have been both isolated from soil samples, while *A. raii* has been found on the wood of the mango tree (*Mangiferae indicae*) and stored wheat grains. In our phylogenetic tree (Figure 1), *A. gelasinospora* and *A. raii* clustered in a monophyletic well-supported clade (100 bs/0.99 pp), suggesting that these could represent the same species. However, only sequences of *rpb2* of *A. raii* are available; thus, other loci need to be sequenced to determine whether these represent the same species. The species differ in the length of the ascospores [44,45], but this could be due to the difference in the culture media and conditions where these were described. *Amesia hispanica* is not phylogenetically related to the aforementioned species, but it is phylogenetically related to *A. dreyfussii* and *A. khuzestanica*. However, these later species produce ascomata with straight to flexuous terminal hairs surrounding the ostiole [31,45]. Moreover, *A. khuzestanica* can be easily distinguished from the other species of the genus by its reniform ascospores [31].

Key to species of *Amesia*
1. Reniform ascospores.............................................................................................*Amesia khuzestanica*
1. Ovate, fusiform or pyriform ascospores............................................................................................2
2. Ascomata with straight or flexuous hairs surrounding the ostiole................................................3
2. Ascomata with coiled hairs surrounding the ostiole.......................................................................5
3. Ascospores ovate to ellipsoidal, (7.0–)8.0–9.0(–9.5) × (5.5–)6.0–6.5(–7.0) μm..........*A. cymbiformis*
3. Ascospores fusiform or pyriform.......................................................................................................4
4. Ascospores (8.0–)8.5–10.5(–11.0) × 4.5–5.5(–6.0) μm...................................................*A. atrobrunnea*
4. Ascospores 14–16 × 5–6 μm...............................................................................................*A. dreyfussii*
5. Ascospores ovate, 6.0–7.0(–7.5) × 4.0–5.0(–5.5) μm........................................................*A. nigricolor*
5. Ascospores fusiform or broadly fusiform.........................................................................................6
6. Ascomata not densely ornamented with hairs, ascospores 9.0–12.0 × 5.5–7.0 μm.....*A. hispanica*
6. Ascomata densely ornamented with hairs.......................................................................................7
7. Ascospores 8.5–10 × 5.5–6.5 μm.................................................................................*A. gelasinospora*
7. Ascospores 10–15 × 5–7.5 μm....................................................................................................*A. raii*

### 3.3. Isolation and Structure Elucidation of Secondary Metabolites

After the solid cultivation of *A. hispanica* sp. nov. (CBS 149852) in rice medium, 794 mg of crude extract were obtained. Targeted isolation by preparative HPLC of the active fractions obtained after silica pre-fractionation afforded compounds **1**−**4** (Figure 3). Their structures were elucidated by 1D- and 2D-NMR spectroscopy in combination with tandem mass spectrometry analyses (Supplementary Figures S5–S15).

**Figure 4:**
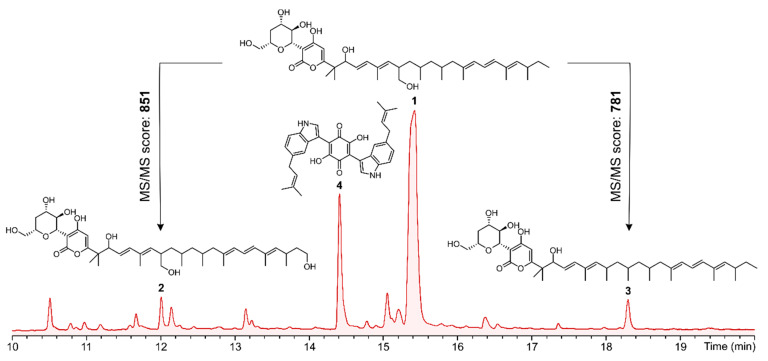
HPLC-UV/Vis chromatogram (210 nm) of the crude extract from the rice culture of *A. hispanica* sp. nov. with peaks of isolated compounds indicated by bold numbers referring to the depicted molecules (**1**–**4**). Over the arrows, the MS/MS similarity scores between MS/MS spectra from compounds **2** and **3** and the MS/MS spectrum of **1** are shown. Complete HPLC-UV/Vis chromatogram (210 nm) from 0 to 25 min is shown in Figure S20.

Compound **1** was obtained as a colorless amorphous powder and its molecular formula was established as C_41_H_64_O_9_ (ten degrees of unsaturation) according to the quasimolecular ion peak cluster at *m*/*z* 701.46257 [M + H]^+^ in the HRESI-MS spectrum. Comparison ^1^H and ^13^C NMR data of **1** measured in DMSO-*d*_6_ with those of Xaio et al. [46] confirmed its identity as dactylfungin A, a known antifungal antibiotic containing an α-pyrone ring conjoined with a polyalcohol moiety and a long side chain. An MS/MS similarity search in MetaboScape for dactylfungin A (**1**) against all the identified features in the crude extract yielded an MS/MS score > 750 for compounds **2** and **3**, as well as other minor derivatives observed in the dactylfungin molecular family (MF) (Figure 4 and Figures S1–S4). Thus, this MF consisted of 16 consensus spectra (nodes), including compounds **2** and **3**, each of which differed by the addition and loss of one oxygen atom when compared to **1**; consequently, we embarked on their preparative isolation.

In this way, compound **2** was obtained as a colorless amorphous powder. The molecular ion cluster at *m*/*z* 717.45596 [M + H]^+^ in the HRESI-MS spectrum indicated that the molecular formula is C_41_H_64_O_10_, and thereby ten degrees of unsaturation. The key difference in the NMR spectra between **1** and **2** was the replacement of the terminal methyl CH_3_–21″ by an oxymethylene moiety. Consequently, **2** was assigned as 21″-Hydroxy-dactylfungin A. Compound **3** was also obtained as a colorless amorphous powder and its molecular formula was established as calculated for C_41_H_64_O_8_ (ten degrees of unsaturation), according to the quasimolecular ion peak cluster at *m*/*z* 685.46734 [M + H]^+^ in the HRESI-MS spectrum. The key difference was identified as the replacement of oxymethylene CH_2_–25″ by an additional methyl group. Thus, we assigned **3** as 25″-Dehydroxy-dactylfungin A.

Compound **4** was obtained as a dark purple powder and its molecular formula was established as calculated for C_32_H_30_N_2_O_4_ (nineteen degrees of unsaturation), according to the quasimolecular ion peak cluster at *m*/*z* 507.22760 [M + H]^+^ in the HRESI-MS spectrum. Compound **4** was identified, based on ^1^H and ^13^C NMR data, as cochliodinol.

### 3.4. Physico-Chemical Characteristics of the Isolated Metabolites

#### 3.4.1. Dactylfungin A (**1**)

Colorless amorphous powder; [α]_D_^20^ +7 (c 0.001, MeOH); UV (MeOH) λmax (log ε) 278.5 (4.43), 236 (4.69); ^1^H NMR data (500 MHz, DMSO-*d*_6_): δ_H_ 6.39 (dd, *J* = 15.3, 10.8 Hz, 15″–H), 6.30 (d, *J* = 15.6 Hz, 4″–H), 6.22 (d, *J* = 15.3 Hz, 16″–H), 6.06 (s, 5–H), 5.91 (d, *J* = 10.8 Hz, 14″–H), 5.59 (dd, *J* = 15.6, 7.0 Hz, 3″–H), 5.39 (d, *J* = 9.6 Hz, 6″–H), 5.33 (d, *J* = 9.6 Hz, 18″–H), 5.13 (br d, *J* = 4.7 Hz, 2″–OH), 4.30 (d, *J* = 9.6 Hz, 1′–H), 4.24 (dd, *J* = 6.3, 4.7 Hz, 2″–H), 4.02 (dd, *J* = 9.6, 8.7 Hz, 2′–H), 3.39–3.49 (m, 3′–H, 5′–H, 6–H_a_), 3.36 (br d, *J* = 5.6 Hz, 25″–H_2_), 3.34 (m, 6–H_b_), 2.64 (m, 7″–H), 2.48 (m, 19″–H), 2.13 (dd, *J* = 12.3, 3.6 Hz, 12″–H_a_), 1.91 (dd, *J* = 11.4, 4.7 Hz, 4′–H_a_), 1.83 (d, *J* = 0.6 Hz, 29″–H_3_), 1.81 (br s, 24″–H_3_), 1.79 (br s, 28″–H_3_), 1.77 (m, 11″–H_3_), 1.73 (m, 12″–H_b_), 1.58 (m, 9″–H), 1.56 (m, 8″–H_a_) ^x1^, 1.44 (m, 20″–H_a_), 1.23–1.36 (m, 4′–H_b_, 10″–H_a_, 20″–H_b_), 1.17 (s, 22″–H_3_), 1.13 (s, 23″–H_3_), 1.56 (m, 8″–H_b_)^x1^, 1.02 (d, *J* = 6.6 Hz, 30″–H_3_), 0.98 (m, 10″–H_b_), 0.95 (d, *J* = 6.2 Hz, 26″–H_3_), 0.89 (t, *J* = 7.4 Hz, 21″–H_3_), 0.85 (d, *J* = 6.3 Hz, 27″–H_3_); ^13^C NMR data (125 MHz, DMSO-*d*_6_): δ_C_ 169.9 (C, C–6), 168.1 (C, C–4), 163.7 (C, C–2), 138.7 (CH, C–18″), 137.0 (C, C–13″), 136.9 (CH, C–4″), 136.6 (CH, C–6″), 136.0 (CH, C–16″), 133.1 (C, C–17″), 133.0 (C, C–5″), 127.1 (CH, C–14″), 126.7 (CH, C–3″), 123.2 (CH, C–15″), 99.6 (C, C–3), 99.3 (CH, C–5), 77.1 (CH, C–5′), 76.1 (CH, C–2″), 74.2 (CH, C–1′), 73.1 (CH, C–3′), 71.7 (CH, C–2′), 65.1 (CH_2_, C–25″), 64.7 (CH_2_, C–6′), 47.7 (CH_2_, C–12″), 45.0 (CH_2_, C–10″), 44.2 (C, C–1″), 40.1 (CH_2_, C–8″) ^×1^, 39.2 (CH, C–7″), 36.9 (CH_2_, C–4′), 34.3 (CH, C–19″), 30.4 (CH_2_, C–20″), 28.5 (CH, C–11″), 28.1 (CH, C–9″), 23.1 (CH_3_, C–23″), 21.6 (CH_3_, C–26″), 21.1 (CH_3_, C–30″), 20.7 (CH_3_, C–27″), 20.1 (CH_3_, C–22″), 16.9 (CH_3_, C–28″), 13.4 (CH_3_, C–24″), 13.0 (CH_3_, C–29″), 12.3 (CH_3_, C–21″) ppm; high-resolution electrospray ionization mass spectrometry (HRESI-MS): *m*/*z* 701.46257 [M + H]^+^ (calculated for C_41_H_64_O_9_, 700.45530); CCS (collisional cross section in Å^2^) = 277.6; MS/MS spectrum (Figure S1). ^×1^ Data extracted from HSQC & HMBC spectra.

#### 3.4.2. 21″-Hydroxy-dactylfungin A (**2**)

Colorless amorphous powder; [α]_D_^20^ +6 (c 0.001, MeOH); UV (MeOH) λmax (log ε) 278.5 (4.40), 236.5 (4.69); ^1^H NMR data (700 MHz, DMSO-*d*_6_): δ_H_ 6.29 (dd, *J* = 15.4, 10.9 Hz, 15″–H), 6.30 (d, *J* = 15.6 Hz, 4″–H), 6.12 (d, *J* = 15.3 Hz, 16″–H), 5.92 (br s, 5–H), 5.81 (d, *J* = 10.9 Hz, 14″–H), 5.50 (dd, *J* = 15.6, 7.0 Hz, 3″–H), 5.29 (d, *J* = 9.6 Hz, 6″–H), 5.24 (d, *J* = 9.6 Hz, 18″–H), 5.01 (d, *J* = 5.0 Hz, 2″–OH), 4.19 (d, *J* = 9.6 Hz, 1′–H), 4.14 (dd, *J* = 6.3, 4.7 Hz, 2″–H), 3.92 (dd, *J* = 9.6, 8.7 Hz, 2′–H), 3.28–3.37 (m, 3′–H, 5′–H, 6–H_a_, 21–H_2_), 3.26 (m, 25″–H_2_), 3.24 (m, 6–H_b_), 2.61 (m, 19″–H), 2.54 (m, 7″–H), 2.04 (dd, *J* = 13.0, 4.4 Hz, 12″–H_a_), 1.81 (dd, *J* = 11.6, 5.2 Hz, 4′–H_a_), 1.73 (d, *J* = 0.9 Hz, 29″–H_3_), 1.71 (d, *J* = 0.9 Hz, 24″–H_3_), 1.70 (br s, 28″–H_3_), 1.67 (m, 11″–H_3_), 1.63 (m, 12″–H_b_), 1.49 (m, 9″–H), 1.46 (m, 20″–H_a_), 1.46 (m, 8″–H_a_), 1.36 (m, 10″–H_b_), 1.19 (m, 10″–H_a_), 1.17 (m, 4′–H_b_), 1.07 (s, 22″–H_3_), 0.93 (s, 8″–H_b_), 0.92 (d, *J* = 6.6 Hz, 30″–H_3_), 0.89 (m, 10″–H_b_), 0.86 (d, *J* = 6.5 Hz, 26″–H_3_), 0.85 (d, *J* = 6.3 Hz, 27″–H_3_); ^13^C NMR data (125 MHz, DMSO-*d*_6_): δ_C_ 169.2 (C, C–6), 168.1 (C, C–4), 163.4 (C, C–2), 138.4 (CH, C–18″), 136.6 (C, C–13″), 136.4 (CH, C–4″), 136.1 (CH, C–6″), 135.5 (CH, C–16″), 132.6 (C, C–17″), 132.5 (C, C–5″), 126.6 (CH, C–14″), 126.3 (CH, C–3″), 122.8 (CH, C–15″), 99.3 (CH, C–5), 98.9 (C, C–3), 76.6 (CH, C–5′), 75.6 (CH, C–2″), 73.9 (CH, C–1′), 72.6 (CH, C–3′), 71.3 (CH, C–2′), 64.6 (CH_2_, C–25″), 64.2 (CH_2_, C–6′), 58.9 (CH_2_, C–21″), 47.2 (CH_2_, C–12″), 44.6 (CH_2_, C–10″), 43.7 (C, C–1″), 40.3 (CH_2_, C–20″), 39.4 × ^1^ (CH_2_, C–8″), 38.7 (CH, C–7″), 36.4 (CH_2_, C–4′), 28.8 (CH, C–19″), 28.0 (CH, C–11″), 27.6 (CH, C–9″), 22.7 (CH_3_, C–23″), 21.1 (CH_3_, C–26″), 21.0 (CH_3_, C–30″), 20.2 (CH_3_, C–27″), 19.7 (CH_3_, C–22″), 16.5 (CH_3_, C–28″), 13.0 (CH_3_, C–24″), 12.5 (CH_3_, C–29″) ppm; ESI-MS: *m*/*z* 715.51 [M–H]^−^, 699.44 [M–H_2_O + H]^+^, 717.44 [M + H]^+^ and 739.47 [M + Na]^+^; HRESI-MS: *m*/*z* 717.45596 [M + H]^+^ (calculated for C_41_H_64_O_10_, 716.44868); CCS (collisional cross section in Å^2^) = 280.2; MS/MS spectrum (Figure S2). ^×1^ Data extracted from HSQC & HMBC spectra.

#### 3.4.3. 25″-Dehydroxy-dactylfungin A (**3**)

Colorless amorphous powder; [α]_D_^20^ +5 (c 0.001, MeOH); UV (MeOH) λmax (log ε) 278.5 (4.27), 236.5 (4.41); ^1^H NMR data (700 MHz, DMSO-*d*_6_): δ_H_ 6.29 (dd, *J* = 15.3, 10.9 Hz, 15″–H), 6.17 (d, *J* = 15.6 Hz, 4″–H), 6.13 (d, *J* = 15.3 Hz, 16″–H), 5.81 (d, *J* = 10.9 Hz, 14″–H), 5.50 (dd, *J* = 15.6, 7.0 Hz, 3″–H), 5.27 (d, *J* = 9.6 Hz, 6″–H), 5.24 (d, *J* = 9.6 Hz, 18″–H), 4.95 (br d, *J* = 3.9 Hz, 2″–OH), 4.61 (br s, 6′–H), 4.18 (d, *J* = 9.6 Hz, 1′–H), 4.15 (dd, *J* = 6.3, 4.7 Hz, 2″–H), 3.92 (dd, *J* = 9.6, 8.7 Hz, 2′–H), 3.30–3.38 (m, 3′–H, 5′–H, 6–H_a_), 3.24 (m, 6–H_b_), 2.54 (m, 7″–H), 2.38 (m, 19″–H), 2.03 (m, 12″–H_a_), 1.81 (dd, *J* = 11.8, 5.0 Hz, 4′–H_a_), 1.74 (s, 29″–H_3_), 1.70 (br s, 28″–H_3_), 1.69 (br s, 24″–H_3_), 1.68 (m, 11″–H_3_), 1.68 (m, 12″–H_b_), 1.50 (m, 9″–H), 1.34 (m, 20″–H_a_), 1.11–1.26 (m, 4′–H_b_, 8″–H_a_, 10″–H_a_, 20″–H_b_), 1.06 (s, 22″–H_3_), 1.00 (s, 23″–H_3_), 0.99 (m, 8″–H_b_), 0.92 (d, *J* = 6.6 Hz, 30″–H_3_), 0.91 (m, 10″–H_b_), 0.90 (d, *J* = 6.5 Hz, 25″–H_3_), 0.85 (d, *J* = 6.5 Hz, 26″–H_3_), 0.80 (t, *J* = 7.4 Hz, 21″–H_3_), 0.76 (d, *J* = 5.8 Hz, 27″–H_3_); ^13^C NMR data (175 MHz, DMSO-*d*_6_): δ_C_ 168.2 (C, C–6), 163.7 (C, C–2), 139.2 (CH, C–6″), 138.3 (CH, C–18″), 136.5 (C, C–13″), 136.0 (CH, C–4″), 135.5 (CH, C–16″), 132.6 (C, C–17″), 130.7 (C, C–5″), 126.6 (CH, C–14″), 126.5 (CH, C–3″), 122.8 (CH, C–15″), 97.8 (C, C–3), 76.4 (CH, C–5′), 75.4 (CH, C–2″), 74.4 (CH, C–1′), 72.6 (CH, C–3′), 71.5 (CH, C–2′), 64.2 (CH_2_, C–6′), 47.4 (CH_2_, C–12″), 44.7 (CH_2_, C–10″), 44.2 (C, C–1″), 44.5 (CH_2_, C–8″), 43.5 (CH, C–7″), 36.5 (CH_2_, C–4′), 33.8 (CH, C–19″), 29.9 (CH_2_, C–20″), 29.6 (CH, C–11″), 27.4 (CH, C–9″), 22.3 (CH_3_, C–23″), 20.7 (CH_3_, C–30″), 20.49 (CH_3_, C–25″), 20.45 (CH_3_, C–26″), 20.1 (CH_3_, C–27″), 20.0 (CH_3_, C–22″), 16.4 (CH_3_, C–28″), 12.61 (CH_3_, C–24″), 12.56 (CH_3_, C–29″), 11.8 (CH_3_, C–21″) ppm; ESI-MS: *m*/*z* 715.51 [M–H]^−^, 667.48 [M–H_2_O + H]^+^, 685.44 [M + H]^+^ and 707.47 [M + Na]^+^; HRESI-MS: *m*/*z* 685.46734 [M + H]^+^ (calculated for C_41_H_64_O_8_, 684.46006); CCS (collisional cross section in Å^2^) = 272.2; MS/MS spectrum (Figure S3).

#### 3.4.4. Cochliodinol (**4**)

Dark purple powder; ^1^H-NMR and ^13^C-NMR were in good agreement with literature [47]; HRESI-MS: *m*/*z* 507.22760 [M + H]^+^ (calculated for C_32_H_30_N_2_O_4_, 506.22032); CCS (collisional cross section in Å^2^) = 238.2.

### 3.5. Biological Activities

All isolated secondary metabolites showed antimicrobial activity against different fungi and/or different bacteria (Table 2). The antifungal activity of the isolated dactylfungins (**1**−**3**) was also evaluated against *R. arrhizus*, *C. neoformans*, and *As. fumigatus*, as they did not show any significant cytotoxic effects when compared to compound **4**. Among them, compounds **1** and **2**, when compared to the respective control, showed better activities for *As. fumigatus* (Table 2), and similar potency against *C. neoformans* for compound **1**. On the other hand, compound **3** displayed inhibitory effects against *Rh. glutinis* and *Sc. pombe* comparable to that of the controls, but also exhibited slight cytotoxicity against the tested cell lines.

## 4. Discussion

The genus *Amesia* was introduced by Wang et al. [14], based on a phylogenetic analysis using the concatenated ITS, LSU, *rpb2*, and *tub2* sequences. This genus was erected to accommodate four species of *Chaetomium* located far from the monophyletic clade including the types of species of the genus, *Chaetomium globosum*. In a recent study, two other species of *Chaetomium* were transferred to *Amesia* [30]. Moreover, *A. khuzestanica* was introduced to accommodate an isolate associated with necrotic spots of the leaves of *Albizia lebbeck* in Iran [31]. In the present study, a fungus isolated from soil in Spain resulted in a new species of this genus. Eight species were then included in this genus, which are distributed worldwide and exhibit a high morphological diversity in their ascomatal hairs and ascospores [14,30,31]. Our new species can be easily distinguished by ascomata that are sparsely covered with coiled hairs.

Taxa belonging to the Chaetomiaceae have been extensively studied for the production of bioactive secondary metabolites and different biotechnological applications. In particular, metabolites from different chemical classes, such as benzoquinones, cytochalasans, diketopiperazines, natphtaquinones, sumiki’s acid derivatives, and diketopiperazines, have been reported for the genus *Amesia* [13,14]. In this study, two novel derivatives of dactylfungin A (**1**)—21″-Hydroxy-dactylfungin A (**2**) and 25″-Dehydroxy-dactylfungin A (**3**)—were found to be produced by the new species *A. hispanica*. These antifungal antibiotics contain an α-pyrone motif substituted with a polyalcohol and a long fatty acid chain. Indeed, the α-pyrone structural feature is widespread among metabolites from different sources, including animals, bacteria, fungi, insects, and marines organisms that display several biological activities, such as antifungal, antibacterial, cytotoxic, neurotoxic, and phytotoxic [48,49]. Despite this fact, derivatives resembling the dactylfungin compound family are rarely found and only a few other examples have been reported from fungi (Figure S20) [46,50,51,52,53]. Here, dactylfungin A (**1**) exhibited an antimicrobial activity similar to that originally reported for dactylfungin B [46]. Dactylfungin A did not show any antibacterial activity, but did show potent antifungal effects against *C. neoformans* and *As. fumigatus* (MIC = 6.25 µg/mL). The presence of an additional hydroxyl group at C-21 for compound **2** resulted in a 4-fold increase in the MIC value against *As. fumigatus*, a loss of activity against *C. neoformans*, and weak activity against *B. subtilis*. However, it is still worth noting that the MIC value against *As. fumigatus* for compound **2** was lower than that of the respective control and there was no evidence of cytotoxic effects for this compound against the tested cell lines. Similarly, the absence of a hydroxyl group at C-25 for compound **3** caused the loss of activity against *As. fumigatus* and *C. neoformans*, but resulted in the appearance of potent inhibitory effects against *Sc. pombe* and *Rh. glutinis* (MIC = 4.2 µg/mL) and weak activity against *Mu. hiemalis*. In addition, weak cytotoxic effects against the two different mammalian cell lines tested were found for compound **3**.

Fusapyrones represent an example of metabolites related to the dactylfungin, which were originally isolated from *Fusarium semitectum* [50,51,54,55]. These compounds showed substantial antifungal inhibitory activities toward agents of human mycoses. Recently, the structure–activity relationships of derivatives of fusapyrone have been studied by Altomare et al. [51], and only fusapyrone and deoxyfusaropyrone showed considerable antifungal activity against moulds, low zootoxicity and selective action. Among others, pentaacetylation of fusapyrone resulted in a significant increase in toxicity, which was mainly attributed to a decrease in hydrophobicity. Similar congeners (YM-202204 and S39163/F-1) have been reported from the fungus *Phoma* sp., from which broad spectrum antifungal activity and inhibition of glycosyl-phosphatidyl-inositol (GPI)-anchoring in yeast cells was found for YM-202204 [52]. 

The comparison between the activities of the different dactylfungin derivatives illustrates that the hydroxylation pattern of the side chain plays an important role in the antifungal activity of these molecules, while the substitution of the α-pyrone with a γ-pyrone ring (dactylfungin B) does not account for the same effect. This change in activity might also be attributed to the effect of the hydroxylation pattern on the hydrophobicity. Limited knowledge is available about the biosynthesis of this group of compounds due among other reasons to the chemical complexity attributed by the presence of rare features such as a C-glycosylated 4-deoxyglucose, a gem-dimethyl group, and an a-β to β-γ double bond shift at C-20 in the case of fusaropyrone. For instance, the polyketide synthase (PKS) FmPKS40 seems to be involved in fusapyrone biosynthesis, a compound that is likely synthesized from acetyl-CoA as a starter unit and the consequent addition of 10 malonyl-CoA units by successive Claisen-condensations [56]. However, details on the synthesis of the gem-dimethyl group and the C-glycosylation mechanism for this type of compound remain unclear. Futures studies will be required to clarify the biosynthesis of this class of metabolites. Thus, the discovery of the production of the dactylfungins by a member of the Sordariales could serve as a starting point for this purpose.

Together with the dactylfungins (**1**–**3**), the known compound cochliodinol (**4**) was isolated as a major metabolite. This group of compounds are, to a certain extent, ubiquitous within the Chaetomiaceae. Wang et al. [14] studied the production of cochliodinol and isocochliodinol by representatives of different genera of the Chaetomiaceae, finding the production of this group of compounds by almost all genera tested, i.e., *Amesia*, *Botryotrichum*, *Chaetomium*, *Dichotomopilus*, *Ovatospora*, and *Subramaniula*. Compound **4** showed weak inhibition of *B. subtillis* and *St. aureus*. Rather strong cytotoxic effects were also found for this compound against the tested cell lines.

The Chaetomiaceae represent a wealthy source of secondary metabolites, which are of important interest for the pharmaceutical industry in the development and study of leading drug candidates. Even within well-studied phylogenetic groups such as this one, the chances to expand the chemical inventory of these fungi and discover chemistry novelty from unexplored taxa are plentiful, as demonstrated herein by the first report of the antifungal antibiotics dactylfungins in a member of the Sordariales.

## Figures and Tables

**Figure 3:**
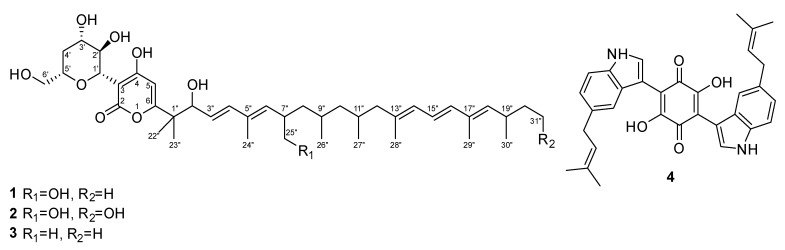
Chemical structures of the isolated metabolites (**1**–**4**).

**Table 1:** Isolates and reference strains of the order Sordariales included in the phylogenetic study. Novel taxon and sequences generated in this study are indicated in bold.

Taxa	Strain	GenBank Accession Number				References
		LSU	ITS	*rpb2*	*tub2*	
*Amesia atrobrunnea*	CBS 379.66 ^T^	JX280666	JX280771	KX976798	KX976916	[14,28]
	CBS 250.75	MH872650	KX976575	KX976799	KX976917	[14,29]
*A. cymbiformis*	CBS 175.84	MH873429	KX976576	KX976799	KX976917	[14,29]
	CBS 176.84	MH873430	KX976577	KX976800	KX976918	[14,29]
*A. dreyfussii*	CBS 376.83^T^	MH873331	MH861613	MZ342985	MZ343023	[29,30]
*A. khuzestanica*	IRAN 3489C^T^	-	MT551117	MN275706	MN275701	[31]
	SCUA-Saf-B16-2	-	MT551118	MN275707	MN275702	[31]
*A. nigricolor*	CBS 600.66^T^	MH870559	KX976578	KX976802	KX976920	[14,29]
	CBS 291.83	MH873317	KX976579	KX976803	KX976921	[14,29]
*A. gelasinospora*	CBS 673.80^T^	MH873069	KX976580	KX976804	KX976922	[14,29]
	CBS 643.83	KX976706	KX976581	KX976805	KX976923	[14]
***A. hispanica***	**CBS 149852^T^**	**OQ100832**	**OQ100090**	**OQ108867**	**OQ108868**	**Present study**
*A. raii*	CBS 107.83^T^	-	-	MZ342968	-	[30]
	CBS 106.83	-	-	MZ342967	-	[30]
*Corynascella humicola*	CBS 337.72^T^	MH872209	KX976656	KX976850	KX976998	[14,29]
*Humicola fuscoatra*	CBS 118.14^T^	MH866152	KX976675	KX976882	KX977017	[14,29]
*H. olivacea*	CBS 142031^T^	KX976770	KX976676	KX976883	KX977018	[14]
*Subramaniula anamorphosa*	CBS 137114^T^	KP970641	KP862598	KP900667	KP900704	[32]
*S. cristata*	CBS 156.52^T^	KX976788	KX976690	KX976903	KX977038	[14]
*S. thielavioides*	CBS 122.78^T^	KP970654	KP862597	KP900670	KP900708	[32]
*Thielavia fragilis*	CBS 456.73^T^	KX976791	KX976693	KX976907	KX977042	[14]
*T. hyrcaniae strain*	CBS 353.62^T^	KM655368	KM655329	KX976908	KX977043	[14,33]
*T. kuwaitensis strain*	CBS 945.72^T^	MH872326	KM655332	KX976909	KX977044	[14,29,33]

CBS: Westerdijk Fungal Biodiversity Institute, Utrecht, the Netherlands; IRAN: Iranian Fungal Culture Collection, Iranian Research Institute of Plant Protection, Tehran, Iran; SCUA: Collection of Fungal Cultures, Department of Plant Protection, Shahid Chamran University of Ahvaz, Iran. ^T^ indicates ex-type strains.

**Table 2:** Minimum inhibitory concentration (MIC, µg/mL) against bacterial and fungal test organisms, and half-maximal inhibitory concentrations (IC_50_, µM) against mammalian cell lines of compounds **1**–**4**. ^1^ cycloheximide, ^2^ nystatin, ^3^ ciprobay, ^4^ oxytetracycline, ^5^ kanamycin, ^6^ gentamicin, ^7^ epothilone B, -: no inhibition observed under test conditions, nt: not tested.

Bioassay	Test Organism/Cell Line	Compound				Positive Control
		1	2	3	4	
**MIC (µg/mL)**	*Rhizopus arrhizus*	100	100	50	nt	16.65 ^1^
	*Cryptococcus neoformans* *	6.25	-	-	nt	2.08 ^1^
	*Aspergillus fumigatus*	6.25	25	100	nt	33.3 ^1^
	*Schizosaccharomyces pombe*	-	-	4.2	-	8.3 ^2^
	*Wickerhamomyces anomalus*	-	-	-	-	8.3 ^2^
	*Mucor hiemalis*	-	-	66.6	-	8.3 ^2^
	*Can* *dida albicans*	-	-	-	-	8.3 ^2^
	*Rhodotorula glutinis*	66.6	-	4.2	66.6	4.2 ^2^
	*Acinetobacter baumanii*	-	-	-	-	0.26 ^3^
	*Escherichia coli*	-	-	-	-	1.7 ^4^
	*Bacillus subtilis*	-	66.6	-	66.6	8.3 ^4^
	*Mycobacterium smegmatis*	-	-	-	-	1.7 ^5^
	*Staphylococcus aureus*	-	-	-	-	0.42 ^4^
	*Pseudomonas aeruginosa*	-	-	-	-	0.21 ^6^
	*Chromobacterium violaceum*	-	-	-	-	0.42 ^4^
**IC_50_ (µM)**	L929	-	-	32.1	5.5	0.00134 ^7^
	KB 3.1	-	-	19.0	4.9	0.00006 ^7^

* The minimum inhibitory concentration (MIC) against *Cryptococcus neoformans* was positively scored in case of 50% inhibition. MIC against other microorganisms was considered positive at 100% inhibition.

## Data Availability

The DNA sequences are deposited in GenBank (https://www.ncbi.nlm.nih.gov/genbank/) and all other relevant data are included in the Supplementary Information.

## Supplementary Materials

The following supporting information can be downloaded at: https://www.mdpi.com/article/10.3390/jof9040463/s1, Figure S1: MS/MS spectrum of dactylfungin (**1**), Figure S2: MS/MS spectrum of 21″-Hydroxy-dactylfungin A (**2**), Figure S3: MS/MS spectrum of 25″-Dehydroxy-dactylfungin A (**3**), Figure S4: Dactylfungin molecular family created from the solid rice medium extract of the culture of *Amesia hispanica* sp. nov., Figure S5: ^1^H NMR spectrum (500 MHz, DMSO-*d*_6_) of dactylfungin (**1**), Figure S6: ^13^C NMR spectrum (125 MHz, DMSO-*d*_6_) of dactylfungin (**1**), Figure S7: COSY NMR spectrum (500 MHz, DMSO-*d*_6_) of dactylfungin (**1**), Figure S8: HSQC NMR spectrum (500 MHz, DMSO-*d*_6_) of dactylfungin (**1**), Figure S9: HMBC NMR spectrum (500 MHz, DMSO-*d*_6_) of dactylfungin (**1**), Figure S10: ^1^H NMR spectrum (700 MHz, DMSO-*d*_6_) of 21″-Hydroxydactylfungin (**2**), Figure S11: ^13^C NMR spectrum (175 MHz, DMSO-*d*_6_) of 21″-Hydroxydactylfungin (**2**), Figure S12: COSY NMR spectrum (700 MHz, DMSO-*d*_6_) of 21″-Hydroxydactylfungin (**2**), Figure S13: HSQC NMR spectrum (700 MHz, DMSO-*d*_6_) of 21″-Hydroxydactylfungin (**2**), Figure S14: HMBC NMR spectrum (700 MHz, DMSO-*d*_6_) of 21″-Hydroxydactylfungin (**2**), Figure S15: ^1^H NMR spectrum (700 MHz, DMSO-*d*_6_) of 25″-Dehydroxydactylfungin (**3**), Figure S16: ^13^C NMR spectrum (175 MHz, DMSO-*d*_6_) of 25″-Dehydroxydactylfungin (**3**), Figure S17: COSY NMR spectrum (700 MHz, DMSO-*d*_6_) of 25″-Dehydroxydactylfungin (**3**), **Figure S18**: HSQC NMR spectrum (700 MHz, DMSO-*d*_6_) of 25″-Dehydroxydactylfungin (**3**), Figure S19: HMBC NMR spectrum (700 MHz, DMSO-*d*_6_) of 25″-Dehydroxydactylfungin (**3**), Figure S20: HPLC-UV/Vis chromatogram (210 nm) of the crude extract from the rice culture of *Amesia hispanica* sp. nov., Figure S21: Examples of fungal natural products featuring α-pyrone and glycosilated moieties [41,42,43,44,45,46], Table S1: Alignment used in the phylogenetic study.
