# Supplementary material for: Amesia hispanica sp. nov., Producer of the Antifungal Class of Antibiotics Dactylfungins
Source: J Fungi (Basel). 2023 Apr 12;9(4):463. doi: 10.3390/jof9040463 (PMC10141101; doi:10.3390/jof9040463)
Supplement: Supplementary file 1 [file jof-09-00463-s001.zip › jof-2278411-supplementary.pdf]

Supporting Material for

Amesia hispanica sp. nov., producer of the antifungal class of antibiotics dactylfungins

Esteban Charria-Girón[1,2], Alberto Miguel Stchigel[3], Adéla Čmoková[4], Miroslav Kolařík[4], Frank Surup[1,2] and Yasmina Marin-Felix[1,2*]

[1] Department Microbial Drugs, Helmholtz Centre for Infection Research (HZI), German Centre for Infection Research (DZIF), Partner Site Hannover-Braunschweig, Inhoffenstrasse 7, 38124 Braunschweig, Germany

[2] Institute of Microbiology, Technische Universität Braunschweig, Spielmannstraße 7, 38106 Braunschweig, Germany

[3] Mycology Unit, Medical School, Universitat Rovira i Virgili, C/Sant Llorenç 21, 43201 Tarragona, Spain

[4] Institute of Microbiology, Czech Academy of Sciences, Vídeňská 1083, 14220 Prague, Czech Republic

* Correspondence: yasmina.marinfelix@helmholtz-hzi.de

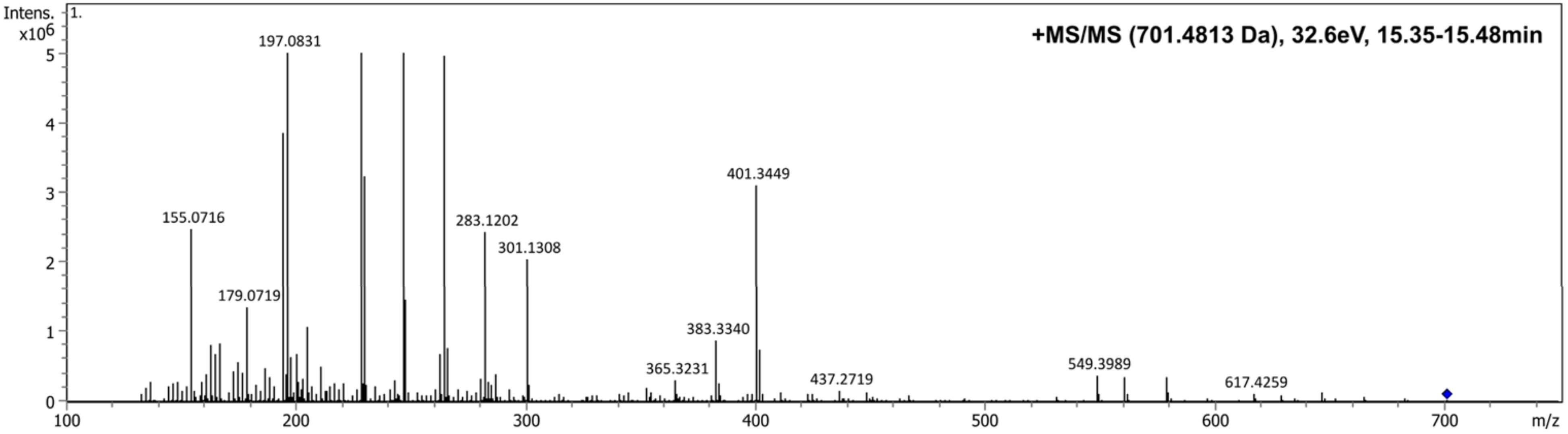

Figure S1. MS/MS spectrum of dactylfungin (**1**),
O=C1C([C@@H]2O[C@H](CO)C[C@H](O)[C@H]2O)=C(O)C=C(C(C)(C)C(O)/C=C/C(C)=C/C(C)CC(C)CC(C)C/C(C)=C/C=C/C(C)=C/C(C)CC)O1.

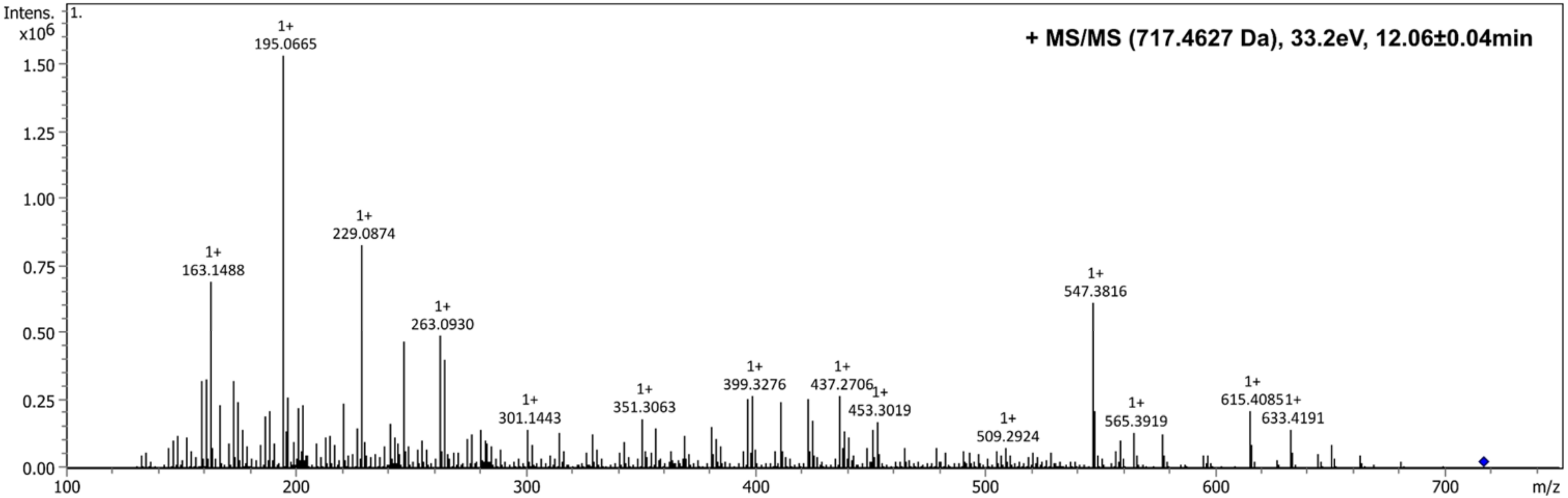

Figure S2. MS/MS spectrum of 21′′-Hydroxy-dactylfungin A (**2**),
O=C1C([C@@H]2O[C@H](CO)C[C@H](O)[C@H]2O)=C(O)C=C(C(C)(C)C(O)/C=C/C(C)=C/C(CO)CC(C)CC(C)C/C(C)=C/C=C/C(C)=C/C(C)CCO)O1.

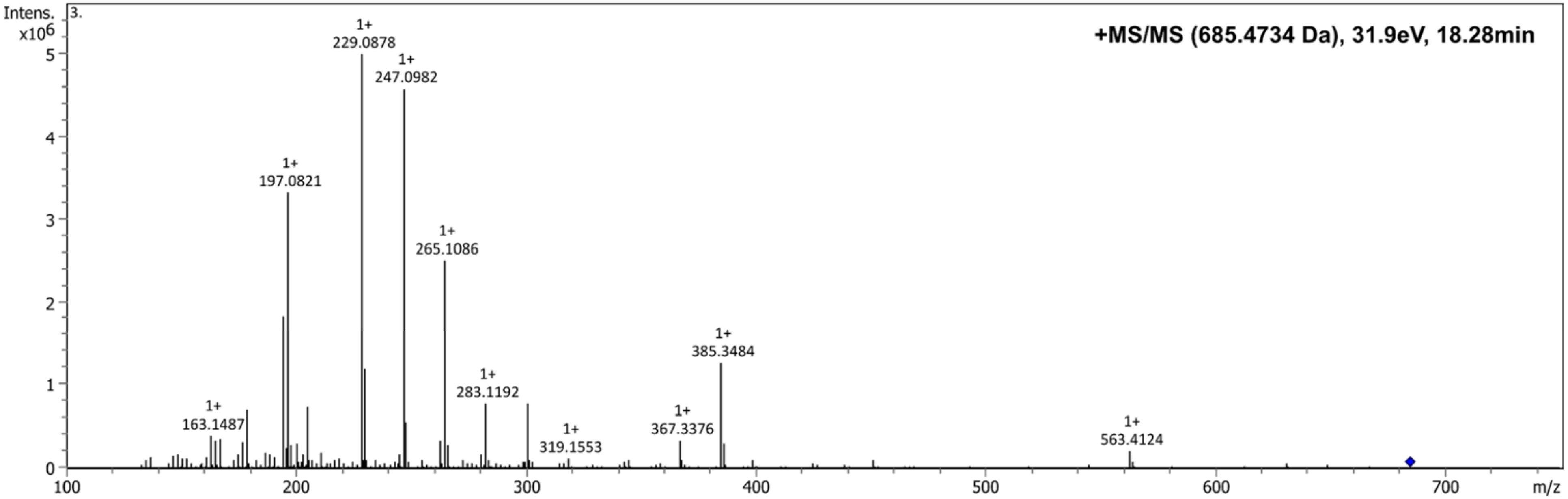

Figure S3. MS/MS spectrum of 25′′-Dehydroxy-dactylfungin A (**3**), O=C1C([C@@H]2O[C@H](CO)C[C@H](O)[C@H]2O)=C(O)C=C(C(C)(C)C(O)/C=C/C(C)=C/C(C)CC(C)CC(C)C/C(C)=C/C=C/C(C)=C/C(C)CC)O1.

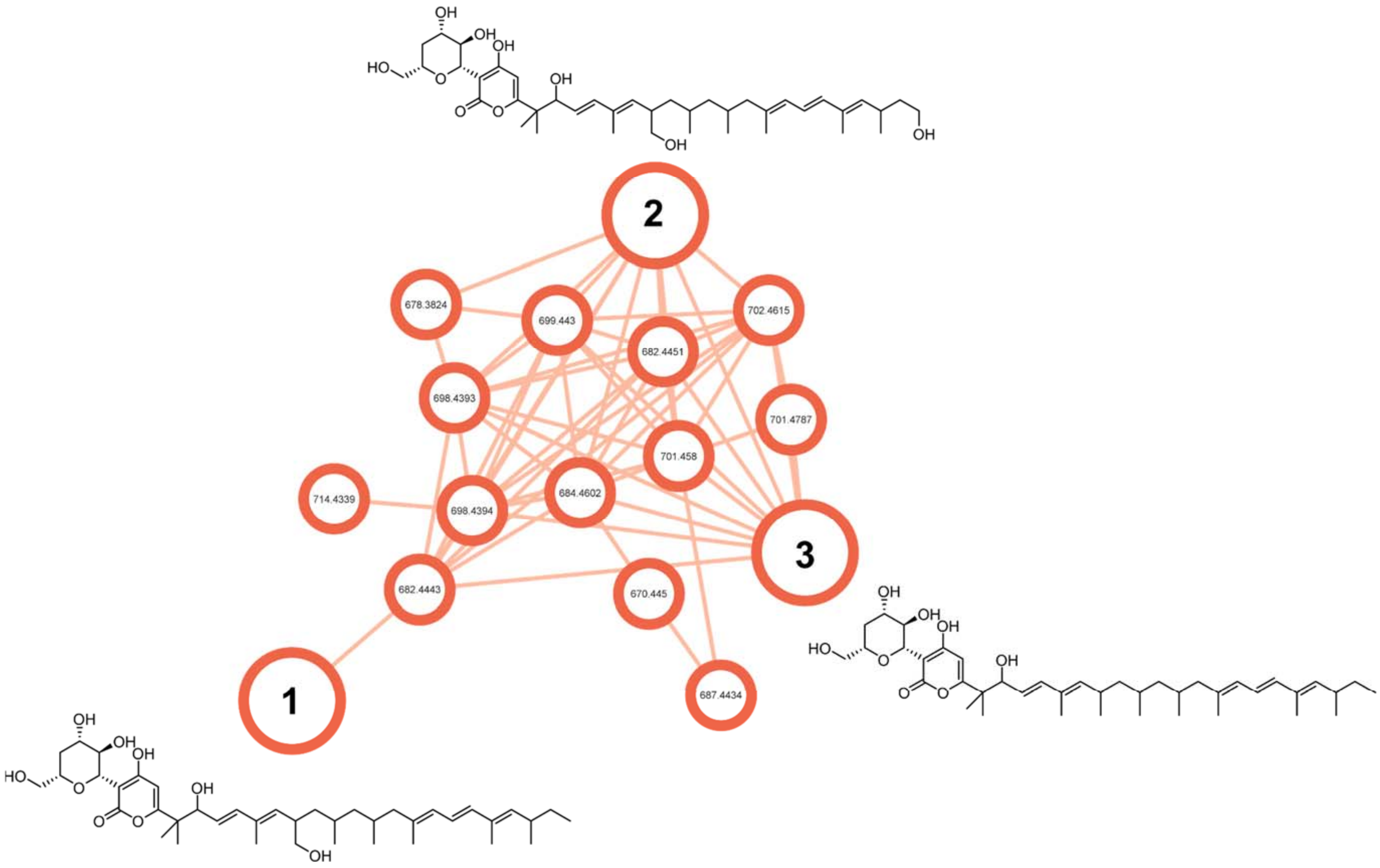

Figure S4. Dactylfungin molecular family created from the solid rice medium extract of the culture of *Amesia hispanica* sp. nov.

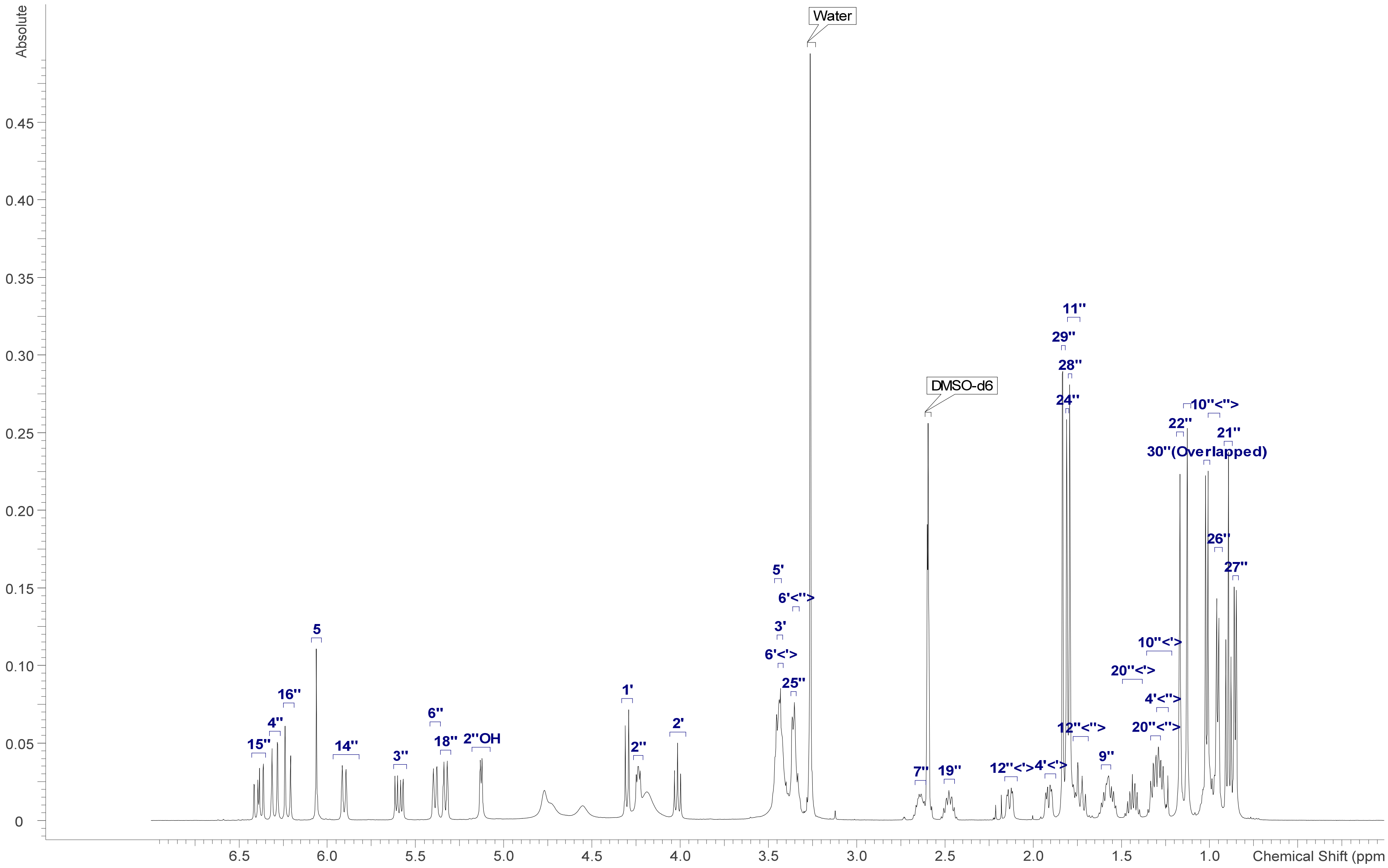

Figure S5. ^{1}H NMR spectrum (500 MHz, DMSO-d_6) of dactylfungin (**1**).

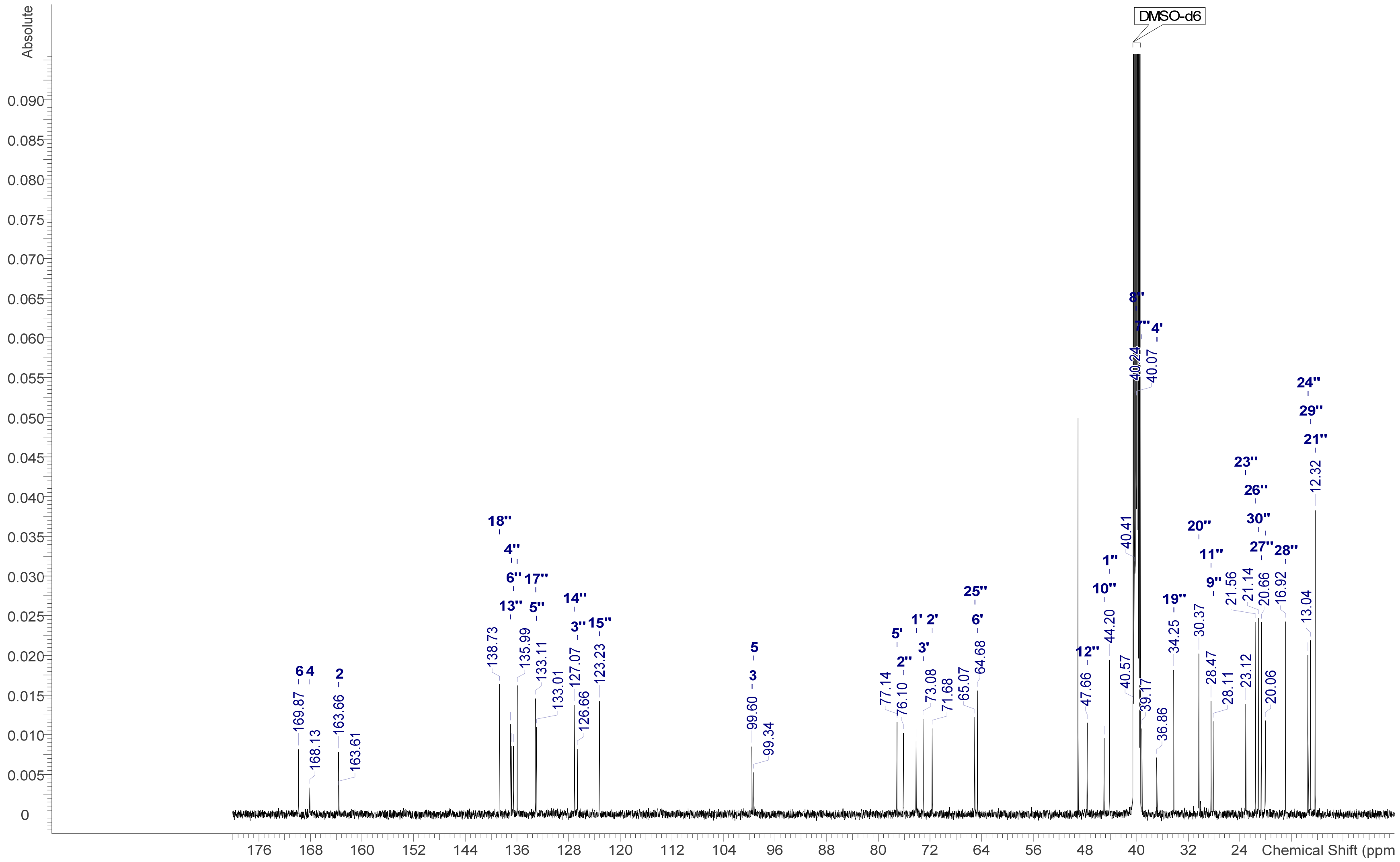

Figure S6. ^{13}C NMR spectrum (125 MHz, DMSO-d_6) of dactylfungin (**1**).

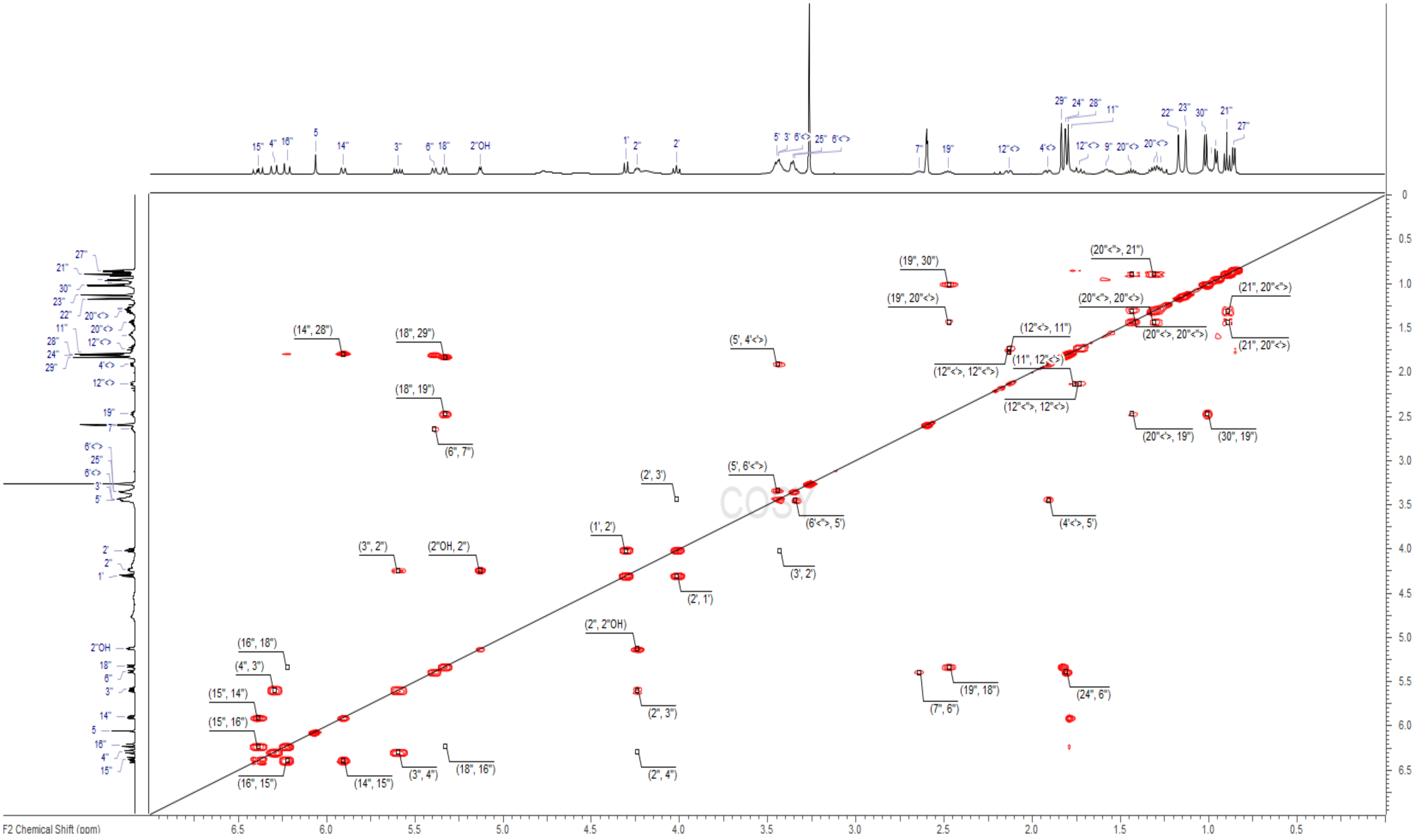

Figure S7. COSY NMR spectrum (500 MHz, DMSO-d_6) of dactylfungin (**1**).

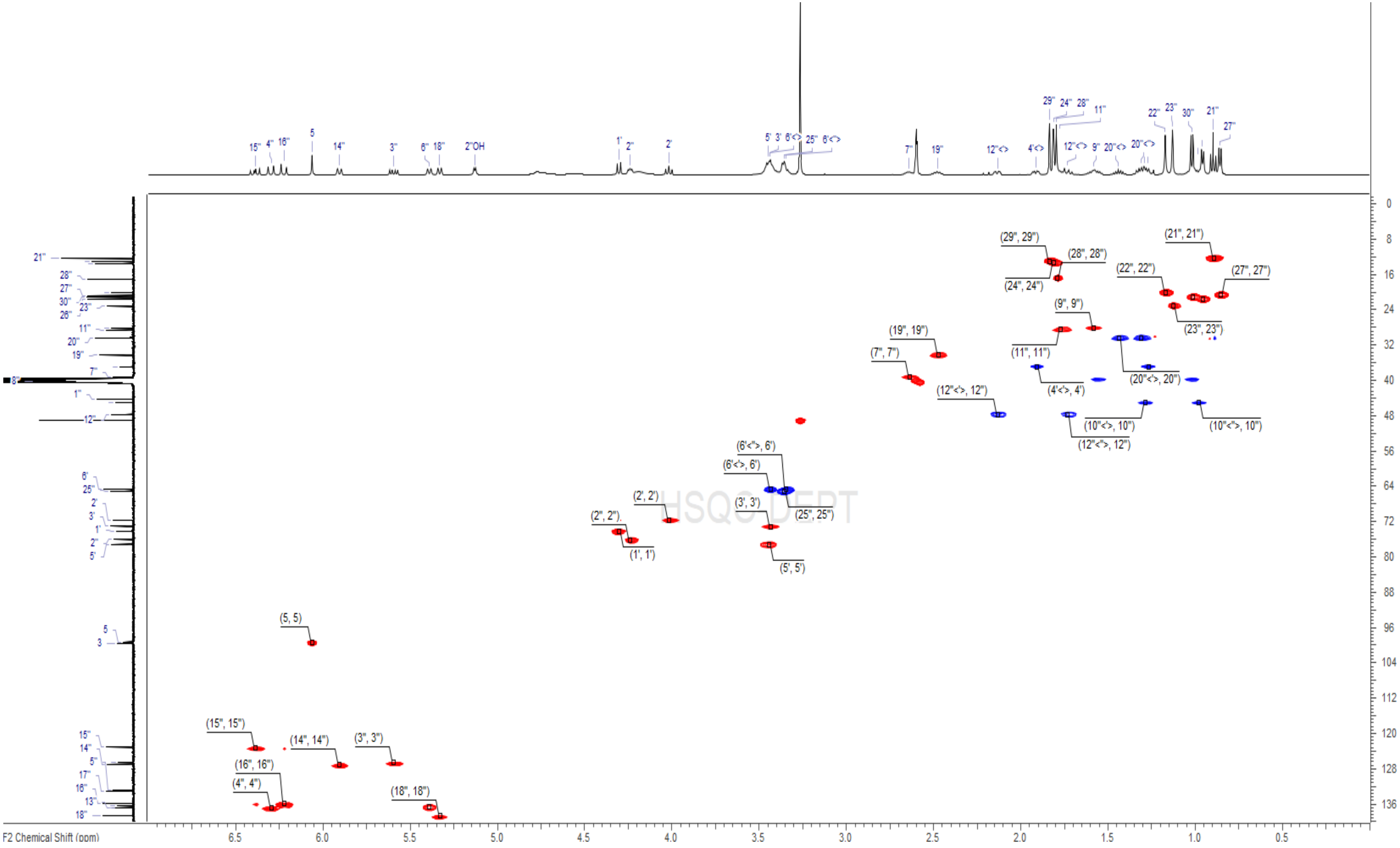

Figure S8. HSQC NMR spectrum (500 MHz, DMSO-d_6) of dactylfungin (**1**).

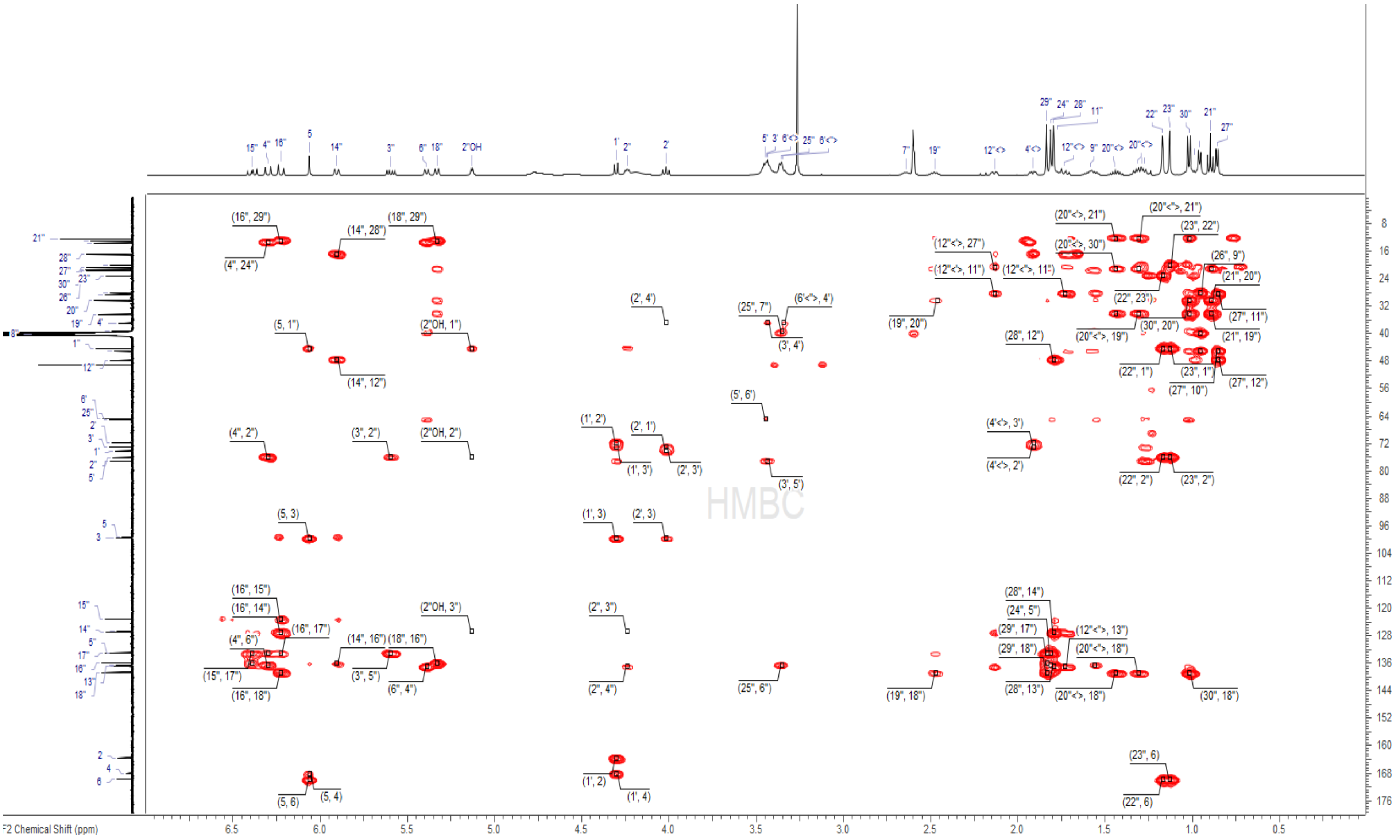

Figure S9. HMBC NMR spectrum (500 MHz, DMSO-d_6) of dactylfungin (**1**).

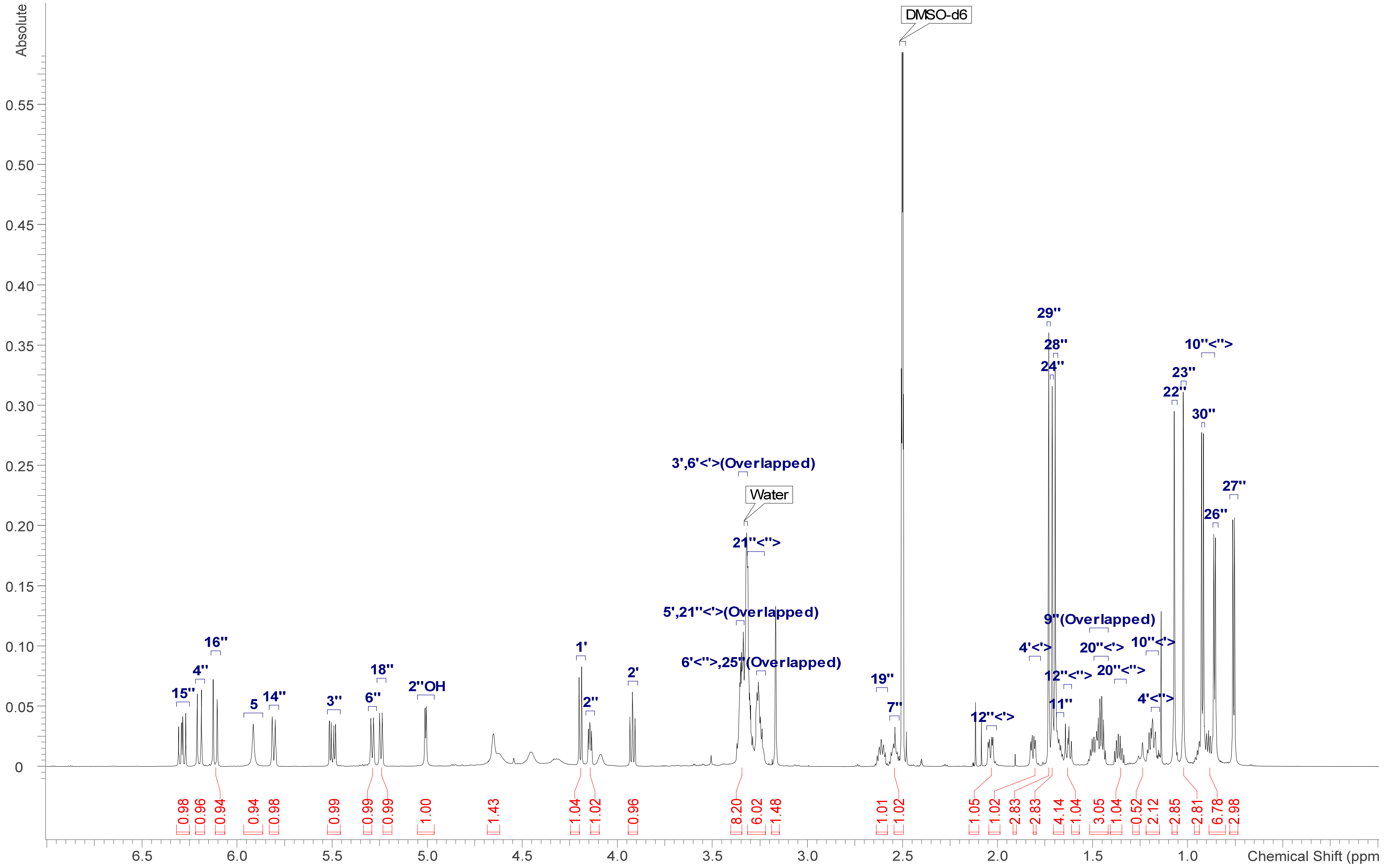

Figure S10. ^{1}H NMR spectrum (700 MHz, DMSO-*d*$_6$) of 21′′-Hydroxydactylfungin (**2**).

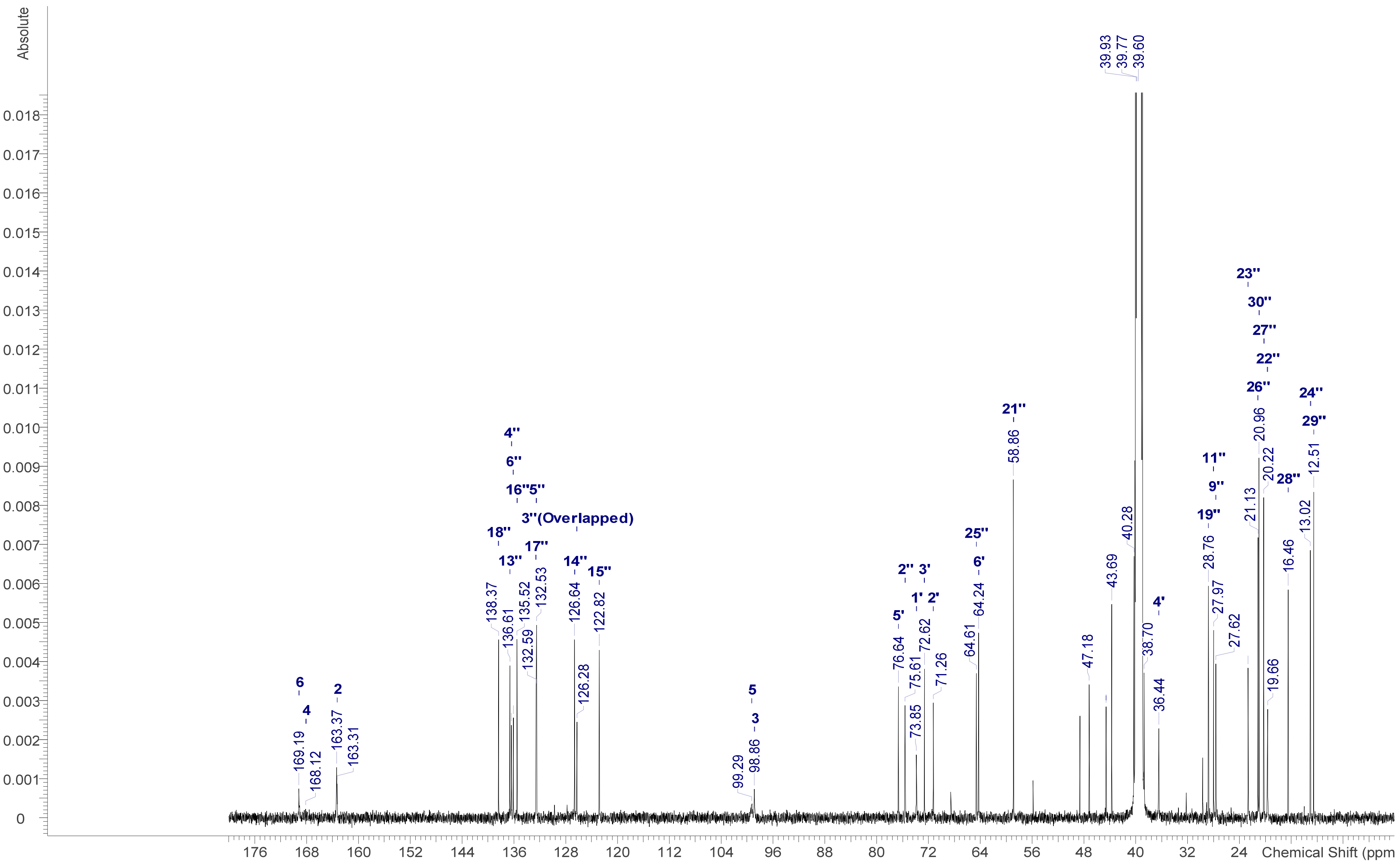

Figure S11. ^{13}C NMR spectrum (175 MHz, DMSO-d_6) of 21′′-Hydroxydactylfungin (**2**).

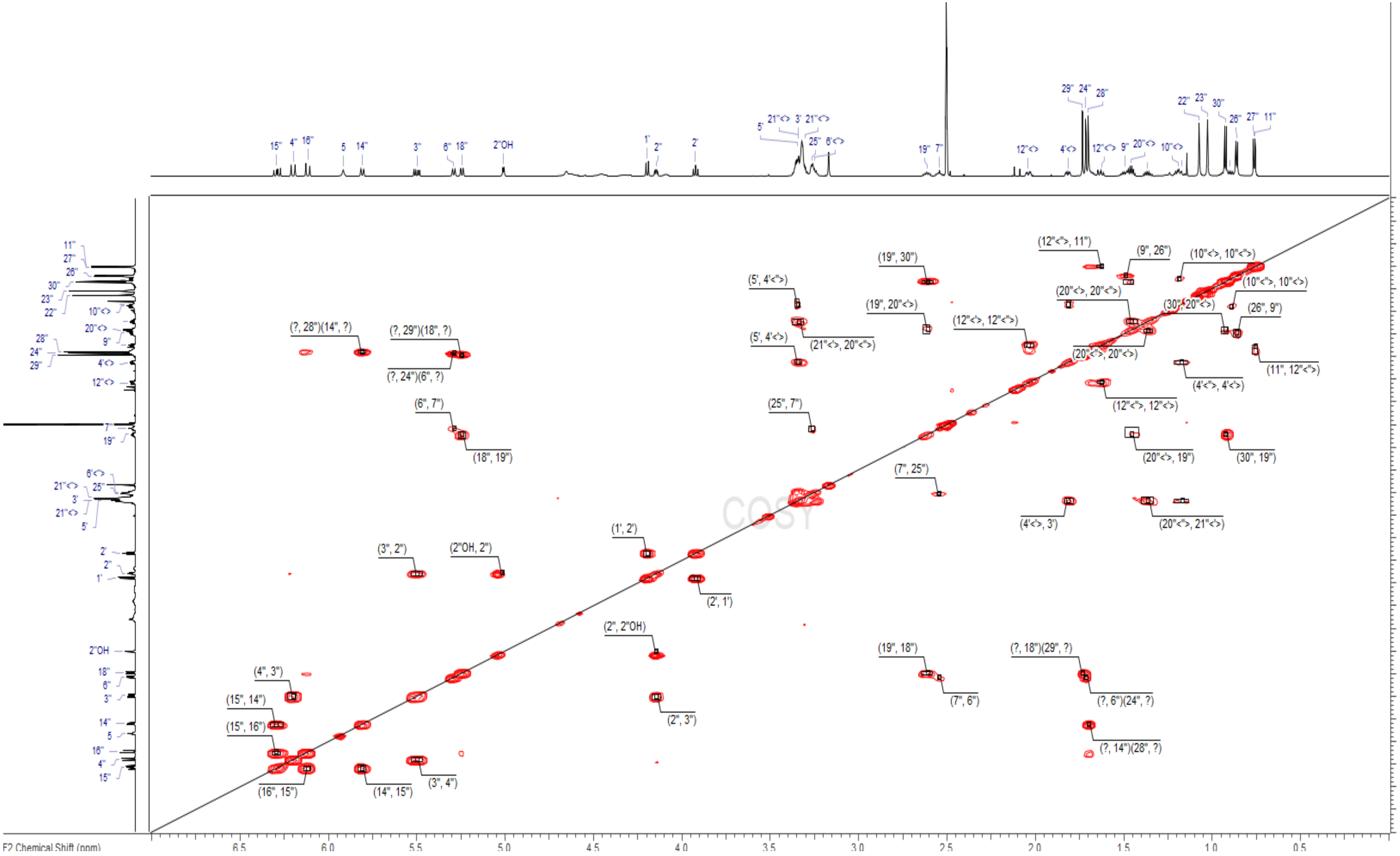

Figure S12. COSY NMR spectrum (700 MHz, DMSO-*d*$_6$) of 21′′-Hydroxydactylfungin (**2**).

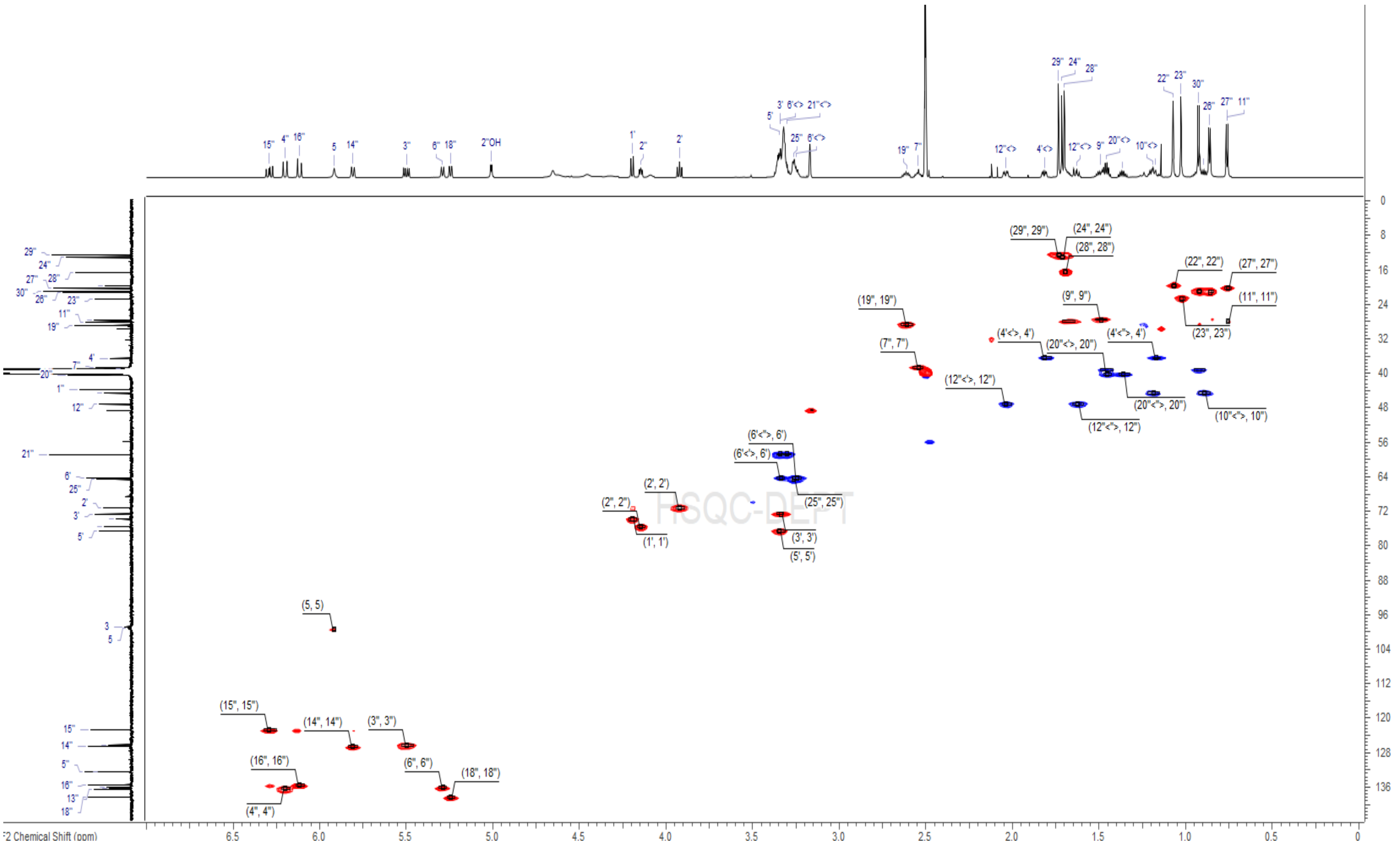

Figure S13. HSQC NMR spectrum (700 MHz, DMSO-d_6) of 21′′-Hydroxydactylfungin (**2**).

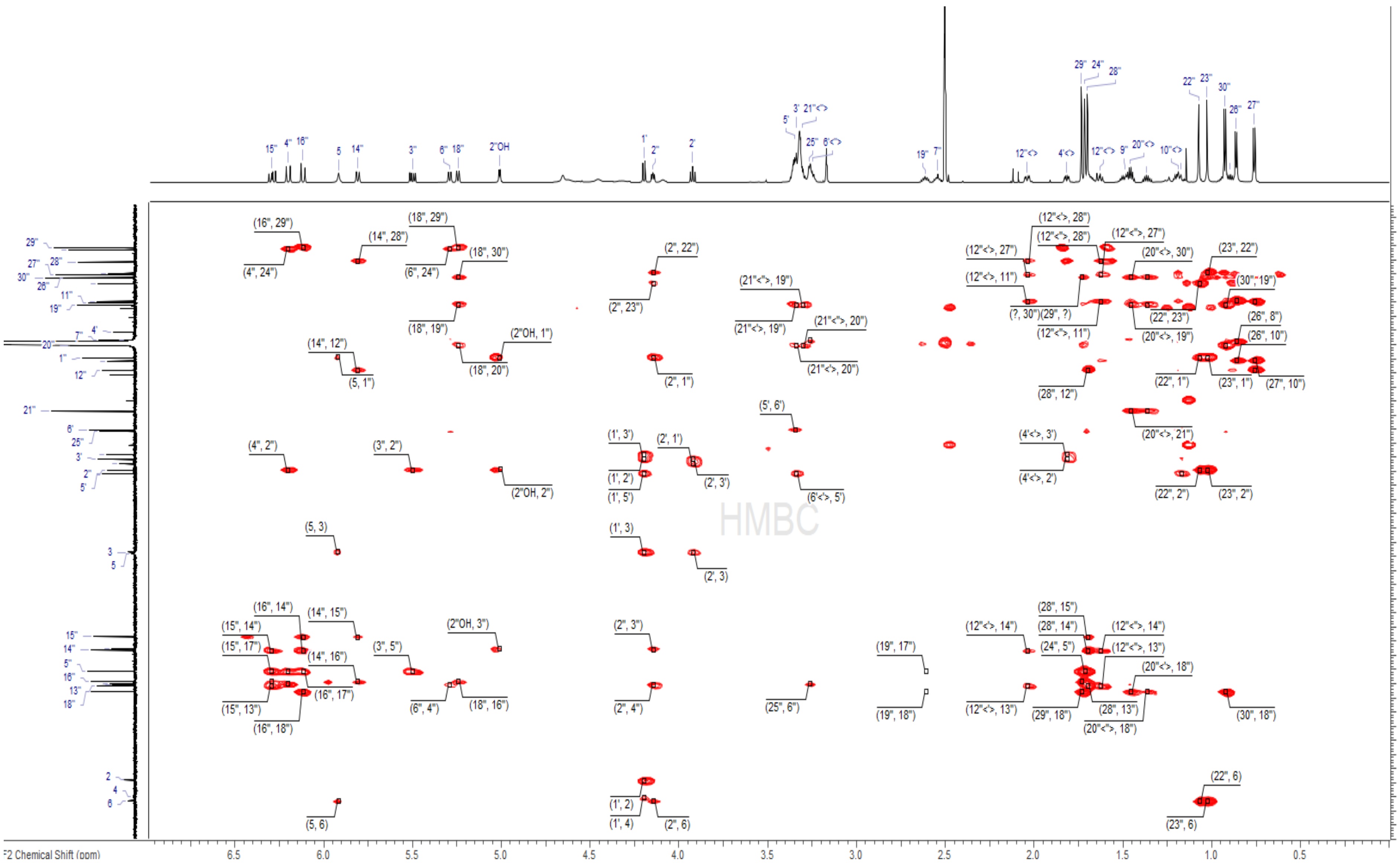

Figure S14. HMBC NMR spectrum (700 MHz, DMSO-d_6) of 21′′-Hydroxydactylfungin (**2**).

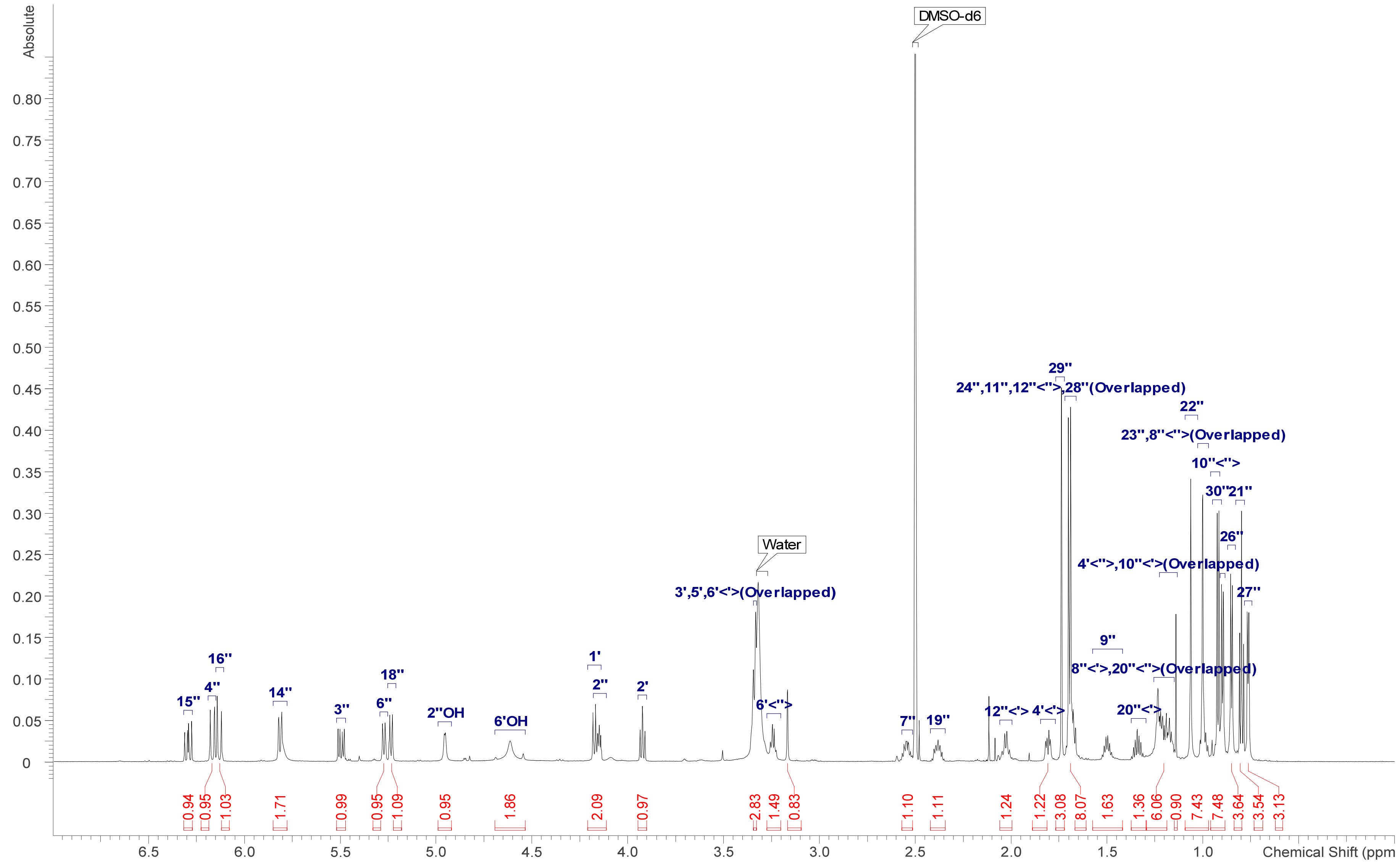

Figure S15. ^{1}H NMR spectrum (700 MHz, DMSO-d_6) of 25′′-dehydroxydactylfungin (**3**).

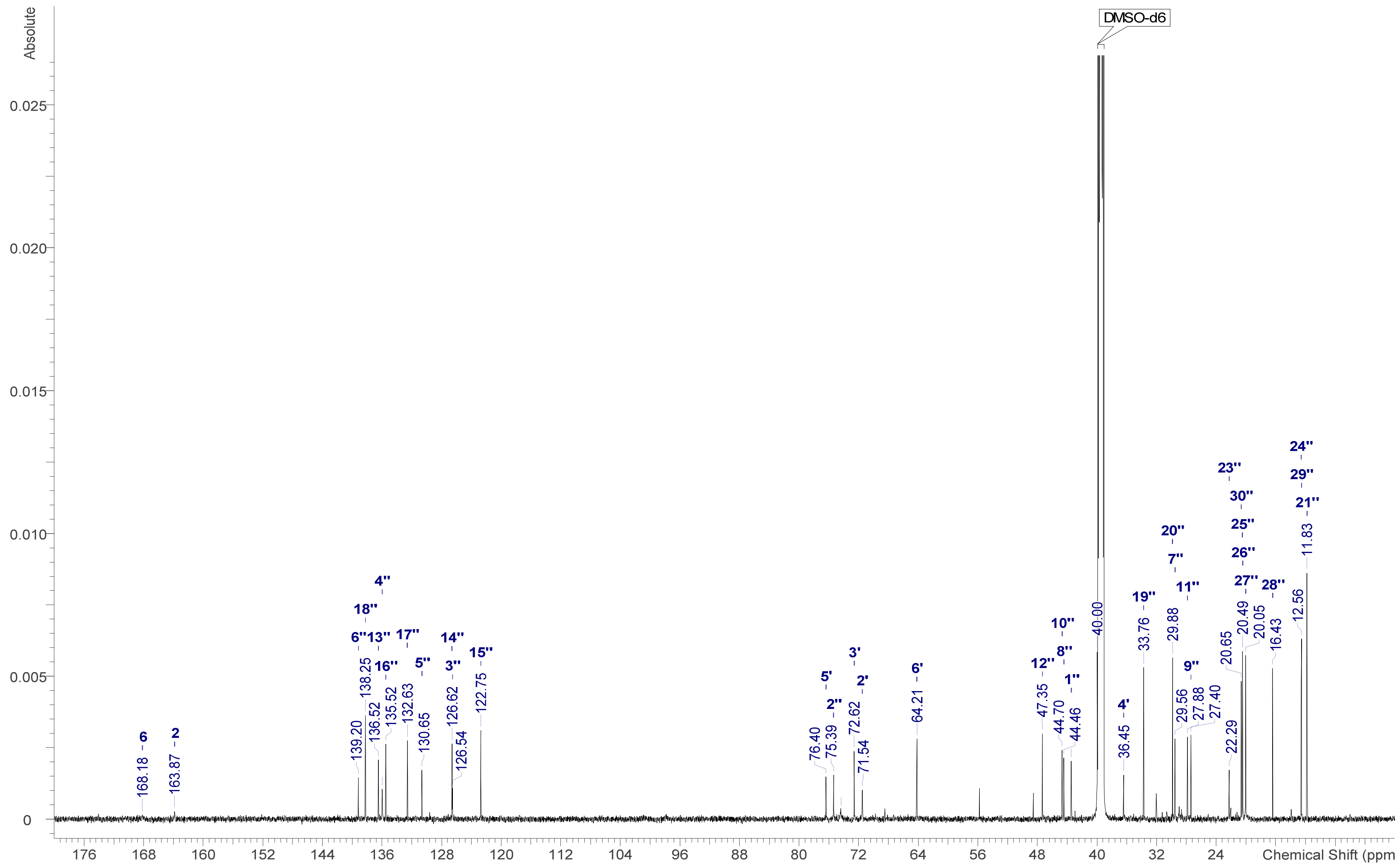

Figure S16. ^{13}C NMR spectrum (175 MHz, DMSO-d_6) of 25′′-Dehydroxydactylfungin (**3**).

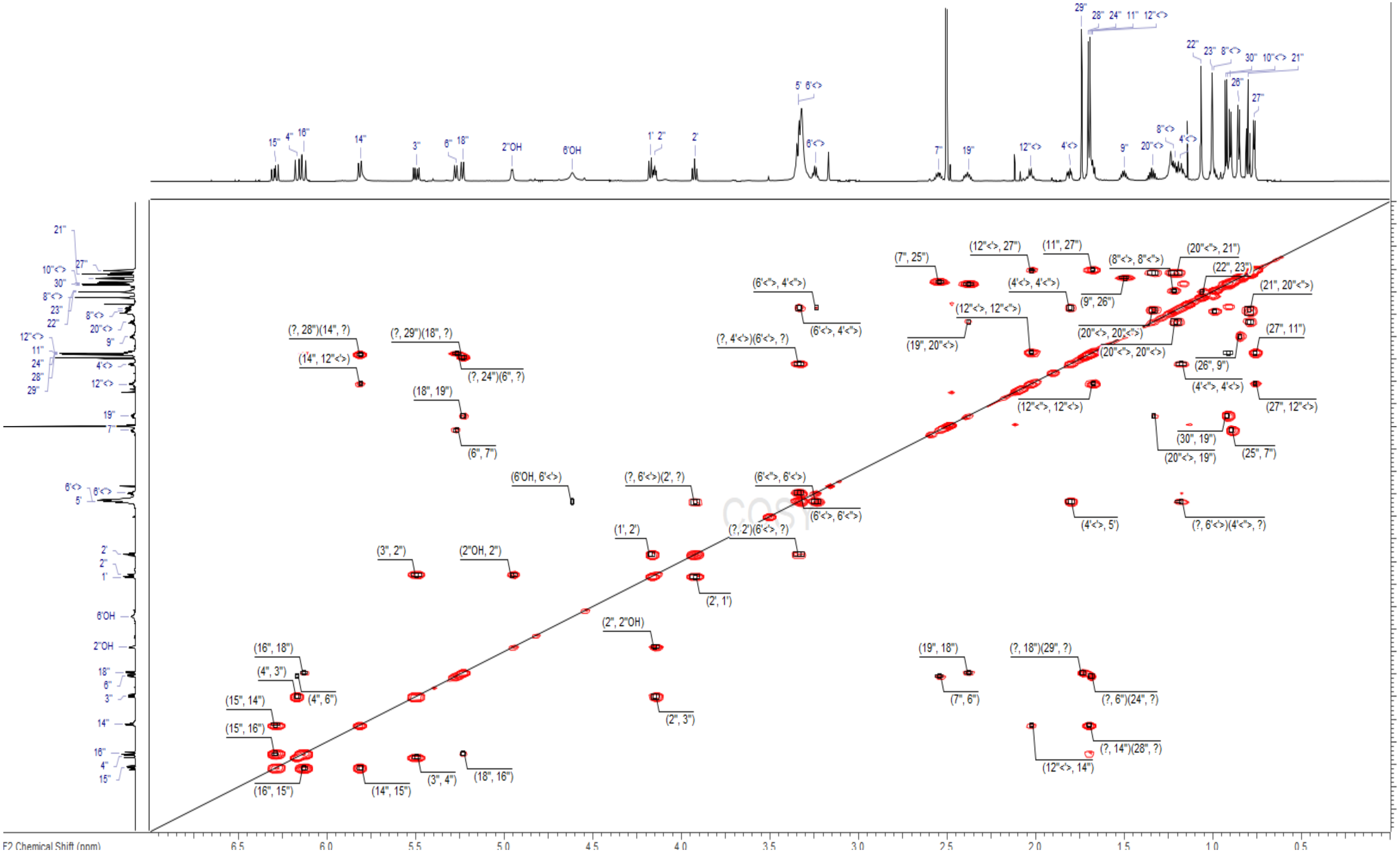

Figure S17. COSY NMR spectrum (700 MHz, DMSO-d_6) of 25′′-Dehydroxydactylfungin (**3**).

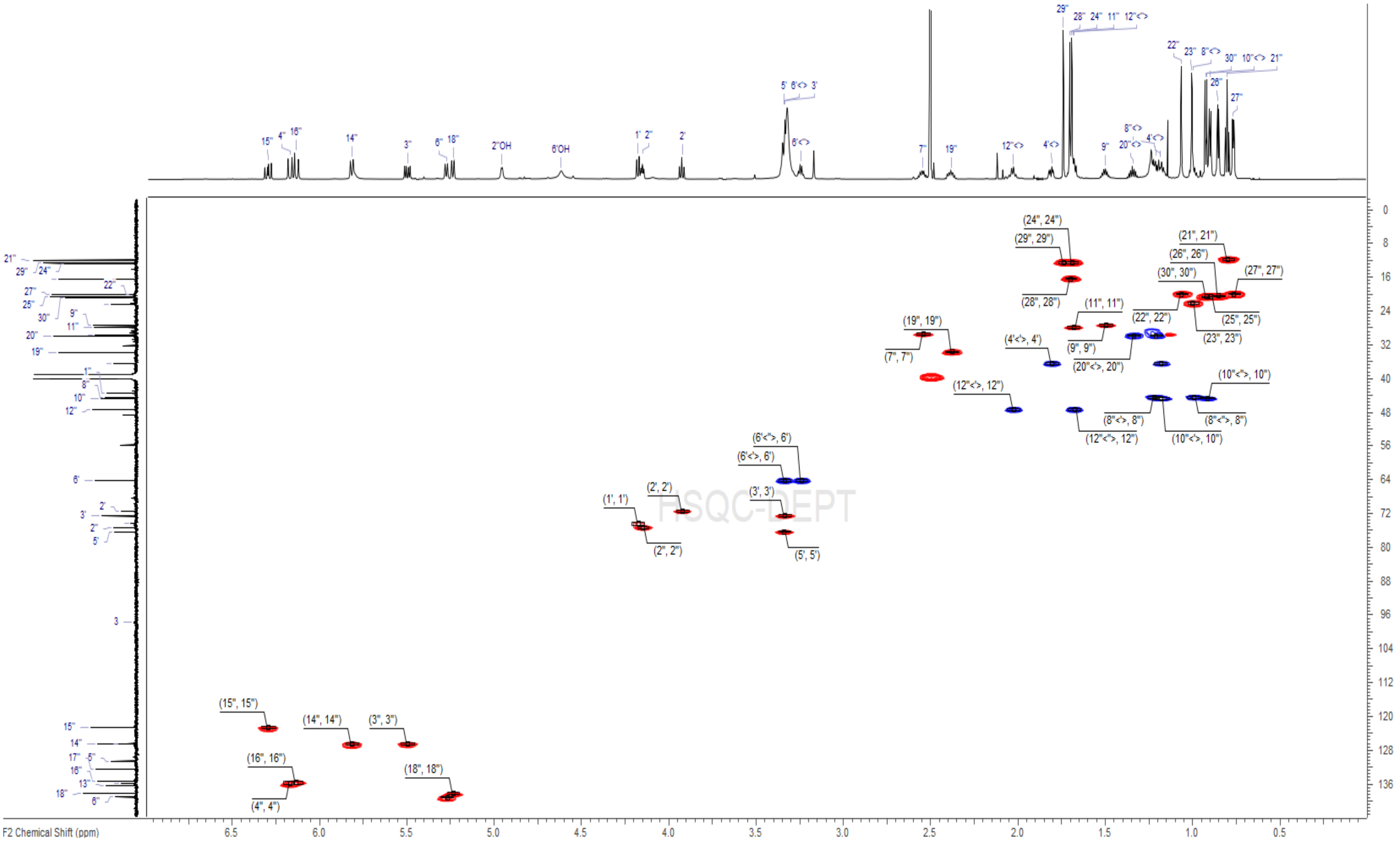

Figure S18. HSQC NMR spectrum (700 MHz, DMSO-d_6) of 25′′-Dehydroxydactylfungin (**3**).

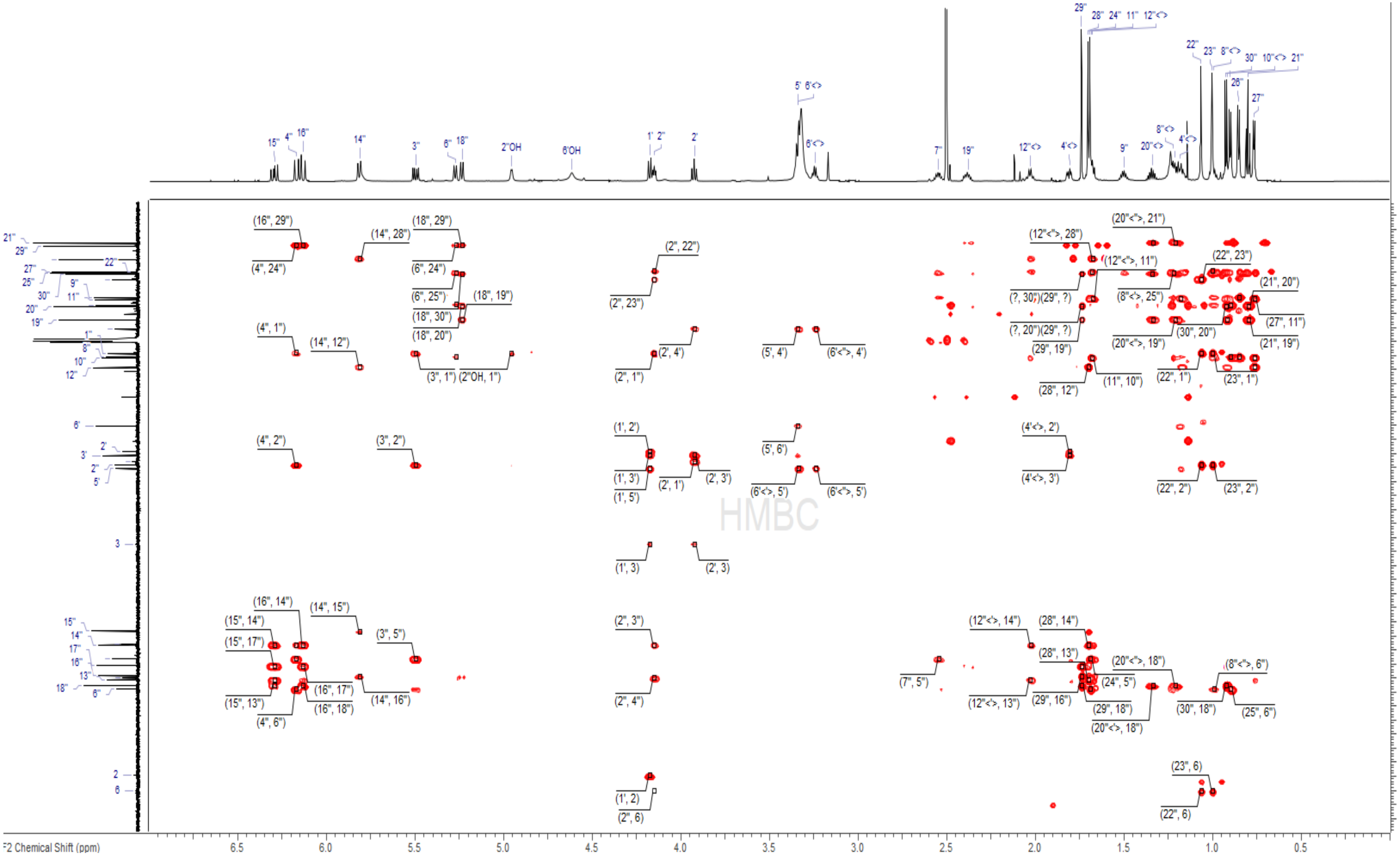

Figure S19. HMBC NMR spectrum (700 MHz, DMSO-d_6) of 25′′-Dehydroxydactylfungin (**3**).

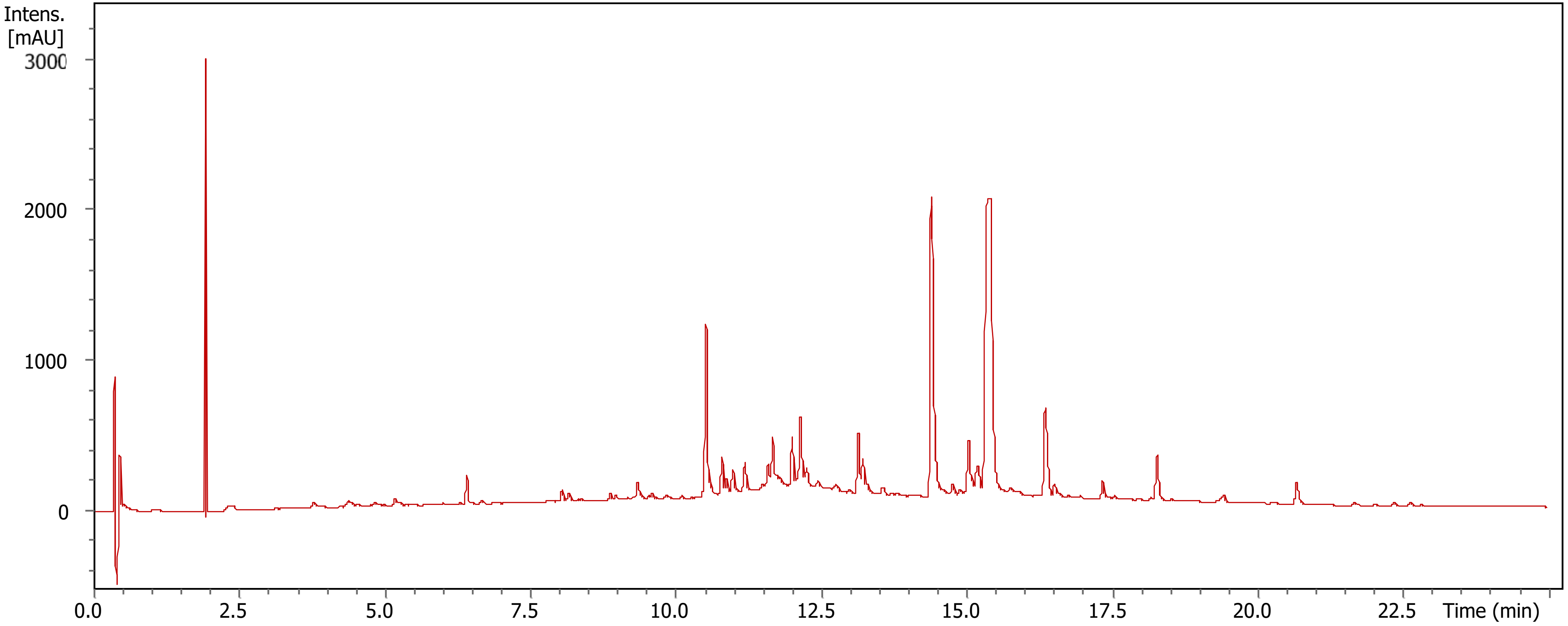

Figure S20. HPLC-UV/Vis chromatogram (210 nm) of the crude extract from the rice culture of *Amesia hispanica* sp. nov.

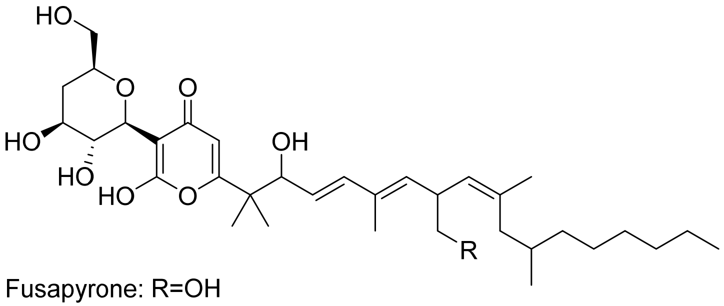

Fusapyrone: R=OH
Desoxyfusapyrone: R=H

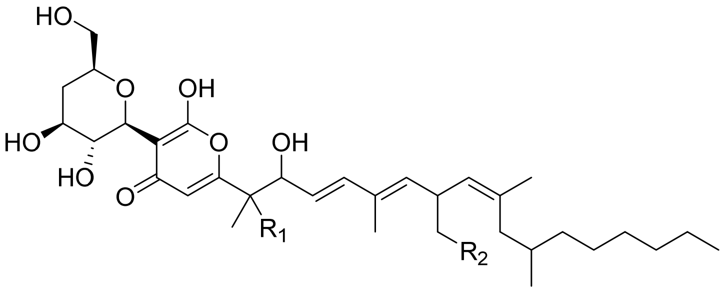

Deoxyneofusapyrone: R_1=CH_3, R_2=H
7-desmethyldeoxyneofusapyrone: R_1=H, R_2=H
Neofusapyrone: R_1=CH_3, R_2=OH

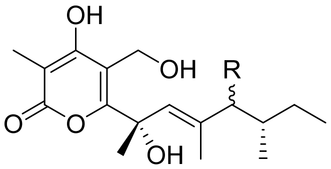

Neovasipyrone A: R=α-OH,β-H
Neovasipyrone b: R=β-OH,α-H

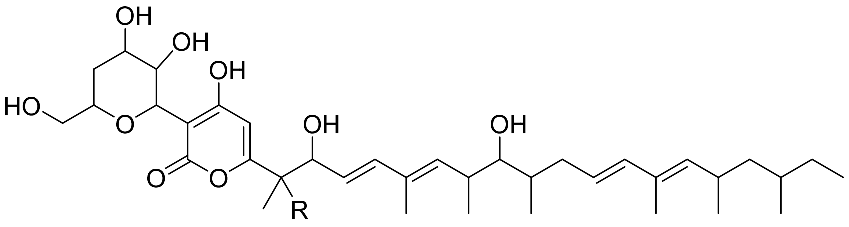

YM-202204: R=H
S39163/F-1: R=CH_3

Figure S21. Examples of fungal natural products featuring α-pyrone and glycosilated moieties [41–46].

Table S1. Alignment used in the phylogenetic study.

>Amesia_hispanica_FMR12004

ATTACAGAGTTGC-AAAACTCCCTAAACCATTGTGAACCTTACCTTT-AACCGTTGCTTCGGCGGGCGGCGCTCT----------------------------
GCCGCCCCTCGGCCCTGC----------------------CTGGGCGCCCGCCGGAGGATACCC-AACTCTTGATTCTTG--
ATGGCCTCTCTGAGTCTTCTGTACTGAA-
TAAGTCAAAACTTTCAACAACGGATCTCTTGGTTCTGGCATCGATGAAGAACGCAGCGAAATGCGATAAGTAATGTGAATTGCAGAATTC
AGTGAATCATCGAATCTTTGAACGCACATTGCGCCCGCCAGTATTCTGGCGGGCATGCCTGTTCGAGCGTCATTTCAACCATCAAGCCCAC
-GGCTTGTGTTGGGGACCTGCG----------GCTGCCCGCAGGCCCTGAAAACCAGTGGCGGGCTCGCT-GTCACACCGAGCGTAGTAG--
CATCATTCTCGCTCAGGGCG-TGCTGCGGGTT--CCGGCCGTTAAA-
CGACCTTCATAACCCAAGGTTGACCTCGGATCAGGTAGGAAGACCCGCTGAACTTAA???????????????????AAAGAAACCAACAGGGATT
GCCC-TAGTAACGGCG-AGTGAAGCGGCAACAGCTCAAATTTGAAATCTGGCTTCGGCCCGAGTTGTAATTTGCAG-
AGGAAGCTTTAGGCGCGGCACCTTCTGAGTCCCCTGGAACGGGGCGCCATAGAGGGTGAGAGCCCCGTATAGTTGGATGCCTAGCCTGT
GTAAAGCTCC-
TTCGACGAGTCGAGTAGTTTGGGAATGCTGCTCAAAATGGGAGGTAAATTTCTTCTAAAGCTAAATACCGGCCAGAGACCGATAGCGCA
CAAGTAGAGTGATCGAAAGATGAAAAGCACTTTGAAAAGAGGGTTAAATAGCACGTGAAATTGTTGAAAGGGAAGCGCTTGTGACCAG
ACTTGCGCCGGGCTGATCATCCGGTGTTCTCACCGGTGCACTCTGCCCGGCTCAGGCCAGCATCGGTTCTCGCGGGGGGATAAAGGTTCC
GGGAATGTAGCTCCTCCGGGAGTGTTATAGCCCGGGGCGTAATGCCCTCGCGGGGACCGAGGTTCGCGC-
TCTGCAAGGATGCTGGCGTAATGGTCATCAGCGACCCGTCTTGAAACACGGACCAAGGAGTCAAGGTTTTGCGCGAGTGTTTGGGTGTAA
AACCCGCACGCGTAATGAAAGTGAACGTAGGTGAGAGCTTCGGCGCATCATCGACCGATCCTGATGTTTTCGGATGGATTTGAGTAAGAG
CGTTAAGCCTTGGACCCGAAAGATGGTGAACTATGCTTGGATAGGGTGAAGCCAGAGGAAACTCTGGTGGAGGCTCGCAGCGG-
TTCTGACGTGCAAATCGATCGTCAAATCTGAGCATCTGGTCACATTGGTGCAAGGTCTGCGGAGGAAGAACGTCATTTCGTTCGAGGTGT
CGTTGGTTCGCGACATCCGCGACCGCGAGTTCAAGATCTTTTCCGATGCTGGGCGCGTCATGAGGCCGCTGTTTACCGTCGAGCAGGAGC
ATGGTTCAGAGACCGGCGCCGAAATGGGGCAACTCATCCTCAACAAGGAGCATATTACACGGTTAGAAGCGGACAAGGAGCTGGGCAA
ATACCATCCCGATTATTGGGGCTGGCAGGGCCTGCTGAAGTCAGGTGCCATCGAGTACCTCGATGCCGAGGAGGAGGAGACGGCCATGA
TCTGCATGACGCCCGAGGATCTCGACAAGTTCCGGTACAGAAAGATGGGGTTCATCGTCGAAGACAACTCGGGTCAAGGTAACAACAGG
ATCAAGACGAAGCCGAACCCCGCCACCCACATGTACACGCACTGCGAGATCCATCCCAGCATGCTGCTCGGCATCTGCGCCAGCATCAT
CCCCTTCCCCGACCACAACCAG???????????AGCTTT-CTTTT-GGCCCCCCTGAT---CTATCC--CACACATTGGGA-TAACCTCGTT---

CCACCCGCT--ACCCGA----CCGACC----GAGAACGATGACGGCGCGGCCATAA--TGGACA-----GGACACGCGTGCTGACAT--
GGCTTCTTTACTACAGGTTCACCTCCAGACCGGCCAGTGCGTAAGTTTGATCGATTC--GAGC----CCGACGACC-GATACTGTG----
TGGTGGTGC------GGGTGAAGACTGACTACAAGCT-CTCCAGGGTAACCAAATTGGTGCTGCTTTCTGGTGTG-TTCG--
ACGCCAACATTAACCGC-----TTGCGTGTCAA-
AGGCAATCAAGACTGACTTCCATTACAGGCAGACCATCTCTGGTGAGCACGGCCTCGACAGCAATGGCGTGTACGTGACTGTCGCCGAC
CCAAA-TCCCCATAATCTCCC---
TTGCTCACCGCTACAAATAGGTACAACGGCACCTCGGAGCTCCAGCTCGAGCGCATGAATGTCTACTTCAACGAGGTGAGTCAGGCCAGT
ACAC----CATCAATTTTCT----TGAAGGCTGCGGGTCGCTGACAACATA----
ATCTTCGCAGGCTTCCGGCAACAAATATGTCCCTCGTGCCGTCCTCGTCGACTTGGAGCCCGGCACCATGGATGCTGTTCGCGCTGGTCCC
TTCGGCCAGCTCTTCCGCCCTGATAACTT

>Amesia_raii_CBS_106.83

??
??
??
??
??
??
??
??
??
??TGGTCACGCT
GGTGCAGGGCCTGCGGAGGAAGAACGTCATTTCCTTCGAGGTTTCGCTGGTTCGCGACATCCGCGACCGCGAGTTCAAGATTTTCTCGGA
TGCTGGACGTGTCATGAGGCCGCTGTTTACTGTCGAGCAGGAGCACGGTTCAGACTCGGGCGCCGAAATGGGCCAGCTCATCCTCAACAA
GGAGCACATTACGCGGTTAGAAACGGACAAGGAGCTGGGCAAATACCACCCCGACTACTGGGGCTGGCAGGGCCTCTTGAAGTCAGGT
GCGATCGAGTACCTCGACGCCGAGGAGGAGGAGACGGCCATGATTTGCATGACGCCCGAAGACCTCGACAAGTTCCGGTACAGAAAAA
TGGGGTTCATCGTCGAAGACAACTCAGGTCAAGGTAACAACAGGATCAAGACGAAGCCTAACCCCGCCACCCACACGTACACGCACTG
CGAGATTCACCCCAGCATGCTGCTCGGCATCTGCGCCAGCATCATTCCCTTCCCGGACCACAACCAG??????????????????????????????????????

???
???
???
???
???

>Amesia_raii_CBS_107.83

???
???
???
???
???
???
???
???
???
??TGGTCACGCT
GGTGCAGGGCCTGCGGAGGAAGAACGTCATTTCCTTCGAGGTTTCGCTGGTTCGCGACATCCGCGACCGCGAGTTCAAGATTTTCTCGGA
TGCTGGACGTGTCATGAGGCCGCTGTTTACTGTCGAGCAGGAGCACGGTTCAGACTCGGGCGCCGAAATGGGCCAGCTCATCCTCAACAA
GGAGCACATTACGCGGTTAGAAACGGACAAGGAGCTGGGCAAATACCACCCCGACTACTGGGGCTGGCAGGGCCTCTTGAAGTCAGGT
GCGATCGAGTACCTCGACGCCGAGGAGGAGGAGACGGCCATGATTTGCATGACGCCCGAAGACCTCGACAAGTTCCGGTACAGAAAAA
TGGGGTTCATCGTCGAAGACAACTCAGGTCAAGGTAACAACAGGATCAAGACGAAGCCTAACCCCGCCACCCACACGTACACGCACTG
CGAGATTCACCCCAGCATGCTGCTCGGCATCTGCGCCAGCATCATTCCCTTCCCGGACCACAACCAG???
???
???
???
???
???

>Amesia_IRAN_3489C

ATTACAGAGTTGC-AAAACTCCCTAAACCATTGTGAACGTTACC-TTCAACCGTTGCTTCGGCGGGCGGCGCTCT----------------------------
GCCGCCCCTCGGCCCCGC----------------------CCGGGCGCCCGCCGGAGGATACCC-AACTCTTGATACTTT--
ATGGCCTCTCTGAGTCTTCTGTACTGAA-
TAAGTCAAAACTTTCAACAACGGATCTCTTGGTTCTGGCATCGATGAAGAACGCAGCGAAATGCGATAAGTAATGTGAATTGCAGAATTC
AGTGAATCATCGAATCTTTGAACGCACATTGCGCCCGCCAGTATTCTGGCGGGCATGCCTGTTCGAGCGTCATTTCAACCATCAAGCCCAC
-GGCTTGTGTTGGGGACCTGCG----------GCTGCCCGCAGGCCCTGAAAACCAGTGGCGGGCTCGCT-GTCACACCGAGCGTAGTAG--
CATCATTCTCGCTCAGGGCG-TGCTGCGGGTT--CCGGCCGTTAAA-
CGACCTTCATAACCCAAGGTTGACCTCG???
??
??
??
??
??
??TGGTCACGTTGGTACAAGGTCTGCGGAGGAAGAACGTCATTTCGTTCGAGGTGTCGTTG
GTTCGCGACATCCGCGACCGCGAGTTCAAGATCTTTTCCGATGCTGGGCGCGTCATGAGGCCGCTGTTTACCGTTGAGCAGGAGCACGGT
TCAGAGACTGGCGCCGAAATGGGGCAGCTCATTCTCAACAAGGAGCATATCACACGGTTAGAAGCGGACAAGGAGCTGGGCAAATACC
ATCCCGACTACTGGGGCTGGCAGGGCCTGCTGAAGTCGGGTGCCATCGAGTATCTCGATGCCGAGGAGGAGGAGACGGCCATGATCTGC
ATGACGCCCGAGGACCTGGACAAGTTCCGGTACAGAAAGATGGGATTCATCGTCGAAGACAACTCGGGTCAAGGTAACAATAGGATCA
AGACGAAGCCGAACCCCGCCACCCACATGTACACGCACTGCGAGATTCATCCCAGCATGTTGCTCGGCATCTGCGCCAGCATCATCCCCT
TCCCCGACCACAACCAG??
??
???????????????????????????????????AACCAAATCGGTGCTGCTTTCTGGTATG-TTTG--ATGCCAGTATCAAACGC-----TTACGTGTCAA-
CGGCAATCAAGACTGACTTCCATCGCAGGCAGACCATCTCAGGCGAGCACGGCCTCGACAGCAATGGCGTGTACGTGACTATCGCCGAC
CCAAA-TTCTCATAA--TCCC---
TCGCTCACCGCTACAAATAGGTACAACGGCACCTCGGAGCTCCAGCTCGAGCGCATGAATGTCTACTTCAACGAGGTGAGTCGGACCCGT
ACACG-TTTTTCAGTTATCT----CGAAGTTTGC-GGTTGCTGACAATATA----

TTCTTCGCAGGCTTCTGGCAACAAATATGTCCCTCGCGCCGTCCTCGTCGACTTGGAGCCCGGCACCA?????????????????????????????????????
???????????????

>Amesia_SCUA_Saf_B16_2

ATTACAGAGTTGC-AAAACTCCCTAAACCATTGTGAACGTTACC-TTCAACCGTTGCTTCGGCGGGCGGCGCTCT----------------------------
GCCGCCCCTCGGCCCCGC----------------------CCGGGCGCCCGCCGGAGGATACCC-AACTCTTGATACTTT--
ATGGCCTCTCTGAGTCTTCTGTACTGAA-
TAAGTCAAAACTTTCAACAACGGATCTCTTGGTTCTGGCATCGATGAAGAACGCAGCGAAATGCGATAAGTAATGTGAATTGCAGAATTC
AGTGAATCATCGAATCTTTGAACGCACATTGCGCCCGCCAGTATTCTGGCGGGCATGCCTGTTCGAGCGTCATTTCAACCATCAAGCCCAC
-GGCTTGTGTTGGGGACCTGCG----------GCTGCCCGCAGGCCCTGAAAACCAGTGGCGGGCTCGCT-GTCACACCGAGCGTAGTAG--
CATCATTCTCGCTCAGGGCG-TGCTGCGGGTT--CCGGCCGTTAAA-
CGACCTTCATAACCCAAGGTTGACCTCG???
???
???
???
???
???
??TGGTCACGTTGGTACAAGGTCTGCGGAGGAAGAACGTCATTTCGTTCGAGGTGTCGTTG
GTTCGCGACATCCGCGACCGCGAGTTCAAGATCTTTTCCGATGCTGGGCGCGTCATGAGGCCGCTGTTTACCGTTGAGCAGGAGCACGGT
TCAGAGACTGGCGCCGAAATGGGGCAGCTCATTCTCAACAAGGAGCATATCACACGGTTAGAAGCGGACAAGGAGCTGGGCAAATACC
ATCCCGACTACTGGGGCTGGCAGGGCCTGCTGAAGTCGGGTGCCATCGAGTATCTCGATGCCGAGGAGGAGGAGACGGCCATGATCTGC
ATGACGCCCGAGGACCTGGACAAGTTCCGGTACAGAAAGATGGGATTCATCGTCGAAGACAACTCGGGTCAAGGTAACAATAGGATCA
AGACGAAGCCGAACCCCGCCACCCACATGTACACGCACTGCGAGATTCATCCCAGCATGTTGCTCGGCATCTGCGCCAGCATCATCCCCT
TCCCCGACCACAACCAG???
???
????????????????????????????????????AACCAAATCGGTGCTGCTTTCTGGTATG-TTTG--ATGCCAGTATCAAACGC-----TTACGTGTCAA-
CGGCAATCAAGACTGACTTCCATCGCAGGCAGACCATCTCAGGCGAGCACGGCCTCGACAGCAATGGCGTGTACGTGACTATCGCCGAC
CCAAA-TTCTCATAA--TCCC---

TCGCTCACCGCTACAAATAGGTACAACGGCACCTCGGAGCTCCAGCTCGAGCGCATGAATGTCTACTTCAACGAGGTGAGTCGGACCCGT
ACACG-TTTTTCAGTTATCT----CGAAGTTTGC-GGTTGCTGACAATATA----
TTCTTCGCAGGCTTCTGGCAACAAATATGTCCCTCGCGCCGTCCTCGTCGACTTGGAGCCCGGCACCA?????????????????????????????????????
?????????????????

>Amesia_atrobrunnea_CBS_250_75

ATTACAGAGTTGC-AAAACTCCCTAAACCATTGTGAACGTTACCCTT-AACCGTTGCTTCGGCGGGCGGCGCCAT----------------------------
GCCGCCCCTCGGCCCTTC----------------------CCGGGCGCCCGCCGGAGGATACCC-AACTCTTGATATTTT--
ATGGCCACTCTGAGTCTTCTGTACTGAA-
TAAGTCAAAACTTTCAACAACGGATCTCTTGGTTCTGGCATCGATGAAGAACGCAGCGAAATGCGATAAGTAATGTGAATTGCAGAATTC
AGTGAATCATCGAATCTTTGAACGCACATTGCGCCCGCCAGTATTCTGGCGGGCATGCCTGTTCGAGCGTCATTTCAACCATCAAGCCCCC
-GGCTTGTGTTGGGGACCTGCG----------GCTGCCCGCAGGCCCTGAAAAACAGTGGCGGGCTCGCT-GTCACACCGAGCGTAGTAG--
CATCATTCTCGCTCAGGGCG-TGCTGCGGGTT--CCGGCCGTTAAA-
CGACCTTCATAACCCAAGGTTGACCTCGGATCAGGTAGGAAGACCCGCTGAACTTAA?????????????????????????????????????TGCCC-
TAGTAACGGCG-AGTGAAGCGGCAACAGCTCAAATTTGAAATCTGGCTTCGGCCCGAGTTGTAATTTGTAG-
AGGAAGCTTTAGGCGCGGCACCTTCTGAGTCCCCTGGAACGGGGCGCCATAGAGGGTGAGAGCCCCGTATAGTTGGATGCCTAGCCTGT
GTAAAGCTCC-
TTCGACGAGTCGAGTAGTTTGGGAATGCTGCTCAAAATGGGAGGTAAATTTCTTCTAAAGCTAAATACCGGCCAGAGACCGATAGCGCA
CAAGTAGAGTGATCGAAAGATGAAAAGCACTTTGAAAAGAGGGTTAAATAGCACGTGAAATTGTTGAAAGGGAAGCGCTTGTGACCAG
ACTTGCGCCGGGCTGATCATCCGGTGTTCTCACCGGTGCACTCTGCCCGGCTCAGGCCAGCATCGGTTCTCGCGGGGGGATAAAGGCCCA
GGGAATGTAGCTCCTCCGGGAGTGTTATAGCCCGGGGCGCAATGCCCTCGCGGGGACCGAGGTTCGCGC-
TCTGCAAGGATGCTGGCGTAATGGTCATCAGCGACCCGTCTTGAAACACGGACCAAGGAGTCAAGGTTTTGCGCGAGTGTTTGGGTGTAA
AACCCGCACGCGTAATGAAAGTGAACGTAGGTGAGAGCTTCGGCGCATCATCGACCGATCCTGATGTTTTCGGATGGATTTGAGTAAGAG
CGTTAAGCCTTGGACCCGAAAGATGGTGAACTATGCTTGGATAGGGTGAAGCCAGAGGAAACTCTGGTGGAGGCTCGCAGCGG-
TTCTGACGTGCAAATCGATCGTCAAATCTGAGCATCTGGTCACACTGGTGCAGGGTTTGCGGAGGAAGAACGTCATTTCGTTCGAGGTTTC
GCTGGTTCGCGACATCCGCGACCGCGAGTTCAAGATCTTCTCAGATGCCGGGCGCGTCATGAGGCCGCTGTTTACCGTCGAGCAGGAGCA
TGGTTCAGAGACCGGCGCCGAGATGGGCCAGCTCATCCTCAACAAAGAGCATATTACACGGTTAGAAGCCGACAAGGAGCTGGGCAAG
TACCATCCCGACTACTGGGGCTGGCAGGGCCTGCTGAAGTCGGGTGCCATCGAGTATCTCGATGCCGAGGAGGAGGAGACAGCCATGAT

TTGCATGACGCCCGAGGATCTCGACAAGTTCCGGTACAGAAAGATGGGGTTCATCGTCGAAGACAACTCGGGTCAAGGTAACAACAGGA
TCAAGACGAAGCCGAACCCGGCCACCCACATGTACACGCACTGCGAGATCCATCCCAGCATGCTGCTCGGCATCTGCGCAAGCATCATC
CCCTTCCCCGACCACAACCAGACGCGTCGGGAAACT---TTTTT-GGAACCCCTGAT---CTACCC--CACACGTTGGGA-GGACCTCGTT---TC-
CCAGCT--ATTCGA----CCGACC----GAGAGCGATGATGGCACGGCCATGA--TAGAAA-----GAACACA-GTGCTGACGT--
TGCTTCTCTACTACAGGTTCACCTCCAGACCGGCCAATGCGTAAGTTGGATCGATTC--GAGC----TCGACGACC-
GATGCCGTGTTTGTGATGGTGC------GGGTGTAGACTGA---CCAGCT-CTCCAGGGTAACCAAATTGGTGCCGCTTTCTGGTATG-TTCG--
ATGCCAACATCAAGCTC-----TTGCGCGTCTG-
AGGCAACCAAGACTGACTTCCATCACAGGCAGACCATCGCCGGCGAGCACGGCCTCGACAGCAATGGCGTGTACGTGACTGTCGCCGAC
CCAGA-TTCTGATAA--TCCC---TCGCTCACCGCTAC-
GATAGGTACAACGGCACTTCGGAGCTCCAGCTCGAGCGCATGAACGTCTACTTCAATGAGGTGAGTCGGGCCCGTACACC-
TTCATCAGATATCT----TGAATGCTGC-GGTCGCTGACAACATA----
TTCTTGGCAGGCTTCCGGCAACAAGTATGTCCCTCGTGCTGTCCTTGTCGACTTGGAGCCCGGCACCATGGATGCCGTCCGCGTTGGTCCC
TTCGGCCAGCTCTTCCGCCCTGACAACTT

>Amesia_atrobrunnea_CBS_379_66

ATTACAGAGTTGC-AAAACTCCCTAAACCATTGTGAACGTTACCCTT-AACCGTTGCTTCGGCGGGCGGCGCCAT---------------------------
GCCGCCCCTCGGCCCTTC---------------------CCGGGCGCCCGCCGGAGGATACCC-AACTCTTGATATTTT--
ATGGCCACTCTGAGTCTTCTGTACTGAA-
TAAGTCAAAACTTTCAACAACGGATCTCTTGGTTCTGGCATCGATGAAGAACGCAGCGAAATGCGATAAGTAATGTGAATTGCAGAATTC
AGTGAATCATCGAATCTTTGAACGCACATTGCGCCCGCCAGTATTCTGGCGGGCATGCCTGTTCGAGCGTCATTTCAACCATCAAGCCCCC
-GGCTTGTGTTGGGGACCTGCG----------GCTGCCCGCAGGCCCTGAAAAACAGTGGCGGGCTCGCT-GTCACACCGAGCGTAGTAG--
CATCATTCTCGCTCAGGGCG-TGCTGCGGGTT--CCGGCCGTTAAA-
CGACCTTCATAACCCAAGGTTGACC??CCC-TAGTAACGGCG-
AGTGAAGCGGCAACAGCTCAAATTTGAAATCTGGCTTCGGCCCGAGTTGTAATTTGTAG-
AGGAAGCTTTAGGCGCGGCACCTTCTGAGTCCCCTGGAACGGGGCGCCATAGAGGGTGAGAGCCCCGTATAGTTGGATGCCTAGCCTGT
GTAAAGCTCC-
TTCGACGAGTCGAGTAGTTTGGGAATGCTGCTCAAAATGGGAGGTAAATTTCTTCTAAAGCTAAATACCGGCCAGAGACCGATAGCGCA
CAAGTAGAGTGATCGAAAGATGAAAAGCACTTTGAAAAGAGGGTTAAATAGCACGTGAAATTGTTGAAAGGGAAGCGCTTGTGACCAG

ACTTGCGCCGGGCTGATCATCCGGTGTTCTCACCGGTGCACTCTGCCCGGCTCAGGCCAGCATCGGTTCTCGCGGGGGGATAAAGGCCCA
GGGAATGTAGCTCCTCCGGGAGTGTTATAGCCCGGGGCGCAATGCCCTCGCGGGGACCGAGGTTCGCGC-
TCTGCAAGGATGCTGGCGTAATGGTCATCAGCGACCCGTCTTGAAACACGGACCAAGGAGTCAAGGTTTTGCGCGAGTGTTTGGGTGTAA
AACCCGCACGCGTAATGAAAGTGAACGTAGGTGAGAGCTTCGGCGCATCATCGACCGATCCTGATGTTTTCGGATGGATTTGAGTAAGAG
CGTTAAGCCTTGGACCCGAAAGATGGTGAACTATGCTTGGATAGGGTGAAGCCAGAGGAAACTCTGGTGGAGGCTCGCAGCGG-
TTCTGACGTGCAAATCGATCGTCAAATCTGAGCATCTGGTCACACTGGTGCAGGGTTTGCGGAGGAAGAACGTCATTTCGTTCGAGGTTTC
GCTGGTTCGCGACATCCGCGACCGCGAGTTCAAGATCTTCTCAGATGCCGGGCGCGTCATGAGGCCGCTGTTTACCGTCGAGCAGGAGCA
TGGTTCAGAGACCGGCGCCGAGATGGGCCAGCTCATCCTCAACAAAGAGCATATTACACGGTTAGAAGCCGACAAGGAGCTGGGCAAG
TACCATCCCGACTACTGGGGCTGGCAGGGCCTGCTGAAGTCGGGTGCCATCGAGTATCTCGATGCCGAGGAGGAGGAGACAGCCATGAT
TTGCATGACGCCCGAGGATCTCGACAAGTTCCGGTACAGAAAGATGGGGTTCATCGTCGAAGACAACTCGGGTCAAGGTAACAACAGGA
TCAAGACGAAGCCGAACCCGGCCACCCACATGTACACGCACTGCGAGATCCATCCCAGCATGCTGCTCGGCATCTGCGCAAGCATCATC
CCCTTCCCCGACCACAACCAGACGCGTCGGGAAGCT---TTTTT-GGAACCCCTGAT---CTACCC--CACACGTTGGGA-GGACCTCGTT---TC-
CCAGCT--ATTCGA----CCGACC----GAGAGCGATGATGGCACTGCCATGA--TAGAAA-----GAACACA-GTGCTGACGT--
TGCTTCTCTACTACAGGTTCACCTCCAGACCGGCCAATGCGTAAGTTGGATCGATTT--GAGC----CCGACGACC-
GATGCCGTGTTTGTGATGGTGC------GGGTGTAGACTGA---CCAGCT-CTCCAGGGTAACCAAATTGGTGCCGCTTTCTGGTATG-TTCG--
ATGCCAACATCAAGCTC-----TTGCGCGTCTG-
AGGCAACCAAGACTGACTTCCATCACAGGCAGACCATCGCCGGCGAGCACGGCCTCGACAGCAATGGCGTGTACGTGACTGTCGCCGAC
CCAGA-CTCTGATAA--TCCC---TCGCTCACCGCTAC-
GATAGGTACAACGGCACTTCGGAGCTCCAGCTCGAGCGCATGAACGTCTACTTCAATGAGGTGAGTCGGGCCCGTACACC-
TTCATCAGATATCT----TGAATGCTGC-GGTCGCTGACAACATA----
TTCTTGGCAGGCTTCCGGCAACAAGTATGTCCCTCGTGCGGTCCTTGTCGACTTGGAGCCCGGCACCATGGATGCCGTCCGCGTTGGTCCC
TTCGGCCAGCTCTTCCGCCCTGACAACTT

>Amesia_cymbiformis_CBS_175_84

ATTACAGAGTTGC-AAAACTCCCTAAACCATTGTGAACGTTACCCTT-AACCGTTGCTTCGGCGGGCGGCGCCAT---------------------------
GCCGCCCCTCGGCCCTTC---------------------CCGGGCGCCCGCCGGAGGATACCC-AACTCTTGATATTTT--
ATGGCCACTCTGAGTCTTCTGTACTGAA-
TAAGTCAAAACTTTCAACAACGGATCTCTTGGTTCTGGCATCGATGAAGAACGCAGCGAAATGCGATAAGTAATGTGAATTGCAGAATTC

AGTGAATCATCGAATCTTTGAACGCACATTGCGCCCGCCAGTATTCTGGCGGGCATGCCTGTTCGAGCGTCATTTCAACCATCAAGCCCCC
-GGCTTGTGTTGGGGACCTGCG----------GCTGCCCGCAGGCCCTGAAAAACAGTGGCGGGCTCGCT-GTCACACCGAGCGTAGTAG--
CATCATTCTCGCTCAGGGCG-TGCTGCGGGTT--CCGGCCGTTAAA-
CGACCTTCATAACCCAAGGTTGACCTCGGATCAGGTAGGAAGACCCGCTGAACTTAA???TTGCCC-
TAGTAACGGCG-AGTGAAGCGGCAACAGCTCAAATTTGAAATCTGGTTTCGGCCCGAGTTGTAATTTGCAG-
AGGAAGCTTTAGGCGCGGCACCTTCTGAGTCCCCTGGAACGGGGCGCCATAGAGGGTGAGAGCCCCGTATAGTTGGATGCCTAGCCTGT
GTAAAGCTCCTTTCGACGAGTCGAGTAGTTTGGGAATGCTGCTCAAAATGGGAGGTAAATTTCTTCTAAAGCTAAATACCGGCCAGAGAC
CGATAGCGCACAAGTAGAGTGATCGAAAGATGAAAAGCACTTTGAAAAGAGGGTTAAATAGCACGTGAAATTGTTGAAAGGGAAGCGC
TTGTGACCAGACTTGCGCCGGGCTGATCATCCGGTGTTCTCACCGGTGCACTCTGCCCGGCTCAGGCCAGCATCGGTTCTCGCGGGGGAT
AAAGGCCCAGGGAATGTAGCTCCTCCGGGAGTGTTATAGCCCGGGGCGGAATGCCCTCGCGGGGACCGAGGTTCGCGC-
TCTGCAAGGATGCTGGCGTAATGGTCATCAGCGACCCGTCTTGAAACACGGACCAAGGAGTCAAGGTTTTGCGCGAGTGTTTGGGTGTAA
AACCCGCACGCGTAATGAAAGTGAACGTAGGTGAGAGCTTCGGCGCATCATCGACCGATCCTGATGTTTTCGGATGGATTTGAGTAAGAG
CGTTAAGCCTTGGACCCGAAAGATGGTGAACTATGCTTGGATAGGGTGAAGCCAGAGGAAACTCTGGTGGAGGCTCGCAGCGG-
TTCTGACGTGCAAATCGATCGTCAAATCTGAGCATCTGGTCACACTGGTGCAGGGTCTGCGGAGGAAGAACGTCATTTCGTTCGAGGTTTC
GTTGGTTCGCGACATCCGCGACCGCGAGTTCAAGATCTTTTCAGATGCCGGGCGCGTCATGAGGCCGTTGTTTACCGTCGAGCAGGAGCA
TGGTTCAGAGACCGGCGCCGAAATGGGCCAGCTCATACTCAACAAAGAGCATATTACACGGTTAGAAGCGGACAAGGAGCTGGGCAAG
TACCATCCCGACTACTGGGGCTGGCAGGGCCTGCTGAAGTCGGGTGCCATCGAGTATCTCGATGCCGAGGAGGAGGAGACAGCCATGAT
TTGCATGACGCCCGAGGATCTCGACAAGTTCCGGTACAGGAAGATGGGGTTCATCGTCGAAGACAACTCGGGTCAAGGTAACAACAGGA
TCAAGACGAAGCCGAACCCGGCCACCCATATGTACACACACTGCGAGATCCATCCCAGCATGCTGCTCGGCATCTGCGCCAGCATCATTC
CCTTCCCCGACCACAACCAGACGCGTCGGGAAGCT---TTTTTGGGGGCCTCTGAT---CTACCC--CACACGTTGGGT-CGACCTCGCT---
TCATTAGCT--ATTCGA---CCCGACC----GAGAGCGATGACGGCACGGCCATGA--TAGAAA-----GGACACG-GTGCTGACAT--
TGCTTCTCTACTACAGGTTCACCTCCAGACCGGCCAATGCGTAAGTTGGATCTATTC--GAGC----CCGACGACC-
GATGCCGTGTTCGTGATGGTGC------GGGTATAGACTGA---CCAGTT-CTCCAGGGTAACCAAATTGGTGCCGCTTTCTGGTATG-TTCG--
ATGCCAACATCAAGCTC-----TTGCGTGTCTG-
AGGCAATCAAGACTGACTTCCATCACAGGCAGACCATCTCTGGCGAGCACGGCCTCGACAGCAATGGCGTGTACGTAACTGTCGCTGAC
CCAGA-TTCTGATAA--TCCC---TCGCTCACCGCTGC-
GATAGGTACAACGGCACTTCGGAGCTCCAGCTCGAGCGCATGAACGTCTACTTCAACGAGGTGAGTCGGG-CTGTACACC-
TTGATCAGTTATTT----TGAATGCTGC-GGTTTCTGACAACATAT---

TTCTTCGCAGGCTTCCGGCAACAAGTATGTCCCTCGTGCCGTCCTCGTCGACTTGGAGCCCGGCACCATGGATGCCGTTCGCGCTGGTCCC
TTCGGCCAGCTCTTCCGCCCTGACAACTT

>Chaetomium_dreyfussii_CBS_376.83

ATTACAGAGTTGC-AAAACTCCCTAAACCATTGTGAACGTTACC-TTCAACCGTTGCTTCGGCGGGCGGCGCTCT---------------------------
GCCGCCCCTCGGCCCCGC---------------------CCGGGCGCCCGCCGGAGGATACCC-AACTCTTGATACTTT--
ATGGCCTCTCTGAGTCTTCTGTACTGAA-
TAAGTCAAAACTTTCAACAACGGATCTCTTGGTTCTGGCATCGATGAAGAACGCAGCGAAATGCGATAAGTAATGTGAATTGCAGAATTC
AGTGAATCATCGAATCTTTGAACGCACATTGCGCCCGCCAGTATTCTGGCGGGCATGCCTGTTCGAGCGTCATTTCAACCATCAAGCCTAC
-GGCTTGTGTTGGGGACCTGCG----------GCTGCCCGCAGGCCCTGAAAACCAGTGGCGGGCTCGCT-GTCACACCGAGCGTAGTAG--
CATTATTCTCGCTCAGGGCG-TGCTGCGGGTT--CCGGCCGTTAAA-
CGACCTTCATAACCCAAGGTTGACCTC????????????????????????????????????CAATAAGCGGAGGAAAAGAAACCAACAGGGATTGCCC-
TAGTAACGGCG-AGTGAAGCGGCAACAGCTCAAATTTGAAATCTGGCTTCGGCCCGAGTTGTAATTTGTAG-
AGGAAGCTTTAGGCGCGGCACCTTCTGAGTCCCCTGGAACGGGGCGCCATAGAGGGTGAGAGCCCCGTATAGTTGGATGCCTAGCCTGT
GTAAAGCTCC-
TTCGACGAGTCGAGTAGTTTGGGAATGCTGCTCAAAATGGGAGGTAAATTTCTTCTAAAGCTAAATACCGGCCAGAGACCGATAGCGCA
CAAGTAGAGTGATCGAAAGATGAAAAGCACTTTGAAAAGAGGGTTAAATAGCACGTGAAATTGTTGAAAGGGAAGCGCTTGTGACCAG
ACTTGCGTCGGGCTGATCATCCGGTGTTCTCACCGGTGCACTCTGCCCGGCTCAGGCCAGCATCGGTTCTCGCGGGGGGATAAAGGTTCC
GGGAATGTAGCTCCTCCGGGAGTGTTATAGCCCGGGGCGTAATGCCCTCGCGGGGACCGAGGTTCGCGC-
TCTGCAAGGATGCTGGCGTAATGGTCATCAGCGACCCGTCTTGAAACACGGACCAAGGAGTCAAGGTTTTGCGCGAGTGTTTGGGTGTAA
AACCCGCACGCGTAATGAAAGTGAACGTAGGTGAGAGCTTCGGCGCATCATCGACCGATCCTGATGTTTTCGGATGGATTTGAGTAAGAG
CGTTAAGCCTTGGACCCGAAAGATGGTGAACTATGCTTGGATAGGGTGAAGCCAGAGGAAACTCTGGTGGAGGCTCGCAGCGG-
TTCTGACGTGCAAATCGATCGTCAAATCTGAGCATCTGGTCACGTTGGTACAGGGTCTGCGGAGGAAGAACGTCATTTCGTTCGAGGTGT
CGTTGGTTCGCGACATCCGCGACCGCGAGTTCAAGATCTTTTCCGATGCTGGGCGCGTCATGAGGCCGCTGTTTACCGTTGAGCAGGAGC
ACGGTTCAGAAACTGGCGCCGAAATGGGGCAGCTCATCCTCAACAAGGAGCATATCACACGCTTGGAAGCGGACAAGGAGCTGGGCAA
ATACCATCCCGACTACTGGGGCTGGCAGGGCCTGCTGAAGTCGGGCGCCATCGAGTATCTCGATGCCGAGGAAGAGGAGACGGCCATGA
TTTGCATGACGCCCGAGGACCTGGACAAGTTCCGGTACAGAAAGATGGGATTCATCGTCGAAGACAACTCGGGTCAAGGTAACAATAGG
ATCAAGACGAAGCCGAACCCCGCCACCCACATGTACACGCACTGCGAGATCCATCCCAGCATGTTGCTCGGCATCTGCGCCAGCATCAT

CCCCTTCCCTGACCACAACCAGACGCGTCGGGAAGTT---TTCTT-TGGGCCCCTGAT---CTACCC--CACACATTGGGA-TGACCTCGTT---
CCACCAGCT--AGCTACTCAACCGACC----GAGAGCGATGACGGCGCGGCCATGA--TGGACA-----GGACACG-GTGCTGACAT--
TACTTCTCTACTACAGGTTCACCTCCAGACCGGTCAATGCGTAAGTTGGATCGATTC--GAGC----ACGACGACC-AATACCGTGTG-
GTGATGGTGC------GGGTGAAGACTGACAACAACCT-CTCCAGGGTAACCAAATCGGTGCTGCTTTCTGGTATG-TTCG--
ATGCCAGCATCAAACGC-----TTGCGTGTCAA-
GGGCAATCAGGACTGACTTCCATCGCAGGCAGACCATCTCAGGCGAGCACGGCCTCGACAGCAATGGCGTGTACGTGACTGTCGCCGAC
CCAAA-TTCTCATAA--TCCC---
TTGCTCACCGCTACAAATAGGTACAACGGCACCTCGGAGCTCCAGCTCGAGCGCATGAATGTCTACTTCAACGAGGTGAGTCTGGCCCGT
ACACG-TTCTTCAGTTATCT----CGAAGTTTGC-GGTCGCTGACAACAGA----
TTCTTCGCAGGCTTCCGGCAACAAATATGTCCCTCGCGCCGTCCTTGTCGACTTGGAGCCCGGCACCATGGATGCCGTTCGCGCTGGTCCC
TTCGGCCAGCTCTTCCGCCCTGACAGCTT

>Amesia_cymbiformis_CBS_176_84

ATTACAGAGTTGC-AAAACTCCCTAAACCATTGTGAACGTTACCCTT-AACCGTTGCTTCGGCGGGCGGCGCCAT---------------------------
GCCGCCCCTCGGCCCTTC---------------------CCGGGCGCCCGCCGGAGGATACCC-AACTCTTGATATTTT--
ATGGCCACTCTGAGTCTTCTGTACTGAA-
TAAGTCAAAACTTTCAACAACGGATCTCTTGGTTCTGGCATCGATGAAGAACGCAGCGAAATGCGATAAGTAATGTGAATTGCAGAATTC
AGTGAATCATCGAATCTTTGAACGCACATTGCGCCCGCCAGTATTCTGGCGGGCATGCCTGTTCGAGCGTCATTTCAACCATCAAGCCCCC
-GGCTTGTGTTGGGGACCTGCG----------GCTGCCCGCAGGCCCTGAAAAACAGTGGCGGGCTCGCT-GTCACACCGAGCGTAGTAG--
CATCATTCTCGCTCAGGGCG-TGCTGCGGGTT--CCGGCCGTTAAA-
CGACCTTCATAACCCAAGGTTGACCTCGGATCAGGTAGGAAGACCCGCTGAACTTAAGCATATCAATAAGCGGAGGAAAAGAAACCAA
CAGGGATTGCCC-TAGTAACGGCG-AGTGAAGCGGCAACAGCTCAAATTTGAAATCTGGCTTCGGCCCGAGTTGTAATTTGCAG-
AGGAAGCTTTAGGCGCGGCACCTTCTGAGTCCCCTGGAACGGGGCGCCATAGAGGGTGAGAGCCCCGTATAGTTGGATGCCTAGCCTGT
GTAAAGCTCC-
TTCGACGAGTCGAGTAGTTTGGGAATGCTGCTCAAAATGGGAGGTAAATTTCTTCTAAAGCTAAATACCGGCCAGAGACCGATAGCGCA
CAAGTAGAGTGATCGAAAGATGAAAAGCACTTTGAAAAGAGGGTTAAATAGCACGTGAAATTGTTGAAAGGGAAGCGCTTGTGACCAG
ACTTGCGCCGGGCTGATCATCCGGTGTTCTCACCGGTGCACTCTGCCCGGCTCAGGCCAGCATCGGTTCTCGCGGGGGGATAAAGGCCCA
GGGAATGTAGCTCCTCCGGGAGTGTTATAGCCCGGGGCGGAATGCCCTCGCGGGGACCGAGGTTCGCGCATCTGCAAGGATGCTGGCGT

AATGGTCATCAGCGACCCGTCTTGAAACACGGACCAAGGAGTCAAGGTTTTGCGCGAGTGTTTGGGTGTAAAACCCGCACGCGTAATGA
AAGTGAACGTAGGTGAGAGCTTCGGCGCATCATCGACCGATCCTGATGTTTTCGGATGGATTTGAGTAAGAGCGTTAAGCCTTGGACCCG
AAAGATGGTGAACTATGCTTGGATAGGGTGAAGCCAGAGGAAACTCTGGTGGAGGCTCGCAGCGG-
TTCTGACGTGCAAATCGATCGTCAAATCTGAGCATCTGGTCACACTGGTGCAGGGTCTGCGGAGGAAGAACGTCATTTCGTTCGAGGTTTC
GTTGGTTCGCGACATCCGCGACCGCGAGTTCAAGATCTTTTCAGATGCCGGGCGCGTCATGAGGCCGTTGTTTACCGTCGAGCAGGAGCA
TGGTTCAGAGACCGGCGCCGAAATGGGCCAGCTCATACTCAACAAAGAGCATATTACACGGTTAGAAGCGGACAAGGAGCTGGGCAAG
TACCATCCCGACTACTGGGGCTGGCAGGGCCTGCTGAAGTCGGGTGCCATCGAGTATCTCGATGCCGAGGAGGAGGAGACAGCCATGAT
TTGCATGACGCCCGAGGATCTCGACAAGTTCCGGTACAGGAAGATGGGGTTCATCGTCGAAGACAACTCGGGTCAAGGTAACAACAGGA
TCAAGACGAAGCCGAACCCGGCCACCCATATGTACACACACTGCGAGATCCATCCCAGCATGCTGCTCGGCATCTGCGCCAGCATCATTC
CCTTCCCCGACCACAACCAGACGCGTCGGGAAGCT---TTTTTGGGGGCCTCTGAT---CTACCC--CACACGTTGGGT-CGACCTCGCT---
TCATTAGCT--ATTCGA---CCCGACC----GAGAGCGATGACGGCACGGCCATGA--TAGAAA-----GGACACG-GTGCTGACAT--
TGCTTCTCTACTACAGGTTCACCTCCAGACCGGCCAATGCGTAAGTTGGATCTATTC--GAGC----CCGACGACC-
GATGCCGTGTTCGTGATGGTGC------GGGTATAGACTGA---CCAGTT-CTCCAGGGTAACCAAATTGGTGCCGCTTTCTGGTATG-TTCG--
ATGCCAACATCAAGCTC-----TTGCGTGTCTG-
AGGCAATCAAGACTGACTTCCATCACAGGCAGACCATCTCTGGCGAGCACGGCCTCGACAGCAATGGCGTGTACGTAACTGTCGCTGAC
CCAGA-TTCTGATAA--TCCC---TCGCTCACCGCTGC-
GATAGGTACAACGGCACTTCGGAGCTCCAGCTCGAGCGCATGAACGTCTACTTCAACGAGGTGAGTCGGG-CTGTACACC-
TTGATCAGTTATTT----TGAATGCTGC-GGTTTCTGACAACATAT---
TTCTTCGCAGGCTTCCGGCAACAAGTATGTCCCTCGTGCCGTCCTCGTCGACTTGGAGCCCGGCACCATGGATGCCGTTCGCGCTGGTCCT
TTCGGCCAGCTCTTCCGCCCTGACAACTT

>Amesia_gelasinospora_CBS_643_83

ATTACAGAGTTGC-AAAACTCCCTAAACCATTGTGAACGTTACC-TT-AACCGTTGCTTCGGCGGGCGGCGCCCTC--------------------------
GGCGCCCCTCGGCCCACC---------------------CCGGGCGCCCGCCGGAGGATACCC-AACTCTTGATACTTT--
ATGGCCACTCTGAGTCTTCTGTACTGAA-
TAAGTCAAAACTTTCAACAACGGATCTCTTGGTTCTGGCATCGATGAAGAACGCAGCGAAATGCGATAAGTAATGTGAATTGCAGAATTC
AGTGAATCATCGAATCTTTGAACGCACATTGCGCCCGCCAGTATTCTGGCGGGCATGCCTGTTCGAGCGTCATTTCAACCATCAAGCCCCC
-GGCTTGTGTTGGGGACCTGCG----------GCTGCCCGCAGGCCCTGAAAACCAGTGGCGGGCTCGCT-GTCACACCGAGCGTAGTAG--

CATAATTCTCGCTCAGGGCG-TGCTGCGGGTT--CCGGCCGTTAAA-
CGACCTTCATAACCCAAGGTTGACCTCGGATCAGGTAGGAAGACCCGCTGAACTTAAGCATATCAATAAGCGGAGGAAAAGAAACCAA
CAGGGATTGCCC-TAGTAACGGCG-AGTGAAGCGGCAACAGCTCAAATTTGAAATCTGGCCTCGGCCCGAGTTGTAATTTGCAG-
AGGAAGCTTTAGGCGCGGCACCTTCTGAGTCCCCTGGAACGGGGCGCCATAGAGGGTGAGAGCCCCGTATAGTTGGATGCCTAGCCTGT
GTAAAGCTCC-
TTCGACGAGTCGAGTAGTTTGGGAATGCTGCTCAAAATGGGAGGTAAATTTCTTCTAAAGCTAAATACCGGCCAGAGACCGATAGCGCA
CAAGTAGAGTGATCGAAAGATGAAAAGCACTTTGAAAAGAGGGTTAAATAGCACGTGAAATTGTTGAAAGGGAAGCGCTTGTGACCAG
ACTTGCGCCGGGCTGATCATCCGGTGTTCTCACCGGTGCACTCTGCCCGGCTCAGGCCAGCATCGGTTCTCGCGGGGGGATAAAGGCCCA
GGGAATGTAGCTCCTCCGGGAGTGTTATAGCCCGGGGTGCAATGCCCTCGCGGGGACCGAGGTTCGCGCATCTGCAAGGATG????????????
??
???CTGGTCACGC
TGGTGCAGGGCCTGCGGAGGAAGAACGTCATTTCCTTCGAGGTTTCGCTGGTTCGCGACATCCGCGACCGCGAGTTCAAGATTTTCTCGG
ATGCTGGACGCGTCATGAGGCCGCTGTTTACTGTCGAGCAGGAGCACGGTTCAGACTCGGGCGCCGAAATGGGCCAGCTCATCCTCAAC
AAGGAGCACATTACGCGGTTAGAAACGGACAAGGAGCTGGGCAAATACCACCCCGACTACTGGGGCTGGCAGGGCCTCTTGAAGTCAG
GTGCGATCGAGTACCTCGATGCCGAGGAGGAGGAGACGGCCATGATTTGCATGACGCCCGAAGACCTCGACAAGTTCCGGTACAGAAA
AATGGGGTTCATCGTCGAAGACAACTCAGGTCAAGGTAACAACAGGATCAAGACGAAGCCTAACCCCGCCACCCACACGTACACGCAC
TGCGAGATTCACCCCAGCATGCTGCTCGGCATCTGCGCCAGCATCATTCCCTTCCCGGACCACAACCAGACGCGTCGGGAAGCT---TTTTT-
TGGGCCCTTGAT---CTACCC--CACACATTGGGAGCGACCTCGTT---CCACCAGCT--ACCCGA----CCGACCG--
AGAGAGCGATGACGGCGCGGCTATGA--TGGAAA-----GGACACG-GTGCTGACAT--
TACTCTTCTACTACAGGTTCACCTCCAGACCGGCCAGTGCGTAAGTAGAATCGATTC--GAGC----CCGACGACC-GATACGGTGTG-
GTGGTGGTGCTACTATGGGCGAAGACTGACAACCAGCT-CTCCAGGGTAACCAAATCGGTGCCGCTTTCTGGTATGTTTTG--
ATGCCAACTCCATCCGT-----
TTGCGTGTCTATGGGCAATGAAGACTGACTTCTATCACAGGCAGACCATCTCTGGCGAGCACGGCCTCGACAGCAATGGCGTGTACGTGA
CTGTCGCCGACCCAAACCTCCGATAA--TCCC---TCGCTCACCGCTAT-
GATAGGTACAACGGCACCTCGGAGCTCCAGCTCGAGCGCATGAACGTCTACTTCAACGAGGTGAGTCGCGCTCATACACC-
GTCATATGTTGCTT----TCGAGGCTGC-GGTCGCTGACAATATA----
TGTTTTTCAGGCTTCCGGCAACAAGTATGTCCCTCGTGCCGTCCTCGTCGACTTGGAGCCCGGCACCATGGATGCCGTCCGCGCTGGTCCC
TTCGGCCAGCTCTTCCGCACCGACAACTT

>Amesia_gelasinospora_CBS_673_80

ATTACAGAGTTGC-AAAACTCCCTAAACCATTGTGAACGTTACC-TT-AACCGTTGCTTCGGCGGGCGGCGCCCTC------------------------------
GGCGCCCCTCGGCCCACC------------------------CCGGGCGCCCGCCGGAGGATACCC-AACTCTTGATACTTT--
ATGGCCACTCTGAGTCTTCTGTACTGAA-
TAAGTCAAAACTTTCAACAACGGATCTCTTGGTTCTGGCATCGATGAAGAACGCAGCGAAATGCGATAAGTAATGTGAATTGCAGAATTC
AGTGAATCATCGAATCTTTGAACGCACATTGCGCCCGCCAGTATTCTGGCGGGCATGCCTGTTCGAGCGTCATTTCAACCATCAAGCCCCC
-GGCTTGTGTTGGGGACCTGCG----------GCTGCCCGCAGGCCCTGAAAACCAGTGGCGGGCTCGCT-GTCACACCGAGCGTAGTAG--
CATAATTCTCGCTCAGGGCG-TGCTGCGGGTT--CCGGCCGTTAAA-
CGACCTTCATAACCCAAGGTTGACCTCGGATCAGGTAGGAAGACCCGCTGAACTTAA??????CAATAAGCGGAGGAAAAGAAACCAACA
GGGATTGCCC-
TAGTAACGGCGAAGTGAAGCGGCAACAGCTCAAATTTGAAATCTGGCCTCGGCCCGAGTTGTAATTTGCAGAAGGAAGCTTTAGGCGCG
GCACCTTCTGAGTCCCCTGGAACGGGGCGCCATAGAGGGTGAGAGCCCCGTATAGTTGGATGCCTAGCCTGTGTAAAGCTCC-
TTCGACGAGTCGAGTAGTTTGGGAATGCTGCTCAAAATGGGAGGTAAATTTCTTCTAAAGCTAAATACCGGCCAGAGACCGATAGCGCA
CAAGTAGAGTGATCGAAAGATGAAAAGCACTTTGAAAAGAGGGTTAAATAGCACGTGAAATTGTTGAAAGGGAAGCGCTTGTGACCAG
ACTTGCGCCGGGCTGATCATCCGGTGTTCTCACCGGTGCACTCTGCCCGGCTCAGGCCAGCATCGGTTCTCGCGGGGGGATAAAGGCCCA
GGGAATGTAGCTCCTCCGGGAGTGTTATAGCCCGGGGTGCAATGCCCTCGCGGGGACCGAGGTTCGCGCATCTGCAAGGATGCTGGCGT
AATGGTCATCAGCGACCCGTCTTGAAACACGGACCAAGGAGTCAAGGTTTTGCGCGAGTGTTTGGGTGTAAAACCCGCACGCGTAATGA
AAGTGAACGTAGGTGAGAGCTTCGGCGCATCATCGACCGATCCTGATGTTTTCGGATGGATTTGAGTAAGAGCGTTAAGCCTTGGACCCG
AAAGATGGTGAACTATGCTTGGATAGGGTGAAGCCAGAGGAAACTCTGGTGGAGGCTCGCAGCGG-
TTCTGACGTGCAAATCGATCGTCAAATCTGAGCATCTGGTCACGCTGGTGCAGGGCCTGCGGAGGAAGAACGTCATTTCCTTCGAGGTTTC
GCTGGTTCGCGACATCCGCGACCGCGAGTTCAAGATTTTCTCGGATGCTGGACGCGTCATGAGGCCGCTGTTTACTGTCGAGCAGGAGCA
CGGTTCAGACTCGGGCGCCGAAATGGGCCAGCTCATCCTCAACAAGGAGCACATTACGCGGTTAGAAACGGACAAGGAGCTGGGCAAA
TACCACCCCGACTACTGGGGCTGGCAGGGCCTCTTGAAGTCAGGTGCGATCGAGTACCTCGATGCCGAGGAGGAGGAGACGGCCATGAT
TTGCATGACGCCCGAAGACCTCGACAAGTTCCGGTACAGAAAAATGGGGTTCATCGTCGAAGACAACTCAGGTCAAGGTAACAACAGG
ATCAAGACGAAGCCTAACCCCGCCACCCACACGTACACGCACTGCGAGATTCACCCCAGCATGCTGCTCGGCATCTGCGCCAGCATCAT
TCCCTTCCCGGACCACAACCAGACGCGTCGGGAAGCT---TTTTT-TGGGCCCTTGAT---CTACCC--CACACATTGGGAGCGACCTCGTT---
CCACCAGCT--ACCCGA----CCGACCG--AGAGAGCGATGACGGCGCGGCTATGA--TGGAAA-----GGACACG-GTGCTGACAT--

TACTCTTCTACTACAGGTTCACCTCCAGACCGGCCAGTGCGTAAGTAGAATCGATTC--GAGC----CCGACGACC-GATACGGTGTG-
GTGGTGGTGCTACTATGGGCGAAGACTGACAACCAGCT-CTCCAGGGTAACCAAATCGGTGCCGCTTTCTGGTATGTTTTG--
ATGCCAACTCCATCCGT-----
TTGCGTGTCTATGGGCAATGAAGACTGACTTCTATCACAGGCAGACCATCTCTGGCGAGCACGGCCTCGACAGCAATGGCGTGTACGTGA
CTGTCGCCGACCCAAACCTCCGATAA--TCCC---TCGCTCACCGCTAT-
GATAGGTACAACGGCACCTCGGAGCTCCAGCTCGAGCGCATGAACGTCTACTTCAACGAGGTGAGTCGCGCTCATACACC-
GTCATATGTTGCTT----TCGAGGCTGC-GGTCGCTGACAATATA----
TGTTTTTCAGGCTTCCGGCAACAAGTATGTCCCTCGTGCCGTCCTCGTCGACTTGGAGCCCGGCACCATGGATGCCGTCCGCGCTGGTCCC
TTCGGCCAGCTCTTCCGCACCGACAACTT

>Amesia_nigricolor_CBS_291_83

ATTACAGAGTTGC-AAAACTCCCTAAACCATTGTGAACGTTACCTTT-AACCGTTGCTTCGGCGGGCGGCGCTCT----------------------------
GCCGCCCCTCGGCCCTGC---------------------CCGGGCGCCCGCCGGAGGATACCC-AACTCTTGATACTTT-
GATGGCCTCTCTGAGTCTTCTGTACTGAA-
TAAGTCAAAACTTTCAACAACGGATCTCTTGGTTCTGGCATCGATGAAGAACGCAGCGAAATGCGATAAGTAATGTGAATTGCAGAATTC
AGTGAATCATCGAATCTTTGAACGCACATTGCGCCCGCCAGTATTCTGGCGGGCATGCCTGTTCGAGCGTCATTTCAACCATCAAGCCCAC
-GGCTTGTGTTGGGGACCTGCG----------GCTGCCCGCAGGCCCTGAAAACCAGTGGCGGGCTCGCT-GTCACACCGAGCGTAGTAG--
CATCATTCTCGCTCAGGGCG-TGCTGCGGGTT--CCGGCCGTTAAA-
CGACCTTCATAACCCAAGGTTGACCTCGGATCAGGTAGGAAGACCCGCTGAACTTAAGCATATCAATAAGCGGAGGAAAAGAAACCAA
CAGGGATTGCCC-TAGTAACGGCGAAGTGAAGCGGCAACAGCTCAAATTTGAAATCTGGCTTCGGCCCGAGTTGTAATTTGCAG-
AGGAAGCTTTAGGCGCGGCACCTTCTGAGTCCCCTGGAACGGGGCGCCATAGAGGGTGAGAGCCCCGTATAGTTGGATGCCTAGCCTGT
GTAAAGCTCC-
TTCGACGAGTCGAGTAGTTTGGGAATGCTGCTCAAAATGGGAGGTAAATTTCTTCTAAAGCTAAATACCGGCCAGAGACCGATAGCGCA
CAAGTAGAGTGATCGAAAGATGAAAAGCACTTTGAAAAGAGGGTTAAATAGCACGTGAAATTGTTGAAAGGGAAGCGCTTGTGACCAG
ACTTGCGCCGGGCTGATCATCCGGTGTTCTCACCGGTGCACTCTGCCCGGCTCAGGCCAGCATCGGTTCTCGCGGGGGGATAAAGGTTCC
GGGAATGTAGCTCCTCCGGGAGTGTTATAGCCCGGGGCGTAATGCCCTCGCGGGGACCGAGGTTCGCGC-
TCTGCAAGGATGCTGGCGTAATGGTCATCAGCGACCCGTCTTGAAACACGGACCAAGGAGTCAAGGTTTTGCGCGAGTGTTTGGGTGTAA
AACCCGCACGCGTAATGAAAGTGAACGTAGGTGAGAGCTTCGGCGCATCATCGACCGATCCTGATGTTTTCGGATGGATTTGAGTAAGAG

CGTTAAGCCTTGGACCCGAAAGATGGTGAACTATGCTTGGATAGGGTGAAGCCAGAGGAAACTCTGGTGGAGGCTCGCAGCGG-TTCTGACGTGCAAATCGATCGTCAAATCTGAGCATCTGGTCACATTGGTGCAAGGTCTGCGGAGGAAGAACGTCATTTCGTTCGAGGTGTCGTTGGTTCGCGACATCCGCGACCGCGAGTTCAAGATCTTTTCCGATGCGGGGCGCGTCATGAGGCCGCTATTTACCGTCGAGCAGGAGCACGGTTCAGAGACCGGCGCCGAAATGGGGCAGCTCATCCTCAACAAGGAACATATTACACGGTTAGAAGCGGACAAGGAGCTGGGCAAATACCATCCCGACTACTGGGGCTGGCAGGGCCTGCTTAAGTCGGGCGCCATCGAGTATCTCGATGCCGAGGAGGAGGAGACGGCCATGATCTGCATGACGCCCGAGGACCTCGACAAGTTCCGGTACAGAAAGATGGGGTTCATCGTCGAAGACAACTCGGGTCAAGGTAACAACAGGATCAAGACGAAGCCGAACCCCGCCACCCACATGTACACGCACTGCGAGATCCATCCCAGCATGTTGCTCGGCATCTGCGCCAGCATCATCCCCTTCCCCGACCACAACCAGACGCGTCGGGAAGCT---TTTTT-TGGGCCCCTGAT---CTACCT--CACATATTGGGA-CGACCTCGTT---TGACCAGCT--ACCCGA----CCGACC----GAGAGCGATGACGGCGCGGCCGCGA--TGCAAA-----GGACATG-GTGCTGACAT--TGCTTCTCTACTATAGGTTCACCTCCAGACCGGCCAATGCGTAAGTTGGATCGATTC--GAGC----CCGACGACC-GATACCATG----TGCCAGTGC------GGGTGTAGACTGACAACAAGCT-CTCCAGGGTAACCAAATTGGTGCTGCTTTCTGGTATG-TTCG--ATGCCAACATCAACCGC-----GTGCGTGTCAA-AAGCAATCGAGACTGACTTCCATCACAGGCAGACCATCTCTGGCGAGCACGGCCTCGACAGCAATGGCGTGTACGTGACTGTCGGCGACCCAAA-TTCCGATAA--TCGC---TCGCTCACCGGTAC-GATAGGTACAACGGCACCTCGGAGCTCCAGCTCGAGCGCATGAACGTCTACTTCAACGAGGTGAGTTGGGCCTGTACACC-TTCATCAGTTATCT----TGAAGGCTGT-GGTTGCTGACAACATA----TTCTTCGCAGGCTTCCGGCAACAAGTATGTCCCTCGTGCCGTCCTCGTCGACTTGGAGCCCGGCACCATGGATGCCGTCCGCGCTGGTCCCTTCGGCCAGCTCTTTCGCCCTGACAACTT

>Amesia_nigricolor_CBS_600_66

ATTACAGAGTTGC-AAAACTCCCTAAACCATTGTGAACGTTACCTTT-AACCGTTGCTTCGGCGGGCGGCGCTCT---------------------------GCCGCCCCTCGGCCCTGC---------------------CCGGGCGCCCGCCGGAGGATACCC-AACTCTTGATACTTT-GATGGCCTCTCTGAGTCTTCTGTACTGAA-TAAGTCAAAACTTTCAACAACGGATCTCTTGGTTCTGGCATCGATGAAGAACGCAGCGAAATGCGATAAGTAATGTGAATTGCAGAATTCAGTGAATCATCGAATCTTTGAACGCACATTGCGCCCGCCAGTATTCTGGCGGGCATGCCTGTTCGAGCGTCATTTCAACCATCAAGCCCAC-GGCTTGTGTTGGGGACCTGCG----------GCTGCCCGCAGGCCCTGAAAACCAGTGGCGGGCTCGCT-GTCACACCGAGCGTAGTAG--CATCATTCTCGCTCAGGGCG-TGCTGCGGGTT--CCGGCCGTTAAA-CGACCTTCATAACCCAAGGTTGACCTCGGATCAGGTAGGAAGACCCGCTGAACTTAAGCATATCAATAAGCGGAGGAAAAGAAACCAA

CAGGGATTGCCC-TAGTAACGGCG-AGTGAAGCGGCAACAGCTCAAATTTGAAATCTGGCTTCGGCCCGAGTTGTAATTTGCAG-
AGGAAGCTTTAGGCGCGGCACCTTCTGAGTCCCCTGGAACGGGGCGCCATAGAGGGTGAGAGCCCCGTATAGTTGGATGCCTAGCCTGT
GTAAAGCTCC-
TTCGACGAGTCGAGTAGTTTGGGAATGCTGCTCAAAATGGGAGGTAAATTTCTTCTAAAGCTAAATACCGGCCAGAGACCGATAGCGCA
CAAGTAGAGTGATCGAAAGATGAAAAGCACTTTGAAAAGAGGGTTAAATAGCACGTGAAATTGTTGAAAGGGAAGCGCTTGTGACCAG
ACTTGCGCCGGGCTGATCATCCGGTGTTCTCACCGGTGCACTCTGCCCGGCTCAGGCCAGCATCGGTTCTCGCGGGGGGATAAAGGTTCC
GGGAATGTAGCTCCTCCGGGAGTGTTATAGCCCGGGGCGTAATGCCCTCGCGGGGACCGAGGTTCGCGC-
TCTGCAAGGATGCTGGCGTAATGGTCATCAGCGACCCGTCTTGAAACACGGACCAAGGAGTCAAGGTTTTGCGCGAGTGTTTGGGTGTAA
AACCCGCACGCGTAATGAAAGTGAACGTAGGTGAGAGCTTCGGCGCATCATCGACCGATCCTGATGTTTTCGGATGGATTTGAGTAAGAG
CGTTAAGCCTTGGACCCGAAAGATGGTGAACTATGCTTGGATAGGGTGAAGCCAGAGGAAACTCTGGTGGAGGCTCGCAGCGG-
TTCTGACGTGCAAATCGATCGTCAAATCTGAGCATCTGGTCACATTGGTGCAAGGTCTGCGGAGGAAGAACGTCATTTCGTTCGAGGTGT
CGTTGGTTCGCGACATCCGCGACCGCGAGTTCAAGATCTTTTCCGATGCGGGGCGCGTCATGAGGCCGCTATTTACCGTCGAGCAGGAGC
ACGGTTCAGAGACCGGCGCCGAAATGGGGCAGCTCATCCTCAACAAGGAACATATTACACGGTTAGAAGCGGACAAGGAGCTGGGCAA
ATACCATCCCGACTACTGGGGCTGGCAGGGCCTGCTTAAGTCGGGCGCCATCGAGTATCTCGATGCCGAGGAGGAGGAGACGGCCATGA
TCTGCATGACGCCCGAGGACCTCGACAAGTTCCGGTACAGAAAGATGGGGTTCATCGTCGAAGACAACTCGGGTCAAGGTAACAACAGG
ATCAAGACGAAGCCGAACCCTGCCACCCACATGTACACGCACTGCGAGATCCATCCCAGCATGCTGCTCGGCATCTGCGCCAGCATCAT
CCCCTTCCCCGACCACAACCAGACGCGTCGAGAAGCT---TTTTT-TGGGCCCCTGAT---CTACCC--CACATATTGGGA-CGACCTCGTT---
TGACCAGCT--ACCCGA----CCGACC----GAGAGCGATGACGGCGCGGCCGCGA--TGCAAA-----GGACATG-GTGCTGACAT--
TGCTTCTCTACTATAGGTTCACCTCCAGACCGGCCAATGCGTAAGTTGGATCGATTC--GAGC----CCGACGACC-GATACCATG----
TGCCAGTGC------AGGTGTAGACTGACAACAAGCT-CTCCAGGGTAACCAAATCGGTGCTGCTTTCTGGTATG-TTCG--
ATGCCAACATCAACCGC-----GTGCGTGTCAA-
AAGCAATCGAGACTGACTTCCATCACAGGCAGACCATCTCTGGCGAGCACGGCCTCGACAGCAATGGCGTGTACGTGACTGTCGGCGAC
CCAAA-TTCCGATAA--TCGC---TCGCTCACCGGTAC-
GATAGGTACAACGGCACCTCGGAGCTCCAGCTCGAGCGCATGAACGTCTACTTCAACGAGGTGAGTTGGGCCTGTCCACC-
TTCATCAGTTATCT----TGAAGGCTGC-GGTTGCTGACAACATA----
TTCTTCGCAGGCTTCCGGCAACAAGTATGTCCCTCGTGCCGTCCTCGTCGACTTGGAGCCCGGCACCATGGATGCCGTCCGCGCTGGTCCC
TTCGGCCAGCTCTTTCGCCCTGACAACTT

>Humicola_fuscoatra_CBS_118_14

ATTACAGAGTTGC-AAAACTCCC-AAACCATTGTGAACAT-ACC-TA-CCCCGTTGCTTCGGCGGGCGG-CCCGGGCCC-CGCGCCC--------------
-GGCGCCCCCCGGCCCCCC----------------------GTGGGCGCCCGCCGGAGGTAAAACAAACCCTTGAAT-TGC--
ATGGCCTCTCTGAGTCTTCTGTACTGAA-
TAAGTCAAAACTTTCAACAACGGATCTCTTGGTTCTGGCATCGATGAAGAACGCAGCGAAATGCGATAAGTAATGTGAATTGCAGAATTC
AGTGAATCATCGAATCTTTGAACGCACATTGCGCCCGCCAGTATTCTGGCGGGCATGCCTGTTCGAGCGTCATTTCAACCATCAAGCCCCC
-GGCTTGTGTTGGGGACCTGCG----------GCTG-CCGCAGGCCCTGAAAACCAGTGGCGGGCTCGCT-GTCACCCCGAGCGTAGTAG---
TTACATCTCGCTCTGGGAG-TGCTGCGGGTT--CCGGCCGTTAAA-CGCCTTA-
TTTACCCAAGGTTGACCTCGGATCAGGTAGGAAGACCCGCTGAACTTAAGCATATCAATAAGCGGAGGAAAAGAAACCAACAGGGATT
GCCC-CAGTAACGGCG-AGTGAAGCGGCAACAGCTCAAATTTGAAATCTGGCTTCGGCCCGAGTTGTAATTTGCAG-
AGGAAGCTTTAGGCGCGGCACCAACTGAGTCCCCTGGAACGGGGCGCCACAGAGGGTGAGAGCCCCGTATAGTTGGACGCCTAGCCTGT
GTAAAGCTCC-
TTCGACGAGTCGAGTAGTTTGGGAATGCTGCTCAAAATGGGAGGTAAATTTCTTCTAAAGCTAAATACCGGCCAGAGACCGATAGCGCA
CAAGTAGAGTGATCGAAAGATGAAAAGCACTTTGAAAAGAGGGTTAAATAGCACGTGAAATTGTTGAAAGGGAAGCGCTTGTGACCAG
ACTTGCGCCCGGCTGATCATCCGGTGTTCTCACCGGTGCACTCTGCCGGGCTCAGGCCAGCATCGGTTCTCGCGGGGGGATAAAGGTCCT
GGGAACGTAGCTCCTCCGGGAGTGTTATAGCCCAGGGCGTCATGCCCTCGCGGGGACCGAGGTTCGCGCATCTGCAAGGATGCTGGCGT
AATGGTCATCAGCGACCCGTCTTGAAACACGGACCAAGGAGTCAAGGTTTTGCGCGAGTGTTTGGGTGTAAAACCCGCACGCGTAATGA
AAGTGAACGTAGGTGAGAGCTTCGGCGCATCATCGACCGATCCTGATGTTTTCGGATGGATTTGAGTAGGAGCGTTAAGCCTTGGACCCG
AAAGATGGTGAACTATGCTTGGATAGGGTGAAGCCAGAGGAAACTCTGGTGGAGGCTCGCAGCGG-
TTCTGACGTGCAAATCGATCGTCAAATCTGAGCATCTGGTCACGCTGGTCCAGGGGCTGCGGAGAAAGAACGTCATTTCGTTCGAGGTGT
CGCTCGTCAGAGACATCCGCGACCGCGAGTTCAAGATTTTCTCGGATGCCGGCCGTGTCATGAGGCCGCTCTACACGGTAGAGCAAGAG
CCAAACAGCGACAGCGGCGCCGAAATCGGGCAGCTGATCCTGAACAAGGAGCACGTTACGCGGCTCGAGGCCGACAAAGAGCTGGGCA
AGTACCATCCCGACTACTGGGGCTGGCAAGGCCTCTTGAAGTCGGGTGCCATCGAGTACCTCGACGCCGAGGAGGAGGAGACGGTCATG
ATCTGCATGACCCCCGAAGACCTCGACAAGTTCCGGTACCGCAAGCTGGGGTTCATCGTGGAGGACAACTCTGGCCAAGGCAACAACAG
AATCAAGACGAGGCCGAACCCGACGACGCACATGTACACGCATTGCGAGATCCACCCCAGCATGCTGCTCGGCATCTGCGCGAGCATCA
TCCCGTTCCCCGACCACAACCAGGCGTCC-GAGAAGCT---TTTTT-TGGACCCCTGAT---CTACCC----CACATCGGGG-CGACGTCGTC---
CCACCAGCT--TCCCGAGGC-CCAACTC---GCAGGCGTTGGCGACGCGGCCACGACAACAAGAGACCCGGGCGCG-

ATGCTGACATTATGTTTTTCTGCTACAGGTTCACCTCCAGACCGGCCAGTGCGTAAGTTGCACCGACTC--GAAC---GCTGGCGACC-GATATACCGTG-------GTGC------GGGTG--GACTGACGTTGAAGC--TCTAGGGTAACCAAATTGGTGCCGCTTTCTGGTATG-ATCC--GACCCAACCTCAAGAGC-----CACGCTATCCA-TGGCAATCAATACTGACTTCCGCTCCAGGCAGACCATCTCCGGCGAGCACGGCCTCGATGGCAGTGGCGTGTACGTGCCTGTCGCCGCGTCCTCTCTCGATTAA--TCCCTCGTCGCTCACCGCTCC-CATAGGTACAATGGCACCTCCGAGCTCCAGCTCGAGCGCATGAACGTCTACTTCAACGAGGTCAGTCGGACTCATCTCCCTGTCGTGGGAATGTC----GAGCGACTCT-GGTTTCTGACAATGCATGTCAATTCTACAGGCTTCCGGCAACAAGTATGTTCCTCGTGCCGTCCTGGTCGACTTGGAGCCCGGCACCATGGATGCCGTCCGCGCTGGTCCCTTCGGCCAGCTCTTCCGCCCTGACAACTT

>Humicola_olivacea_DTO_319_C7

ATTACAGAGTTGC-AAAACTCCC-AAACCATTGTGAACGTTACCTTT--ACCGTTGCTTCGGCGGGCGG-CCCGGGTCC-TGCCC------------------GGCGCCCCTCGGCCCTCG-----------------------CGGGCGCCCGCCGGAGGTAAACCAAACTATTGCAT-TGT--ATGGCCTCTCTGAGTCTTCTGTACTGAA-TAAGTCAAAACTTTCAACAACGGATCTCTTGGTTCTGGCATCGATGAAGAACGCAGCGAAATGCGATAAGTAATGTGAATTGCAGAATTCAGTGAATCATCGAATCTTTGAACGCACATTGCGCCCGCCAGTATTCTGGCGGGCATGCCTGTTCGAGCGTCATTTCAACCATCAAGCCCCC-GGCTTGTGTTGGGGACCTGCG----------GCTG-CCGCAGGCCCTGAAAACCAGTGGCGGGCTCGCTAGTCACTCCGAGCGTAGTAG--TTTACATCTCGCTCAGGGCG-TGCTGCGGGTT--CCGGCCGTTAAA-AGCCTTATTTTACCCAAGGTTGACCTCGGATCAGGTAGGAAGACCCGCTGAACTTAAGCATATCAATAAGCGGAGGAAAAGAAACCAACAGGGATTGCCC-TAGTAACGGCG-AGTGAAGCGGCAACAGCTCAAATTTGAAATCTGGCTTCGGCCCGAGTTGTAATTTGCAG-AGGAAGCTTTAGGCGCGGCACCAACTGAGTCCCCTGGAACGGGGCGCCACAGAGGGTGAGAGCCCCGTATAGTTGGACGCCTAGCCTGTGTAAAGCTCC-TTCGACGAGTCGAGTAGTTTGGGAATGCTGCTCAAAATGGGAGGTAAATTTCTTCTAAAGCTAAATACCGGCCAGAGACCGATAGCGCACAAGTAGAGTGATCGAAAGATGAAAAGCACTTTGAAAAGAGGGTTAAATAGCACGTGAAATTGTTGAAAGGGAAGCGCTTGTGACCAGACTTGCGCCCGGCTGATCATCCGGTGTTCTCACCGGTGCACTCTGCCGGGCTCAGGCCAGCATCGGTTCTCGCGGGGGGGTAAAGGCCCTGGGAACGTAGCTCCTCCGGGAGTGTTATAGCCCAGGGCGTAATGCCCTCGCGGGGACCGAGGTTCGCGCATCTGCAAGGATG??CTGGTGACGC

TGGTCCAAGGGTTGCGGCGAAAGAACGTCATCTCCTTCGAGGTCTCGCTTGTCCGCGACATCCGCGATCGCGAGTTCAAGATCTTCTCCGA
CGCCGGCCGTGTGATGAGGCCACTGTACACGGTCGAGCAAGAGCAGAACAGCGACAGCGGTGCGGAAGTCGGACAGCTGATTCTGAAC
AAGGAGCATGTTACGCGCCTGGAGGCGGACAAGGAGCTGGGCAAGTACCATCCCGACTACTGGGGCTGGCAGGGCCTGCTCAAGTCGG
GTGCCATCGAGTACCTCGACGCCGAGGAGGAGGAAACCGTCATGATTTGCATGACGCCCGAGGACCTCGATAAATTTCGGTACCGCAAG
ATGGGCTTTATTGTCGAGGACAACTCTGGTCAAGGCAACAACAGGATCAAGACGAGGCCGAATCCGACAACTCACATGTACACGCATTG
CGAGATCCACCCCAGCATGCTGCTCGGTATCTGCGCGAGCATTATTCCCTTCCCCGACCACAACCAGGCGTCC-GAGAAGCTT--TTTCT-
AGGACCCCTGAT---CTACCC--CACATCGGGAAG-ACAGGTCGTTCCACCACCAGCTTGTCCCGA----
CCAGCTCCGGGCGGGCGATGGCGATGCCGCCGTGA-ATCACAAG----CGACGCG-ATACTGACAT--
TGTTCTCTCACTACAGGTTCACCTCCAGACCGGCCAGTGCGTAAGTTGAGCCGACGC--GAAGACGACTCTCGATC-GATCAGGTGG--------
TTGC------AGGTG--GACTGACGGTGGCCT--TCCAGGGTAACCAAATCGGTGCCGCTTTCTGGTATG-TTCA--
GCCTCGAGCATCAAGACGTGTGTTTTGTATCGACGGCATCAGAATACTGACTTTCTATCCAGGCAGACCATCTCCGGCGAGCACGGCCTC
GACGGCAATGGCGTGTACGTGACCGTCGCCGCCCCGCGTCTCGATTAA--TCAC---TCGCTCACCGCCAC-
CACAGGTACAATGGCAGCTCCGAGCTCCAGCTCGAGCGCATGAACGTCTACTTCAACGAGGTCAGTCGTGCTCGCCAGAA---CGTTAA-----
-----TGTGGTCCGG-GGTTGCTGACAAGTGATGT-
TTTGCTGCAGGCCTCGGGCAACAAGTATGTCCCCCGTGCCGTCCTCGTCGACTTGGAGCCCGGCACCATGGATGCCGTCCGCGCCGGTCC
CTTCGGCCAGCTCTTCCGCCCCGACAACTT

>Hyalosphaerella_fragilis_CBS_456_73

ATTACAGAGTTGC-AAAACTCCCTAAACCATTGTGAACCTTACCTTTCAACCGTTGCTTCGGCGGGCGGGCCC------------------------------
CGTGCCCCTGGGCCCCTC---------------------G-CGGGCGCCCGCCGGAGGTCACCCAAACTCTTGATACTTT--
ATGGCCTCTCTGAGTCTTCTGTACCGAA-
TAAGTCAAAACTTTCAACAACGGATCTCTTGGTTCTGGCATCGATGAAGAACGCAGCGAAATGCGATAAGTAATGTGAATTGCAGAATTC
AGTGAATCATCGAATCTTTGAACGCACATTGCGCCCGCCAGTATTCTGGCGGGCATGCCTGTTCGAGCGTCATTTCAACCATCAAGCCCCG
-GGCTTGTGTTGGGGACCTGCG----------GCTGCCCGCAGGCCCTGAAAACCAGTGGCGGGCTCGCT-GTCACACCGAGCGTAGTAG--CATCA-
CCTCGCTCAGGGCG-TGCTCCGGGTT--CCGGCCGTTAAA-
AGCCTTTCCAAACCCAAGGTTGACCTCGGATCAGGTAGGAAGACCCGCTGAACTTAAGCATATCAATAAGCGGAGGAAAAGAAACCAA
CAGGGATTGCCT-TAGTAACGGCGAAGTGAAGCGGCAACAGCTCAAATTTGAAATCTGGCTTCGGCCCGAGTTGTAATTTGCAG-
AGGAAGCTTTAGGCGCGGCACCTTCTGAGTCCCCTGGAACGGGGCGCCATAGAGGGTGAGAGCCCCGTATAGTTGGATGCCTAGCCTGT

GTAAAGCTCC-
TTCGACGAGTCGAGTAGTTTGGGAATGCTGCTCAAAATGGGAGGTAAATTTCTTCTAAAGCTAAATACCGGCCAGAGACCGATAGCGCA
CAAGTAGAGTGATCGAAAGATGAAAAGCACTTTGAAAAGAGGGTTAAATAGCACGTGAAATTGTTGAAAGGGAAGCGCTTGTGACCAG
ACTTGCGCCGGGCTGATCATCCGGTGTTCTCACCGGTGCACTCTGCCCGGCTCAGGCCAGCATCGGTTCTCGCGGGGGATAAAGGCCCT
GGGAACGTAGCTCCTCCGGGAGTGTTATAGCCCGGGGCGCAATGCCCTCGCGGGGACCGAGGTTCGCGC-
TCTGCAAGGATG???
??
??????????????CTGGTCACCCTGGTCCAAGGGTTGCGGCGGAAGAATGTCATTTCGTTTGAGGTGTCGCTCGTCAGGGACATCCGCGACCGC
GAGTTCAAGATCTTCTCGGATGCTGGCCGTGTCATGAGGCCACTGTTCACCGTGGAGCAAGAGCACAACAGCGAGAGCGGCGCCGAGGT
GGGCCAGCTGATTCTCAACAAGGAACACATCAACCGACTGGAGACGGACAAGGAGTTGGGCAAATACCATCCCGATTACTGGGGTTGGC
AGGGCTTGTTAAAGTCGGGTGCCATCGAGTACCTGGACGCCGAGGAGGAGGAGACGGTCATGATCTGCATGACTCCCGAGGACCTCGAC
AAGTTCCGTTACCGCAAAATGGGCTTCATCATTGAAGACAATTCTGGTCAGGGTAACAACAGGATCAAGACGAAGCCGAACCCGACGAC
TCACATGTACACCCACTGCGAGATCCACCCCAGCATGTTGCTCGGCATCTGCGCGAGCATCATTCCGTTCCCAGACCACAACCAGGCGTC
T-GGGAAGCTT--TTTTT-TGGGCCCCTGAT---CTACCCCACACACATCGAGA-CGACGTCGTC---CCACCAGCT---CCCGA----CCGACC----
GATGGCGATGGTGGCGCTGCCATGA--TGGGAA-----GTACACG-ATGCTGACAT--
TGTCTCTTTGCTACAGGTTCACCTTCAGACCGGCCAGTGCGTAAGTTGAACCGATTC--GAAC----ATGGTGACC-GATCACATG---------GTGC-
-----GGGTG--GACTGACATGAAGCT-CCCCAGGGTAACCAAATCGGTGCCGCTTTCTGGTATG-TTAA-TTCGAAAACACCAAGTCC-----
GTTGTAACCCATGGGCCATCAATGCTGACTTCCTTCACAGGCAGACCATCTCTGGCGAGCACGGCCTCGACAGCAATGGCGTGTACGTGA
CTGTCGCCGATTCCGA--TCAATTAA--GCCC---TCGCTCACCGCTAC-
AATAGGTACAATGGCACCTCCGAGCTCCAGCTCGAGCGCATGAACGTCTACTTCAACGAGGTAAGTCGGCCTCACACACCTTTACTGTGT
CACGAC---AATCATTTGC-GTTTGCTGACGAATGA----
ATCTCTCCAGGCTTCCGGCAACAAGTATGTCCCTCGTGCCGTCCTGGTCGACCTGGAGCCCGGCACCATGGATGCCGTCCGCGCTGGTCCC
TTCGGTCAGCTCTTCCGCCCTGACAACTT

>Parathielavia_kuwaitensis_CBS_945_72

?????????????????????????????????????C--TCAACCGTTGCTTCGGCGGGCGGCCCGGGCCTCACCGCCC---------------
GGACGCCCCCCCAGGCCCC-TC------------------GCGGGGCGCCCGCCGGAGGTA-CCCAAACTCTTGAATATAT--
ATGGCCTCTCTGAGTCTTCTGTACTGAA-

TCAGTCAAAACTTTCAACAACGGATCTCTTGGTTCTGGCATCGATGAAGAACGCAGCGAAATGCGATAAGTAATGTGAATTGCAGAATTC
AGTGAATCATCGAATCTTTGAACGCACATTGCGCCCGCCAGTATTCTGGCGGGCATGCCTGTCCGAGCGTCATTTCAACCATCAAGCCCC
G-CGCTTGTGTTGGGGTCCTGCG----------GCTGCCCGCAGGCCCTGAAAACCAGTGGCGGGCTCGCT-GTCGCACCGAGCGTAGTAG--
CATAA-
CCTCGCTCAG??GCATATCAATAAGCGGAGGAAAAGA
AACCAACAGGGATTGCCC-CAGTAACGGCG-AGTGAAGCGGCAACAGCTCAAATTTGAAATCTGGCCTCGGCCCGAGTTGTAATTTGCAG-
AGGAAGCTTTAGGCGCGGCCCCAACTGAGTCCCCTGGAACGGGGCGCCACAGAGGGTGAGAGCCCCGTATAGTTGGACGCCTAGCCTGT
GTAAAGCTCC-
TTCGACGAGTCGAGTAGTTTGGGAATGCTGCTCAAAATGGGAGGTAAATTTCTTCTAAAGCTAAATACCGGCCAGAGACCGATAGCGCA
CAAGTAGAGTGATCGAAAGATGAAAAGCACTTTGAAAAGAGGGTTAAATAGCACGTGAAATTGTTGAAAGGGAAGCGCTTGTGACCAG
ACTTGCGCCGGGCTGATCATCCGGTGTTCTCACCGGTGCACTCTGCCCGGCTCAGGCCAGCATCGGTTCTCGCGGGGGGACAAAGGCTCC
GGGAACGTAGCTCCTCCGGGAGTGTTATAGCCCGGGGCGCAATGCCCCCGCGGGGACCGAGGACCGCGC-
TCTGCAAGGATGCTGGCGTAATGGTCATCAGCGACCCGTCTTGAAACACGGACCAAGGAGTCAAGGTTTTGCGCGAGTGTTTGGGTGTAA
AACCCGCACGCGTAATGAAAGTGAACGTAGGTGAGAGCTTCGGCGCATCATCGACCGATCCTGATGTTCTCGGATGGATTTGAGTAGGA
GCGTTAAGCCTTGGACCCGAAAGATGGTGAACTATGCTTGGATAGGGTGAAGCCAGAGGAAACTCTGGTGGAGGCTCGCAGCGG-
TTCTGACGTGCAAATCGATCGTCAAATCTGAGCATCTGGTCAGCTTGGTTCAGGGATTGCGGCGAAAGAACGTCATCTCGTTCGAGGTTTC
CCTTGTCAGGGACATCCGCGACCGCGAGTTCAAGATCTTCTCGGATGCCGGCCGTGTCATGAGGCCGCTCTACACCGTCGAGCAAGAGCC
AAACAGCGAGACCGGGGCCGAGGTGGGGCAGCTGATTCTCAACAAGGAACACATCAGCCGGTTGGAAGCGGACAAGGAGCTGGGCAA
GTACCATCCCGATTACTGGGGTTGGCAGGGGCTGCTCAAGTCGGGTGCCATCGAGTACCTGGATGCCGAGGAGGAAGAGACAGTCATGA
TCTGCATGACGCCCGAAGACCTCGACAAGTTCCGTTACCGCAAGATGGGCTTCATCATCGAAGACAATTCTGGTCAGGGTAACAACAGG
ATCAAGACGAAGCCGAACCCGACGACCCACATGTATACCCACTGCGAGATCCATCCCAGCATGCTGCTCGGCATCTGCGCGAGCATCAT
CCCCTTCCCCGACCACAACCAGGCGTCT-GGGTAGCAT--TTTTT-TGGGCCCCTGAT----TACCC--CACACATTGAGA-CGACGTCGTC---
CCACCCGTT---CCCGA----CCGACC----GCTGGCGATGGCGGCGCTGCCGTGA--TGGAAA-----GCACGCG-ATGCTGACTT--
TGTTTCTTTGCTACAGGTTCACCTCCAGACCGGCCAGTGCGTAAGTAGAATCGACTC--GGACCCGACCGACAACC-CATCAGATG---------
GTGC----TTGGGTG--GACTGACAGCGAGCT-C-CCAGGGTAACCAAATCGGTGCCGCCTTCTGGTAGG-TCAACCTCGAAAACAGCCAATCC-
----TTCATAACTCA-
AGGTGATTGATGCTGACGTCCTCGATAGGCAGACCATCTCTGGCGAGCACGGCCTCGACAGCAATGGCGTGTACGTGACTACCACC-
ATCCCGA-TCGATGTAC--TCCC---TCGCTCACCGCTAC-

AATAGGTACAACGGAACCTCCGAGCTCCAGCTCGAGCGCATGAACGTCTACTTCAACGAGGTTAGTCGACGTTATATACCTGCGCGATAA
TACAGCGAGGAGCAGCCGT-GTTTGCTGACGAGCGA-------
TCCTCAGGCTTCCGGCAACAAGTATGTCCCTCGTGCCGTCCTCGTCGACTTGGAGCCCGGCACCATGGACGCCGTCCGCGCTGGCCCCTTC
GGCCAGCTCTTCCGCCCTGACAACTT

>Subramaniula_anamorphosa_CBS_137114

ATTACAGAGTTAC-CCAACTCCC-AAACCATTGTGAACGTTACC--TCTCCCGTTGCTTCGGCGGGCGGG-CCGGCCCC-AGGGC------------------
CGCGCCCCCCCGGCCCCC-TC------------------GCGGGGCGCCCGCCGGAGGAAACCC-AACTCTTGATTATC---
ATGGCCTCTCTGAGTCTTCTGTACTGAA-
TAAGTCAAAACTTTCAACAACGGATCTCTTGGTTCTGGCATCGATGAAGAACGCAGCGAAATGCGATAAGTAATGTGAATTGCAGAATTC
AGTGAATCATCGAATCTTTGAACGCACATTGCGCCCGCCAGTATTCTGGCGGGCATGCCTGTTCGAGCGTCATTTCAACCATCAAGCCCCG
-TGCTTGTGTTGGGGACCTGCG----------GCTG-CCGCAGGCCCTGAAAACCAGTGGCGGGCTCGCT-GTCACACCGGGCGTAGTAA---
TTACATCTCGCTCAGGGCG-TGCTGCGGGTT--CCGGCCGTAAAA-
AGCCTTATTTTACCCAAGGTTGACC??GTGAAGCGGC
AACAGCTCAAATTTGAAATCTGGCTTCGGCCCGAGTTGTAATTTGCAG-
AGGAAGCTTTAGGCGCGGCACCATCTGAGTCCCCTGGAACGGGGCGCCACAGAGGGTGAGAGCCCCGTATAGATGGACGCCTAGCCTGT
GTAAAGCTCC-
TTCGACGAGTCGAGTAGTTTGGGAATGCTGCTCAAAATGGGAGGTAAATTTCTTCTAAAGCTAAATATTGGCCAGAGACCGATAGCGCAC
AAGTAGAGTGATCGAAAGATGAAAAGCACTTTGAAAAGAGGGTTAAATAGCACGTGAAATTGTTGAAAGGGAAGCGCTTGTGACCAGA
CTTGCGCCGGGCGGATCATCCGGTGTTCTCACCGGTGCACTCCGCCCGGCTCAGGCCAGCATCGGTTCTCGCGGGGGGATAAAGGCCCTG
GGAACGTAGCTCCTCCGGGAGTGTTATAGCCCAGGGTGTAATGCCCTCGCGGGGACCGAGGTTCGCGCATCTGCAAGGATGCTGGCGTA
ATGGTCATCAGCGACCCGTCTTGAAACACGGACCAAGGAGTCAAGGTTTTGCGCGAGTGTTTGGGTGTAAAACCCGCACGCGTAATGAA
AGTGAACGTAGGTGAGAGCTTCGGCGCATCATCGACCGATCCTGATGTTTTCGGATGGATTTGAGTAGGAGCGTTAAGCCTTGGACCCGA
AAGATGGTGAACTATGCTTGGATAGGGTGAAGCCAGAGGAAACTCTGGTGGAGGCTCGCAGCGG-
TTCTGACGTGCAAATCGATCGTCAAATCTGAGCATCTGGTCACGTTGGTGCAAGGGTTGCGCCGGAAGAACGTCATCTCGTTTGAGGTTTC
GCTTGTCCGCGACATTCGCGACCGCGAGTTCAAGATCTTCTCCGATGCGGGCCGCGTCATGAGGCCGCTGTTTACGGTGGAGCAGGAGCA
GAACGCGGAGAGCGGGGTTGAGGTCGGGCAGCTGATTCTCAACAAAGAGCACATCACGCGGCTAGAGGCGGACAAGGAGATAGGCCG
ATACCACCCCGACTACTGGGGTTGGCAGGGCTTGCTCAAGTCTGGTGCCATCGAGTATCTCGATGCCGAAGAGGAAGAGACCGTCATGAT

TTCCATGACGCCCGAGGATCTCGACAAGTTCCGGTACCGCAAGATGGGATTCGTCGTTGAGGACAACTCCGGCCAGGGTAACAACAGGA
TCAAGACAAGACCCAATCCGGCGACGCACATGTACAC??A
GCTT---TTTCC-TGGGCCCCTGAT---CTACCC--CACACACTGGGA-CAACCTCATTC--CCACGAGCT---ACCGA----CCGGTC-----
CGAGCGCGACAGGTGTAGGCATGA--TGAAAA-----GAGCACC-ATGCTAACAA--
TGTTCCCTATCTACAGGTCCACCTCCAGACCGGCCAGTGCGTAAGTTGAACCGATTT-GATAC----CGGGCGACC-GATCAGGCG---------
GTGC------GGGTG--AACTGACAGCGTGCCTCTC-AGGGTAACCAAATTGGTGCTGCTTTCTGGTATG-TTCA--GCCTCA-CGACAGACGT-----
CGACGTGCAAAAGGGCGACTGATACTGACT-
CCTCTCCAGGCAGACCATCTCTGGCGAGCACGGCCTCGACAGCAATGGCGTGTACGTGACCGTCGCCGATACCCG-CCCGACTGA--CCTC-
--TTACTCACCGCTTC-
GACAGGTACAACGGCACCTCGGAGCTGCAGCTCGAGCGCATGAACGTCTACTTCAACGAGGTGCGTTGGCTCTCTCCGCG--
TCATCGGCGACT-----AAGTGTTCCT-GGGTGCTGACAATCGC----
TATCATACAGGCGTCCGGCAACAAGTATGTTCCCCGCGCCGTCCTCGTCGACTTGGAGCCCGGCACCATGGACGCCGTCCGCGCTGGTCC
CTTCGGTCAGCTCTTCCGCCCCGACAACTT

>Subramaniula_cristata_CBS_156_52

ATTACAGAGTTTCTATAACTCCC-AAACCATTGTGAACCTTACC-TCAAACCGTTGCTTCGGCGGGCGGG-CCGGCCCC-AGGGC---------------
--CGCGCCCCCCAGGCCCC--TC------------------GCGGGGCGCCCGCCGGAGGAAACCC-AACTCTTGATTATT---
ATGGCCTCTCTGAGTCTTCTGTACTGAA-
TAAGTCAAAACTTTCAACAACGGATCTCTTGGTTCTGGCATCGATGAAGAACGCAGCGAAATGCGATAAGTAATGTGAATTGCAGAATTC
AGTGAATCATCGAATCTTTGAACGCACATTGCGCCCGCCAGTATTCTGGCGGGCATGCCTGTTCGAGCGTCATTTCAACCATCAAGCCCCC
-GGCTTGTGTTGGGGACCTGCG----------GCTG-CCGCAGGCCCTGAAAACCAGTGGCGGGCTCGCT-GTCACACCGAGCGTAGTAG-
ATTTTCATCTCGCTCAGGGCG-TGCTGCGGGTT--CCGGCCGTAAAA-AGCCTTA-
TTTACCCAAGGTTGACCTCGGATCAGGTAGGAAGACCCGCTGAACTTAAGCATATCAATAAGCGGAGGAAAAGAAACCAACAGGGATT
GCCC-CAGTAACGGCG-AGTGAAGCGGCAACAGCTCAAATTTGAAATCTGGCTTCGGCCCGAGTTGTAATTTGCAG-
AGGAAGCTTTAGGCGCGGCACCATCTGAGTCCCCTGGAACGGGGCGCCATAGAGGGTGAGAGCCCCGTATAGATGGACGCTAAGCCTGT
GTAAAGCTCC-
TTCGACGAGTCGAGTAGTTTGGGAATGCTGCTCAAAATGGGAGGTAAATTTCTTCTAAAGCTAAATATTGGCCAGAGACCGATAGCGCAC
AAGTAGAGTGATCGAAAGATGAAAAGCACTTTGAAAAGAGGGTTAAATAGCACGTGAAATTGTTGAAAGGGAAGCGCTTGTGACCAGA

CTTGCGCCTGGCTGATCATCCGGTGTTCTCACCGGTGCACTCTGCCAGGCTCAGGCCAGCATCGGTTCTCGCGGGGGATAAAGGCCCTG
GGAACGTAGCTCCTCCGGGAGTGTTATAGCCCAGGGTGCAATGCCCTCGCGGGGACCGAGGTTCGCGCATCTGCAAGGATG?????????????
???
???CTAGTCACATTA
GTCCAAGGGCTGCGGAGAAAGAATGTCATATCCTTCGAGGTGTCGCTCGTTCGCGACATCCGCGACCGCGAGTTCAAGATTTTCTCCGAT
GCTGGCCGCGTCATGAGGCCGCTGTTCACTGTAGAGCAAGAGCCCAACGCTGAGAGCGGTGCCGAGGTCGGCCAGTTGATTCTCAACAA
GGACCACATCGCGCGGTTGGAGGCGGATAAAGATCTAGGGAAGTATCACCCTGACTACTGGGGCTGGCAGGGCCTGCTCAAGTCGGGTG
CGATTGAGTACCTCGACGCTGAGGAGGAGGAAACGGTCATGATCTCCATGACGCCTGAGGATCTCGACAAGTTCCGGTACCGCAAGATG
GGCTTCGTCGTCGAGGACAACTCTGGCCAGGGCAACAACAGGATCAAGACCAAGCCGAATCCGACCACGCATATGTACACGCATTGCGA
GATCCACCCCAGCATGCTGCTGGGCATCTGCGCCAGTATCATTCCGTTCCCCGACCACAACCAGACGCGTCAGGAAGTT---TTTTC-
TGGGCCCCTGAT---TTACCC--CACACGCGAACGTCGACCTCATTT--CCACGAGCT--ACCCGA----CCGGTC-----
CAAGCGCGATAGACGTCGCCATGA--TGAAGA-----GAGTTTG-ATGCTGACAT--
CGCTTCTTAACTACAGGTCCACCTCCAGACCGGCCAGTGCGTAAGTTGAATCGACTC-GATAC----CGGACAACC-GATCAGGTG---------
GTGC------AGGTG--GACTGACAGCTCACGCCTT-AGGGTAACCAAATCGGTGCTGCTTTCTGGTATG-TTCG--ACTCCAACACCAACCAC-----
CGACAGGCACA-
AGGTGGCTGAGACTGACTTCTCCTCCAGGCAGACCATCTCCGGCGAGCACGGCCTTGACAGCAATGGCGTGTATGTGACCGTTGCCCATC
CCCG-GCCGACCGA--TCTC---CCGCTCACCGCTAC-
AACAGGTACAACGGCACGTCTGAGCTCCAGCTCGAGCGCATGAACGTCTACTTCAACGAGGTAGGTTGGCTTCACGCCTT---
CTTTGGGCACT-----CGGGTGGCGT-GGCTGCTGACGACAAC----
TGTTCTACAGGCTTCCGGCAACAAGTATGTTCCTCGCGCAGTCCTCGTCGACTTGGAGCCCGGCACCATGGACGCCGTCCGCGCCGGCCC
CTTCGGCCAGCTCTTCCGCCCCGACAACTT

>Subramaniula_thielavioides_CBS_122_78

ATTACAGAGTTTCTATAACTCCCCAAACCATTGTGAACCTTACC-TTCTACCGTTGCTTCGGCGGGCGGG-CCGGCCCC-AGGGC----------------
--CGCGCCCCCCAGGCCCCCTTC------------------GCGGGGCGCCCGCCGGAGGAAACCCAAACTCTTGATATCC---
ATGGCCTCTCTGAGTCTTCTGTACTGAA-
TAAGTCAAAACTTTCAACAACGGATCTCTTGGTTCTGGCATCGATGAAGAACGCAGCGAAATGCGATAAGTAATGTGAATTGCAGAATTC
AGTGAATCATCGAATCTTTGAACGCACATTGCGCCCGCCAGTATTCTGGCGGGCATGCCTGTTCGAGCGTCATTTCAACCATCAAGCCCCG

-GGCTTGTGCTGGGGACCTGCG----------GCTG-CCGCAGGCCCTGAAAACCAGTGGCGGGCTCGCT-GTCACACCGAGCGTAGTAG-
ATTCTCATCTCGCTCAGGGCG-TGCTGCGGGTT--
CCGGCCGTAAAACAGCCTTATTTACCCAAGGTTGACC??
????GTGAAGCGGCAACAGCTCAAATTTGAAATCTGGCTTCGGCCCGAGTTGTAATTTGCAG-
AGGAAGCTTTAGGCGCGGCACCATCTGAGTCCCCTGGAACGGGGCGCCATAGAGGGTGAGAGCCCCGTATAGATGGACGCTAAGCCTGT
GTAAAGCTCC-
TTCGACGAGTCGAGTAGTTTGGGAATGCTGCTCAAAATGGGAGGTAAATTTCTTCTAAAGCTAAATACCGGCCAGAGACCGATAGCGCA
CAAGTAGAGTGATCGAAAGATGAAAAGCACTTTGAAAAGAGGGTTAAATAGCACGTGAAATTGTTGAAAGGGAAGCGCTTGTGACCAG
ACTTGCGCCGGGCTGATCATCCGGTGTTCTCACCGGTGCACTCTGCCCGGCTCAGGCCAGCATCGGTTCTCGCGGGGGGATAAAGGCCCT
GGGAACGTAGCTCCTCCGGGAGTGTTATAGCCCAGGGTGTAATGCCCTCGCGGGGACCGAGGTTCGCGCATCTGCAAGGATGCTGGCGT
AATGGTCATCAGCGACCCGTCTTGAAACACGGACCAAGGAGTCAAGGTTTTGCGCGAGTGTTTGGGTGTAAAACCCGCACGCGTAATGA
AAGTGAACGTAGGTGAGAGCTTCGGCGCATCATCGACCGATCCTGATGTTTTCGGATGGATTTGAGTAGGAGCGTTAAGCCTTGGACCCG
AAAGATGGTGAACTATGCTTGGATAGGGTGAAGCCAGAGGAAACTCTGGTGGAGGCTCGCAGCGG-
TTCTGACGTGCAAATCGATCGTCAAATCTGAGCATCTGGTCACGCTGGTCCAGGGGCTGCGCAGGAAGAACGTCATCTCATTCGAGGTGT
CGCTGGTCCGCGACATTCGCGACCGCGAGTTCAAGATCTTCTCCGATGCGGGCCGTGTCATGAGGCCGCTCTTCACTGTGGAGCAAGAGC
ATAACGCGGAGAGCGGCGTCGAGGTCGGCCAGTTGATCCTCAACAAAGAGCACATCGCGCGGCTGGAGGCGGACAAGGAGCTGGGCAA
GTACCACCCGGATTACTGGGGCTGGCAGGGTCTGCTCAAGTCGGGTGCCATTGAGTACCTCGACGCCGAGGAGGAAGAAACGGTCATGA
TTTCCATGACGCCCGAGGATCTCGACAAGTTCCGGTACCGCAAGATGGGCTTCGTCGTCGAGGACAATTCCGGCCA????????????????????????
???ACGCGTCAGGGAAGC---TTTTT-
TGGGCCCCTGAT---CTACCC--CACGCGCGAAGA-CGACCTCACC---CCATGAGCC--TCCCGA----CCAGCC-----
CAAACGCGACAGATGTCGCCGTTG--TGGAA------GAGTATG-ATGCTGACGC--
TACTTCTCAACTATAGGTCCACCTCCAGACCGGCCAGTGCGTAAGTAGAATCGGTTCAGAAAC----CCGGCGACC-GACCAGGTG---------
GTGC------AGGTG--GACTGACAGCAAG----TTCAGGGTAACCAAATCGGTGCTGCTTTCTGGTAAG-TTCG--GCGTCAACAACAAGCAC-----
CGACACGCAGAAAAACAATCGAGACTGACCT-
TCATCCAGGCAAACCATCTCTGGCGAGCACGGCCTCGACAGCAATGGCGTGTACGTGACCAACGCTGACCCTGG--CCGATCGA--ACCA---
CCGCTCACCGCTTG-
AATAGGTACAATGGCACTTCGGAGCTCCAGCTCGAGCGCATGAACGTTTACTTCAACGAGGTGGGTTGGCTTGACGCCCC---
ATTCGACCATCT----GGGTGGGCGT-GGTTTCTGACAGCAGC----

TGCTCTACAGGCCTCCGGCAACAAGTATGTTCCTCGCGCCGTCCTCGTCGACTTGGAGCCCGGCACCATGGATGCCGTCCGTGCCGGTCCT
TTCGGCCAGCTCTTCCGCCCCGACAACTT

>Thielavia_hyrcaniae_CBS_353_62

???TTCAACCGTTGCTTCGGCGGGCGGGCCCGGGTCTCACCGCCC---------------
GGCCGCCCCACCGGCCCCGCTC-----------------GCGGGGCGCCCGCCGGAGGTACCCAAAACTCTTGATACTTT--
ATGGCCTCTCTGAGTCTTCTGTACTGAA-
TCAGTCAAAACTTTCAACAACGGATCTCTTGGTTCTGGCATCGATGAAGAACGCAGCGAAATGCGATAAGTAATGTGAATTGCAGAATTC
AGTGAATCATCGAATCTTTGAACGCACATTGCGCCCGCCAGTATTCTGGCGGGCATGCCTGTTCGAGCGTCATTTCAACCATCAAGCCCCG
-TGCTTGTGTTGGGGACCTGCG----------GCTGCCCGCAGGCCCTGAAAACCAGTGGCGGGCTCGCT-GTCACACCGAGCGTAGTAG--
CAACAACCTCGCTCAG??
?????????????GTTCACGGCGTAGTGAAGCGGCAACAGCTCAAATTTGAAATCTGGCTTCGGCCCGAGTTGTAATTTGCAG-
AGGAAGCTTTAGGTGCGGCCCCAACTGAGTCCCCTGGAACGGGGCGCCACAGAGGGTGAGAGCCCCGTATAGTTGGACGCCTAGCCTGT
GTAAAGCTCC-
TTCGACGAGTCGAGTAGTTTGGGAATGCTGCTCAAAATGGGAGGTAAATTTCTTCTAAAGCTAAATACCGGCCAGAGACCGATAGCGCA
CAAGTAGAGTGATCGAAAGATGAAAAGCACTTTGAAAAGAGGGTTAAATAGCACGTGAAATTGTTGAAAGGGAAGCGCTTGTGACCAG
ACTTGCGCCGGGCTGATCATCCGGTGTTCTCACCGGTGCACTCTGCCCGGCTCAGGCCAGCATCGGTTCTCGCGGGGGGATAAAGGCTCC
GGGAACGTAGCTCCTCCGGGAGTGTTATAGCCCGGGGCGTAATGCCCTCGCGGGGACCGAGGACCGCGC-
TCT??
???
?????????CTGGTCAGTTTGGTTCAAGGATTGCGGCGAAAGAACGTCATCTCGTTTGAGGTTTCGCTTGTCAGGGACATCCGCGACCGCGAGT
TCAAGATCTTCTCGGATGCTGGCCGTGTCATGAGGCCGCTGTTCACTGTCGAGCAAGAGCCAAACAGCGAGACCGGGGCTGAGGTGGGC
CAGCTGATTCTCAACAAGGAACACATTAGCCGGTTGGAAGCCGACAAGGAGCTGGGCAAATATCATCCCGATTACTGGGGTTGGCAGGG
CTTGTTGAAGTCGGGTGCCATCGAGTACCTGGATGCCGAGGAGGAGGAGACAGTCATGATTTGCATGACACCCGAGGACCTCGACAAGT
TCCGTTACCGCAAGATGGGCTTCATCATCGAAGACAATTCTGGTCAGGGTAACAACAGGATCAAGACGAAGCCGAACCCGACTACTCAC
ATGTACACCCACTGCGAGATCCATCCCAGCATGCTGCTCGGCGTTTGCGCGAGCATCATCCCGTTCCCAGACCACAACCAGGCGTCT-
GGCTAGCT---TTTTT-TGGGCCCCTAATTACCTACCC--CACACATCGAGA-CGACGTCGTC---CGACCCGCT---CCCGA----CCGACC----
GCTGGCGATGGCAGCACTGCCGTGA--TGGAAG-----GCACACA-ATGCTGACTA--

TGTTTCTTTGCTACAGGTTCACCTCCAGACCGGCCAGTGCGTAAGTAGAATCGAATC--GGAC----TCGGCATCC-CATCGGATG---------GTAC----TTGGGTG--GACTAACAAGAAGCT-C-CCAGGGTAACCAAATCGGGGCCGCCTTCTGGTAAG-TTGACCTCGAAAACAACGAATCC-----TTCATAAGTCA-AGGCGATCAATGCTGACATCTTTGATAGGCAGACAATCTCTGGCGAGCACGGTCTCGACAGCAATGGCGTGTACGTGACTGTCGCCGATCCTGA-TCGATTTAC--CTCC---TTGCTCACCGCTCC-GACAGGTACAATGGAACCTCCGAGCTTCAGCTCGAGCGCATGAACGTCTACTTCAACGAGGTACGTCGACCTCATACCACTTTACTGTAATACAACGAGGAGCACCCTT-GTTTGCTGACGAGCGA-------ACTTCAGGCTTCTGGCAACAAGTATGTCCCTCGTGCCGTCCTGGTCGACCTGGAGCCCGGCACCATGGATGCCGTCCGCGCCGGTCCCTTCGGTCAGCTCTTCCGCCCTGACAACTT

>Corynascella_humicola_CBS_337_72

ATTACAGAGTTGA-AAGACTCCCCATACCATCGCGAACGTTACCCTT-AACCGTTGCTTCGGCGGGCGGTCTCCTCTCCCCCCCCCTTCTGGGGGCGGGTTGGGAAGCCCCTCGGCCCTCCCTTTCCCTTCGCGGGGTTGGGGCGGGGCGCCCGCCGGAGGTCACGCAAACTCTTGATACCTGAAACGGCCTCTCTGAGTATTCTGTACTTAATTAAGTCAAAACTTTCAACAACGGATCTCTTGGTTCTGGCATCGATGAAGAACGCAGCGAAATGCGATAAGTAATGTGAATTGCAGAATTCAGTGAATCATCGAATCTTTGAACGCACATTGCGCCCGCCAGCATTCTGGCGGGCATGCCTGTTCGAGCGTCATTTCAACCATCAAGCCCCCGGGCTTGTGTTGGGGACCTGCGCGGCTCTTTTCTCGCCCGCAGGCCCTGAAATGCAGTGGCGGGCTCGCTAGTCACACCGAGCGTAGTAGCATTTTTGTCTCGCTCAGGGCGTTGCTGCGGGTTTCCCGGCCGTTAAACACCCACCATTT-CTCAAGGTTGACCTCGGATCAGGTAGGAAGACCCGCTGAACTTAAGCATATCAATAAGCGGAGGAAAAGAAACCAACAGGGATTGCCCTTAGTAACGGCGAAGTGAAGCGGCAACAGCTCAAATTTGAAATCTGGCCTCGGCCCGAGTTGTAATTTGCAGAAGGAAGCTTTGGGCGCGGCGCCTTCCGAGTCCCCTGGAACGGGGCGCCATAGAGGGTGAGAGCCCCGTATGGTTGGACGCCTAGCCTGTGTAAAGCTCC-TTCGACGAGTCGAGTAGTTTGGGAATGCTGCTCAAAATGGGAGGTAAATTTCTTCTAAAGCTAAATACCGGCCAGAGACCGATAGCGCACAAGTAGAGTGATCGAAAGATGAAAAGCACTTTGAAAAGAGGGTTAAATAGCACGTGAAATTGTTGAAAGGGAAGCGCTTGTGACCAGACTTGCGCCGGGCTGATCATCCGGTGTTCTCACCGGTGCACTCTGCCCGGCTCAGGCCAGCATCGGTTCTCGCGCGGGGACAAAGGTCCCGGGAATGTAGCTCCTCCGGGAGTGTTATAGCCCGGGGCGCAATGCCCGCGCGGGGACCGAGGCTCGCGC-TCTGCAAGGATGCTGGCGTAATGGTCATCAGCGACCCGTCTTGAAACACGGACCAAGGAGTCAAGGTTTTGCGCGAGTGTTTGGGTGTCAAACCCGCACGCGTAATGAAAGTGAACGTAGGTGAGAGCTTCGGCGCATCATCGACCGATCCTGATGTATTCGGATGGATTTGAGTAGGAGCGTTAAGCCTTGGACCCGAAAGATGGTGAACTATGCTTGGATAGGGTGAAGCCAGAGGAAACTCTGGTGGAGGCTCGCAGCGGTTTCT

GACGTGCAAATCGATCGTCAAATCTGAGCATCTGGTCTCACTGGTGCAGGGTTTGCGGAGAAAGAATGTCATTTCGTTTGAGGTTTCGCTT
GTCCGGGACATTCGCGATCGCGAGTTCAAGATCTTTTCGGATGCTGGCCGCGTCATGAGGCCTCTGTTTACTGTGGAACAAGAGGACAAC
AGCGACACCGGCGCCGAGAAAGGCCAGCTGGTCCTAAATAAGGAGCACATCCAACGGCTAGAGAGGGATAAGGAGCTCGGCAAATAC
CACCCCGACTATTGGGGCTGGCCTGGGCTGTTGAAGTCGGGCGCGATTGAATACCTCGACGCTGAGGAAGAGGAAACGGCCATGATCAG
CATGAGTCCCGAGGATCTCGACATGTTCCGGCTCCGCAAGCTGGGCTTCACCGTCGAAGACCATTCCGGCGAAGGCAATAGCCGAATCA
GGACCAAGCCGAACCCGGCGACTCACATGTACACGCACTGCGAGATCCATCCCAGCATGCTTCTCGGCATCTGCGCGAGTATTATCCCAT
TCCCCGACCACAACCAGGCGTCTCGGGAAGCTATATTTTT-TGGGCCCCTGACC--TTACCC--CACACTTTTTGA-ACAATACGCG---CC-
CAAGCT--CCCAGA----CCGACTG--GAAAGGCGATGCCGATGCTCTCTTGA--TGGGAT-----CGACACG-ATACTAACTA--
TGGTTCTCTGCTATAGGTTCACCTTCAGACTGGCCAATGCGTAAGTTCGACCGATTC--GAGC---GTCGGCGGCCTTATCAGGTGG----
TGTGGTGC------AGGTG--GACTGACAACAACCCGATGCAGGGTAACCAAATTGGTGCCGCTTTCTGGTATG-GCCA--
TCCTCAAGATTCAGCAC-----CATCGCCCAAA-
AGCCACTTCTTGCTGACTTATTTGACAGGCAGACTATCTCCGGCGAGCACGGCCTCGACGGCAATGGCGTGTACGTGGCTGCTGGCGATT
CCCG-GTCGGTTAA--TCCC---TCGCTCACCGTCCC-
GATAGGTATAATGGCACCTCCGAGCTCCAGCTCGAGCGCATGAACGTCTACTTCAACGAGGTAAGTTCGGATCCCAGATC--
TCCTGAGCGGTT-----GGAGTGGGAT-GTGCGCTGAAA---------
TCTGGTGCAGGCGTCCGGCAACAAGTATGTCCCTCGTGCCGTTCTGGTCGACTTGGAGCCCGGCACGATGGATGCCGTCCGCGCTGGTCC
CTTCGGCCAGCTCTTCCGCCCCGACAACTT
